# Emotions and individual differences shape human foraging under threat

**DOI:** 10.1038/s44220-025-00393-8

**Published:** 2025-03-12

**Authors:** Hailey A. Trier, Jill X. O’Reilly, Lisa Spiering, Sandy Ma Yishan, Nils Kolling, Matthew F. S. Rushworth, Jacqueline Scholl

**Affiliations:** 1 Department of Experimental Psychology, Wellcome Centre for Integrative Neuroimaging (WIN), University of Oxford, Oxford, UK; 2 University of Toronto, Toronto, Ontario Canada; 3 Université Claude Bernard Lyon 1, Inserm, Stem Cell and Brain Research Institute U1208, Bron, France; 4 Université Claude Bernard Lyon 1, CNRS, INSERM, Lyon Neuroscience Research Centre (CNRL) U1028 UMR5292, PsyR2 team, Centre Hospitalier Le Vinatier, Bron, France

**Keywords:** Psychology, Diagnostic markers

## Abstract

A common behavior in natural environments is foraging for rewards. However, this is often in the presence of predators. Therefore, one of the most fundamental decisions for humans, as for other animals, is how to apportion time between reward-motivated pursuit behavior and threat-motivated checking behavior. To understand what affects how people strike this balance, we developed an ecologically inspired task and looked at both within-participant dynamics (moods) and between-participant individual differences (questionnaires about real-life behaviors) in two large internet samples (*n* = 374 and *n* = 702) in a cross-sectional design. For the within-participant dynamics, we found that people regulate task-evoked stress homeostatically by changing behavior (increasing foraging and hiding). Individual differences, even in superficially related traits (apathy–anhedonia and anxiety–compulsive checking) reliably mapped onto unique behaviors. Worse task performance, due to maladaptive checking, was linked to gender (women checked excessively) and specific anxiety-related traits: somatic anxiety (reduced self-reported checking due to worry) and compulsivity (self-reported disorganized checking). While anhedonia decreased self-reported task engagement, apathy, strikingly, improved overall task performance by reducing excessive checking. In summary, we provide a multifaceted paradigm for assessment of checking for threat in a naturalistic task that is sensitive to both moods as they change throughout the task and clinical dimensions. Thus, it could serve as an objective measurement tool for future clinical studies interested in threat, vigilance or behavior–emotion interactions in contexts requiring both reward seeking and threat avoidance.

## Abstract (web summary)

This study explores the impact of mood and individual differences on trading off between possible rewards and checking for the presence for threat and escaping to safety in a gamified foraging task.

## Main

To survive in natural environments, animals must balance their time between pursuing essential rewards, such as food, while being vigilant for potential threats, such as predators. Humans also rely on this type of decision-making in contemporary life. For example, care must be taken to avoid cars when entering the supermarket to buy food. Examining how people partition their time and switch flexibly between foraging and vigilance may help us understand not only a fundamental decision-making process that is ubiquitous across species but also how it is shaped by individual human traits and emotions.

The study of cognitive mechanisms underlying reward- and threat-oriented behavior has been advanced by paradigms in which single-event decisions are presented to participants at discrete intervals. However, recently a new framework, computational ethology, has emerged for studying and analyzing natural, everyday decision-making with ecological tasks^1–4^. One key paradigm is the ecological foraging task. Previous studies have described cognitive and neural mechanisms associated with foraging in environments with fluctuating rewards^1,5–7^. However, it is not well understood how decision-makers manage foraging in the context of predatory threat. Risk of predation is a constraint on animal foraging behavior, and survival requires foraging strategically to evade threat. Animal, human and modeling studies have modeled the predatory risk versus foraging trade-off and characterized the effects of environmental conditions on evasive behavior and value-based decision-making.^8–12^ Work has also begun to look at individual differences (for example, gender and clinical traits) in the coordination of reward-seeking and threat-avoiding behaviors.^13,14^ The key aim of our study is looking at how humans partition their time among foraging, vigilance and escape behaviors. We examine foraging under predatory threat, with a special focus on how this both reflects and drives variation in momentary emotions.

Ecological tasks may be particularly useful for studying not just fundamental aspects of everyday and healthy behavior but also behavior that is changed in poor mental health. The current task makes it possible to examine how changes in the balancing of reward-seeking and threat-related behaviors are linked with transdiagnostic behavioral dimensions, that is, aspects of behavior that cut across traditional diagnostic categories^15^ associated with generalized anxiety disorder (dimension: anxiety and intolerance to uncertainty), obsessive–compulsive disorder (OCD, dimension: compulsive checking), and depression (dimensions: anhedonia and apathy). We hypothesize that these dimensions are either related to the processing of threat (anxiety, intolerance to uncertainty, compulsivity) or reward motivation (apathy, anhedonia).

Anxiety has been characterized in terms of threat-related attentional bias, threat avoidance, changes in information sampling and learning.^16–21^ However, it is not known how anxiety affects behavior in the context of predatory threat, in particular, when vigilance can reveal danger but also help evade it. For disorders involving apathy and anhedonia (for example, depression), a range of studies have revealed differences in reward-guided behavior, in particular changes to the trade-off between reward and effort and changes to reinforcement learning.^22–28^ It is not yet clear how this translates to more ecological tasks requiring switching between vigilance and foraging, or whether, in a more ecological task scenario, extreme variation might even enhance performance (and we find here that indeed it does), as suggested by evolutionary psychiatry.^29^ One psychiatric dimension that has previously been examined under this lens concerns the symptoms of psychosis which, while associated with severe psychiatric illness (for example, schizophrenia) also correlate with effects that are experienced as positive, such as mystical experiences^30^ or creativity.^31^

In addition to the influence of individual traits, everyday behavior is subject to changing moods, and reciprocally, behavior may, in turn, drive variation in those moods. For example, it is very intuitive that when feeling stressed, one would choose behaviors to reduce this stress. Studies using reinforcement learning and decision-making paradigms have shown that emotions (more specifically, ‘happiness’) are impacted by reward outcomes, and in turn, emotions affect learning and decision-making in several ways.^32–35^ While recent computational models have highlighted how distinct emotions may affect distinct behaviors, there is so far less experimental work examining this.^36,37^ In this Article, we will address this by capturing two distinct emotions, stress and excitement. We focus on these emotions as particularly relevant for a task involving the two dimensions of threat and reward. Second, we investigate the possibility of a bidirectional, homeostatic relationship between emotion and behavior. For example, a daily life mood-monitoring study found that individuals engage in pleasurable activities during low moods and shift to useful but less pleasant tasks during good moods.^38^ This homeostasis was impaired by depression.^39^ We build on this by measuring computational effects of mood as it changes throughout the task and its homeostasis in a well-controlled experiment.

In summary, the task made it possible to examine how people apportion their time between pursuing rewards and checking for threats, how these decisions unfold moment to moment and lead to changes in emotion and arousal, and how these processes vary in relation to a range of clinical dimensions. We employed a split-sample approach^23^ recruiting two large samples (discovery: *n* = 374; replication: *n* = 702). We pre-registered hypotheses (summarized in Table 1 and 2) on the basis of the discovery sample (https://osf.io/3gb8n). We find that stress shows a homeostatic relationship with foraging (providing pleasant rewards) and hiding. We find our most striking links to real-life individual differences for gender (with women performing worse, checking too much and foraging too little) and for apathy, with higher apathy being linked to an improvement in performance due to fewer unnecessary behavioral switches between foraging and checking.

**Table 1:** Mood and task performance

Hypothesis	Eff. size [lower CI]	*P* (one-tailed)	*W* or *t*	*n*
1. Environment is linked with mood.	0.79 [0.50]	<0.001	68,189	377
1A. Environment affects excitement.	0.43 [0.09]	<0.001	182,380	699
1B. Environment affects stress.	0.76 [0.30]	<0.001	228,961	699
1C. Having been caught affects stress and excitement in opposite ways.	0.23 [0.02]	<0.001	NA	377
2. Stress and behavior have homeostatic interactions.	0.21 [0.13]	<0.001	116,410	612
2A. Stress has a homeostatic relationship with foraging behavior before discovery of the predator.	0.19 [0.10]	<0.001	134,216	662
2Ai. Stress is linked with increased foraging and more efficient checking before the predator is seen.	0.14 [0.06]	<0.001	140,444	696
2Aii. Foraging and efficient checking are linked with reduced stress (both pre- and post-discovery).	0.27 [0.03]	<0.001	144,016	662
2B. Stress is linked with a behavioral shift toward hiding rather than activity.	0.11 [0.02]	0.003	116,043	641
3. Excitement and action vigor are in a positive feedback loop.	0.04 [0.00]	0.125	1.15	689
3A. Excitement is linked with increased response vigor.	–0.01 [–0.01]	0.62	–0.31	699
3B. Response vigor is linked with increased excitement.	0.11 [0.00]	0.002	2.97	690

Our task evoked changes in stress and excitement (1A–C). Stress showed a homeostatic relationship with behavior (2A, B). By contrast, a positive feedback loop between excitement and behavior was not replicated (3A, B). All hypothesis tests reported here reflect a single Wilcoxon signed-rank test, with the following exceptions: H1C is the least significant *P* value of two Wilcoxon signed-rank tests, and H3, 3A and 3B were all independent sample *t* tests. All tests are one-tailed. All predictors and their individual significance levels are listed in Supplementary Table 9. Eff. size, Cohen’s *d* for *t* tests and Wilcoxon effect size for Wilcoxon signed-rank tests. CI, confidence interval—only lower boundaries are reported as pre-registered tests are one-tailed. Reported test statistics: *t* statistic for *t* tests or *W* statistic for Wilcoxon signed-rank tests. *n*, sample size available for tests (differing between tests depending on data available per participant; for example, for hypothesis 1C, some participants got caught too rarely in the task to allow measurement of the impact of this on mood).

**Table 2:** Individual differences in clinical traits and gender correlate with task behavior in the replication sample

Hypothesis	Pearson’s *r* or % correct [CI]	*P* (one-tailed)	*n*
4. OCI-RC score is associated with measures of task-related behavior.	0.18 [0.12;]	<0.001	670
4A. OCI-RC score is associated with increased vigilance and decreased foraging, but in a disorganized pattern.	0.161 [0.10;]	<0.001	673
4Ai. OCI-RC score is associated with increased vigilance.	0.014 [-0.05;]	0.35	690
4Aii. OCI-RC score is associated with increased disorganized checking.	0.223 [0.16;]	<0.001	701
4Aiii. OCI-RC score is associated with decreased foraging, particularly when reward is low.	0.042 [-0.02;]	0.15	681
4B. OCI-RC score is associated with greater threat avoidance.	0.16 [0.10;]	<0.001	702
4C. OCI-RC score is related to higher reported arousal (stress and excitement) in general and in anticipation of predator arrival.	0.12 [0.06;]	0.0011	699
5. Anxiety and intolerance to uncertainty are related to measures of task-related behavior.	0.069 [NA;]	0.036	678
5A. Intolerance to uncertainty (IU) is associated with a change in the balance of check/forage/hide actions in response to both environment and mood.	0 [NA;]	0.5	678
5Ai. Prospective IU is associated with a reduction in the tendency for reward to shift behavior toward foraging instead of vigilance.	0.03 [-0.03;]	0.22	681
5Aii. Inhibitory IU is linked with changes in the effect of predator speed and stress on hiding behavior.	-0.018 [-0.08;]	0.69	700
5Aiii. Prospective IU and cognitive/somatic anxiety are associated with increased emotional responses to reward and threat.	-0.02 [-0.08;]	0.7	699
5B. Trait-somatic anxiety is linked with decreased vigilance due to threat-related worry and avoidance, resulting in being caught more often.	0.205 [0.14;]	<0.001	697
6. Apathy and anhedonia are linked with measures of task-related behavior.	0.123 [NA;]	<0.001	696
6A. Behavioral apathy is associated with a reduction in vigilant and threat-avoidant behavior in favor of foraging, despite increased emotional sensitivity to threat.	0.106 [0.04;]	0.0031	696
6Ai. Behavioral apathy is associated with a reduction in vigilant and threat-avoidant behavior in favor of foraging (resulting in higher final score).	0.085 [0.02;]	0.0114	698
6Aii. Behavioral apathy is linked with increased self-reported emotional sensitivity to threat.	0.073 [0.01;]	0.0257	700
6B. Anhedonia and emotional apathy are linked with the foraging/vigilance trade-off being more sensitive to reward, while reducing the subjective feeling of task engagement.	0.141 [0.08;]	<0.001	696
6Bi. Anhedonia and emotional apathy are linked with the foraging/vigilance trade-off being more sensitive to reward.	0.055 [-0.01;]	0.07	699
6Bii. Anhedonia and emotional apathy are associated with a reduction in the self-reported feeling of task engagement.	0.165 [0.10;]	<0.001	699
7. Gender is linked with task performance.	66% [NA;]	<0.001	659
7A. Women overall perform worse. They tend to favor vigilance over foraging.	65% [NA;]	<0.001	662
7Ai. Women forage less.	66% [NA;]	<0.001	671
7Aii. Women check more and sooner, but also get caught more often.	60% [NA;]	<0.001	678
7B. Women are more stressed in general and particularly by threat.	54% [NA;]	0.0183	689
7C. Women show lower response vigor, particularly in situations where it is usually increased.	63% [NA;]	<0.001	691

Correlations (Pearson’s *r*) or accuracy (% correct classification of binary gender (male/female)) were computed by predicting clinical scores (or gender) in the replication sample on the basis of the regression models trained on the discovery sample. Results for individual measures making up the hypotheses are shown in Supplementary Table 11. *P* values are the result of one-tailed permutation tests. CIs are also one-tailed; they are NA for accuracy (gender) as the value present is the percentage of participants correctly classified. When overarching hypotheses were the combination across different questionnaire subscales (H5 and H6), confidence intervals were not defined (NA) and statistics were computed on the average correlation across the sub-hypotheses (see Methods). See Supplementary Table 12 for additional statistics (Bayes factor (BF), out-of-sample *R*^2^ and a control analysis of uniqueness of the links).

## Results

### Participants

A total of 1,199 participants took part after giving informed consent. Data quality checks excluded 123 participants due to inattentiveness (during the task or the questionnaires), incompleteness or otherwise low-quality data (Supplementary Figs. 1 and 2 and Supplementary Table 1). This resulted in 1,076 (discovery sample: *n* = 374; replication sample: *n* = 702) with a good spread of clinical scores (for example, 27% with clinically significant anxiety scores or 16% taking psychoactive medication, mainly antidepressants; Supplementary Tables 2 and 3). Here we report results from testing the replication sample with both pre-registered and exploratory analyses (discovery sample results are included with the pre-registration).

### Task

**Fig. 1:**
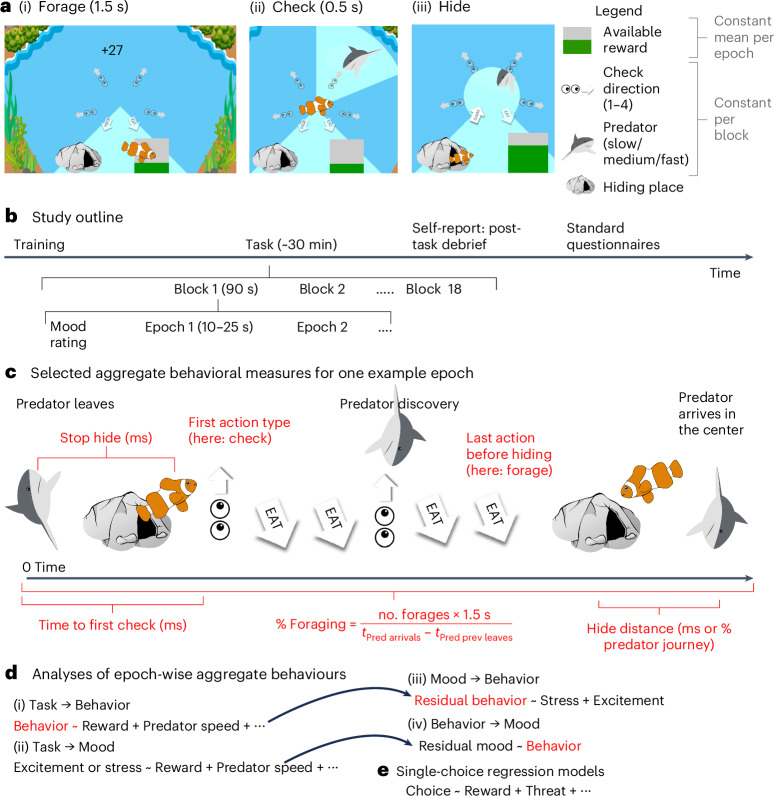
Experimental task. **a**, In the task, participants could freely choose to forage (**a**(i)) to gain points, check for predator threats (**a**(ii)) or hide (**a**(iii)) from threat. Predators (differing in three levels of approach speed) appeared at a randomized location at the edge of the game area after a variable delay and moved toward the fish until either catching it or retreating. When arriving in the center, the predator caught the fish if it was not hidden. Being caught decreased participants’ life counter by one. **b**, Study outline. The task comprised 20 blocks of 90 s varying in the type of predator (slow, medium or fast) and the number of check areas (one to four). Within a block, there were several predator epochs (see **c** for example), which varied in average reward rate. Between blocks, participants used sliding scales to rate their level of excitement and stress during the previous block. **c**, Epochs lasted from the disappearance of the previous predator (or the beginning of a block) until the arrival of a predator in the center of the screen. In red are highlighted some aggregate behavioral measures that we computed (Supplementary Table 5). **d**,**e**, The data were analyzed using different types of regression models: using behavioral measures averaged across epochs (**d**(i)) or blocks (**d**(ii)–**d**(iv)) or using non-averaged (that is ‘single’) choices (**e**). Taking advantage of the interleaved nature of mood ratings and task blocks, we could establish directionality in the impacts of the task on mood (**d**(ii)), of mood on behavior (**d**(iii)) and of behavior on mood (**d**(iv)).

During each of 20 90-second blocks, participants played a fish in an underwater environment (Fig. 1) in which they continuously and freely chose between foraging for rewarding food (later translated into bonus payment; Fig. 1a(i)), checking for threatening predators (Fig. 1a(ii)) and hiding in a safe space (Fig. 1a(iii)). Their goal was to gain as much reward as possible without being caught by any predators. Participants thus needed to trade-off opportunities for foraging for rewards with the need to check for predators (incurring an opportunity cost as they could only either check or hide, not do both at the same time) to be able to escape threat in time (again incurring an opportunity cost as no foraging was possible while hiding). Within each block, three to six predators would approach the fish; the block could therefore be subdivided into ‘predator epochs’ comprising first a variable period with no predator (2.0 to 10.5 s) then an approach period in which the predator started approaching from the edge of the screen toward the center in a straight line at a constant speed, taking 10, 15 or 20 s (that is, three speed levels) to reach the center. The ‘checking segment’ in which the predator appeared was randomized. Once the predator had entered the environment, the predator would be detected if the participant chose to check that segment. Finally, there was an endpoint when the predator reached the center, at which point it caught the fish (if foraging) or not (if hiding). Blocks varied by predator threat level (speed of the predator, three levels) and the number of segments to check (one to four), which determined the number of checks required to scan the entire surrounding area for predators. Reward richness varied between predator epochs: when foraging, the participants received a series of slightly variable rewards around a set mean within each predator epoch; reward size was random about this mean and did not deplete with consumption. In between the 90-second blocks, participants rated their level of excitement and stress during the previous block using sliding scales (Fig. 1b, ‘mood rating’). After the task, participants completed psychiatric questionnaires measuring symptoms of apathy,^40^ anhedonia,^41^ anxiety,^42^ intolerance to uncertainty,^43^ compulsive checking,^44^ decentering ability^45^ (included only in exploratory analyses) and answered questions that required them to introspect about their task performance (‘post-task debrief’; Supplementary Table 4).

### Behavioral measures

**Fig. 2:**
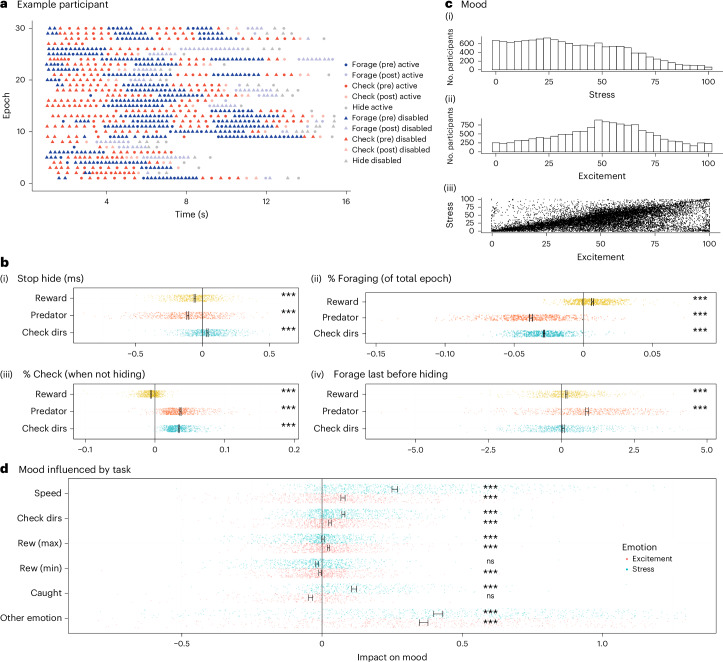
Behavioral measures. **a**, Example participant behavior throughout the epochs for the fast predator (more examples in Supplementary Fig. 3). **b**, Task features impacted behavioral measures: time until participants pressed a button to return from hiding (**b**(i)), proportion of time in each epoch spent foraging (**b**(ii)), proportion of active (non-hiding) time spent checking (**b**(iii)), whether the last action before hiding was a forage (coded as 1) or not (coded as 0) (**b**(iv)), measured in regression analyses. **c**, Ratings of stress (**c**(i)) and excitement (**c**(ii)) correlated across participants (**c**(iii); average Pearson’s *r* = 0.68, *P* < 0.001). Mood was impacted by task factors of the preceding block (regression analyses). Predator, fast, medium or slow speed of the predator; Check dirs, the number of possible directions to check; Rew (max)/Rew (min), maximum/minimum reward in the 90 s block (consisting of several epochs, with constant mean reward within each epoch); Caught, number of times caught by predator previously; Other emotion, stress when predicting excitement and vice versa. Significance tests: two-tailed (**b**) and one-tailed (**d**) one-sample *t* tests (other than for the impact of ‘other emotion’ in **d**, which was two-tailed as not pre-registered) of the regression weights of individual participants. ***P < 0.001. In **d**, for illustration, axes were constrained, cutting off some data points (<34 out of 699). Error bars show mean and standard errors. Individual participants are shown as dots. All data from replication sample (**b**: *n* = 702; **d**: *n* = 699 other than regressor ‘caught,’ for which *n* = 377).

This task provided rich measures of behavior: participants varied widely in their strategies for approaching the task (Fig. 2 and Supplementary Fig. 3). We analyzed the behavior, deriving measures taking account of this variability (Fig. 1c,d). Task data were epoched into two separate ‘phases’ for analysis: behavior before a predator had been discovered (‘pre-discovery phase’) and after (‘post-discovery phase’). For each epoch, we extracted aggregate behavioral measures that we then related to task features (for example, how much reward affects the rate of checking or the time to first action in an epoch; Fig. 1d). The task features of key interest were the predator speed and the reward, while we also controlled for other features, including the number of areas to check and the block in the experiment (thereby controlling for performance improvements over time; Supplementary Fig. 4). This provided us with a very rich set of measures with robust links to individual differences on clinical questionnaires. Specifically, 33 behavioral measures (Supplementary Table 5) were extracted from task data (Fig. 1c) related to hiding (for example, how long participants took to return from hiding after disappearance of the predator), foraging (for example, percentage of total time in an epoch spent foraging) or whether the last action before hiding was a forage or a check), or checking (for example, percentage of active (non-hiding) time spent checking) or the interplay among the task, mood (Fig. 2c) and behavior. We then related each behavior to task factors such as reward and threat levels (Fig. 2b). Many measures were affected by changes in reward, threat and number of directions for checking (Supplementary Table 8 has a complete list). For example, when reward was higher, participants returned faster from hiding (*P* < 0.001) and spent more time foraging (*P* < 0.001) and less time checking (*P* < 0.001). When predators were faster, participants were equally faster to return from hiding (*P* < 0.001) but spent less time foraging (*P* < 0.001) and more time checking (*P* < 0.001). In addition, in exploratory analyses, we also used more standard individual-choice decision-making models^46^ (Fig. 1e) that applied logistic regressions to each choice participants made (thus not capturing reaction time or mood effects). We show group-level results (Supplementary Table 6), clinical links in exploratory analyses (following) and parameter recovery (Supplementary Table 7 and Supplementary Fig. 5).

### Testing pre-registered hypotheses

On the basis of the discovery sample results, we pre-registered hypotheses to then test in the replication sample. We tested three groups of pre-registered hypotheses (labeled H1 to H7 and where sub-hypothesis are labeled, for example, as H1A) relating task behavior with (1) mood states, (2) clinical scores and (3) gender. Hypotheses were pre-registered hierarchically (power calculations in Supplementary Fig. 6), with more general ones being tested before more specific ones, using follow-up tests only if the more general ones were significant. In other words, statistical significance testing was performed and inferences made about the general-level hypotheses first. Only when a general-level hypothesis was significant was statistical significance testing and inference at the lower levels warranted. This approach ensured that the number of false positives was kept low because it was similar to the familiar method of initially performing an omnibus test, such as an analysis of variance, and only subsequently conducting follow-up tests. All hypotheses were formed on the basis of the findings in the discovery sample and then tested in the independent replication sample. This approach allowed detection of unexpected results while also incorporating robust statistics.

#### Mood hypothesis test results

##### Mood is influenced by environment

Mood ratings (stress and excitement) varied substantially across the experiment (Fig. 2c). Our first set of hypothesis tests (H1) confirmed that task-related environmental factors (for example, threat level, reward) affected mood ratings (H1; *Z* = –15.37, *P* < 0.0001; Fig. 2d). Examining this further, we found that environmental factors were significantly linked with excitement (H1A; *Z* = –11.21, *P* < 0.0001; Fig. 2d) and stress (H1B; *Z* = –19.97, *P* < 0.0001; Fig. 2d): for both moods, higher ratings were coupled with higher rewards, faster predator speed (threat level) and greater number of directions to check for predators. However, being caught by a predator was associated with stress and excitement in different ways (H1C; least significant *P* value of two component measures was *P* < 0.001): being caught was linked with increased stress and decreased excitement. Of interest here is evidence that mood effects may vary by valence; while stress and excitement both constitute emotional arousal, they are associated with negative and positive valence, respectively. Here we found that some threat-related factors affected arousal regardless of valence (for example, faster predator speed increased both stress and excitement) while others had a valence-specific effect (for example, being caught). In an exploratory analysis, we also assessed the relationship between the excitement rating and the stress rating, which showed a positive relationship across participants (Fig. 2c(iii),d, *P* < 0.001); that is, the higher the excitement ratings, the higher the stress ratings.

##### Mood and behavior are homeostatic

**Fig. 3:**
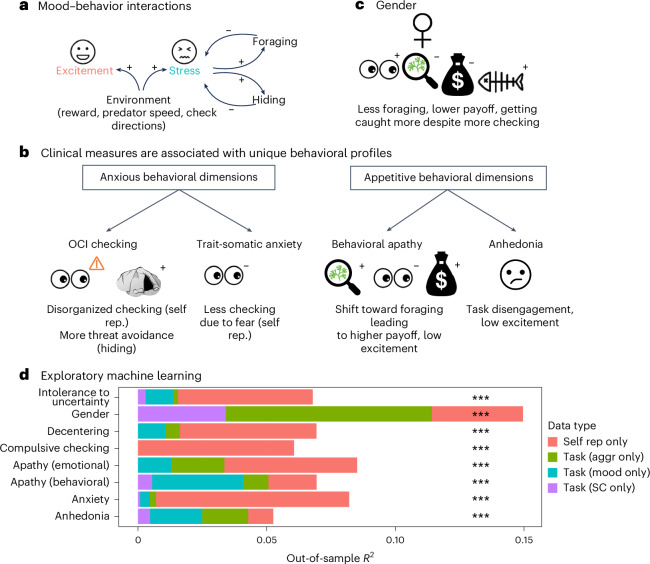
Results summary and exploratory analyses. **a**, The task evoked emotions. Participants regulated emotions through behavior homeostatically: pre-block stress led to increased foraging and hiding, which in turn lowered stress ratings after the block (impact of hiding on stress not pre-registered). **b**, Individual differences (questionnaires, gender). Compulsive checking (OCI-R) was linked with disorganized and sub-optimal self-reported checking, greater threat avoidance via hiding and task disengagement. Trait-somatic anxiety (STICSA) was related to a self-reported reduction in checking due to threat-related worry, self-reported checking avoidance. Behavioral apathy (AMI behavioral score) was associated with a shift in behavior toward foraging and away from checking, leading to a higher total payoff at the end of the task. Apathy was also associated with reduced excitement (total payoff and excitement results not pre-registered). Anhedonia (SHAPS score) was linked with self-reported task disengagement and lower excitement, but if anything, increased behavioral sensitivity to reward. **c**, Women performed worse in the task overall (reduced payoff). They foraged less, checked more and got caught more often. **d**, Exploratory machine-learning models (regularized Bayesian regression) trained on the replication sample could predict clinical scores and gender in the discovery sample when trained on different groups of measures (for illustration, out-of-sample *R*^2^, all *P* < 0.001; Extended Data Table 1): post-task self-reports of task behavior (‘self rep.’), behavior during the task split into measures (for example, rate of checking or impact of reward on rate of checking) relating to either (1) mood (‘mood’) or (2) non-mood aggregate behaviors (‘aggr’) and (3) obtained from single-choice regression analyses (SC), similar to standard decision-making models. While for many clinical dimensions, self-reports provided the largest explanatory power, this was notably less the case for apathy, anhedonia and gender. In **d**, *n* = 374 (model trained on replication sample *n* = 702).

A second set of hypothesis tests (H2) investigated possible homeostatic interactions between stress and task-related behavior, that is, whether an interdependent relationship exists between stress and behavior by which each contributes to regulating the other (that is, a negative feedback loop). Homeostatic relationships should, therefore, mean that the stress-induced behavior leads, in turn, to a reduction in stress. Such a result would be analogous to the previous findings that people engaged with pleasant behaviors during low moods, which in turn improved mood.^38^ To test for causality, we took advantage of the temporal order of measurements; that is, to measure the impact of mood on behavior, we used regression predicting behavior in the task as a function of previous mood. Conversely, to measure the impact of behavior on mood, we used regression predicting mood ratings made after a block, based on behavior during a block, controlling for mood preceding the block. Indeed, we found general evidence of such interactions (H2; *Z* = –4.90, *P* < 0.0001; summary in Fig. 3a). First, we confirmed a homeostatic relationship between stress and foraging behavior (H2A; *Z* = –4.85, *P* < 0.0001). Increased stress preceded increased foraging and more efficient checking (H2Ai; *Z* = –3.46, *P* = 0.0003). This was captured by individual measures (Supplementary Table 9) such as stress increasing pre-discovery foraging rates (*P* = 0.04) or decreasing the proportion of active (non-hiding) time spent checking (*P* = 0.02) or leading to earlier detection of the predator (*P* < 0.001). Conversely, we found that foraging and efficient checking behaviors significantly reduced stress (H2Aii; *Z* = –6.98, *P* < 0.0001). This was captured by findings such as that higher forage rates before discovery of the predator (*P* < 0.001), or the presence of any foraging after discovery of the predator (*P* < 0.001), decreased stress (full list of replicated measures in Supplementary Table 9). In addition, greater stress was linked with a behavioral shift toward hiding rather than activity (H2B; *Z* = –3.45, *P* < 0.001; individually replicated measure: stress leading to earlier hiding, *P* < 0.001; Supplementary Table 9). Because hiding had no effect on stress in the discovery sample, we did not pre-register to examine this further in the replication sample. However, in an exploratory analysis, combining data across the two samples, we found some evidence that earlier hiding reduced stress in subsequent blocks (*P* < 0.001, BF = 19 for the impact on stress two blocks into the future and *P* = 0.01, BF = 0.8 for the impact on stress directly measured after the behavior).

We failed to replicate a finding in the discovery sample of a positive feedback loop between excitement and action vigor (H3, *t*(688) = 1.15, *P* = 0.125). As an exploratory analysis, we report the results for the separate parts of the loop between excitement and action vigor in Table 3, which found a significant link of action vigor increasing excitement (*P* = 0.002), but not excitement increasing action vigor (*P* = 0.62). This was captured by (as a proxy for vigor) the rate of pressing of the hide button (*P* = 0.03) or the forage button (*P* = 0.002) when they were inactive (the buttons were inactive for a short delay after each response although button presses were still recorded during this period) increasing excitement. Note that for H2A, H3A and H3B, the effects are specific to the reported emotion (stress or excitement), as tested by including the other emotion as a control factor. By contrast, for H2B, the effect does not remain significant when controlling for excitement (*P* = 0.19), suggesting an effect due to a more general effect arousal rather than an effect specifically related only to stress (test for excitement only: *P* = 0.0018, but in discovery sample: *P* = 0.51; test for excitement controlling for stress: *P* = 0.017, but in discovery sample: *P* = 0.89).

**Table 3:** Behavioral, mood or self-report measures with links to clinical traits

Clinical subscale	Conceptual description	Variables
Apathy (behavioral)	Increased and more effective task engagement	↑ Total earnings, ↓hiding (predator distance when hiding) (regression model); ↑ % time spent foraging (regression model); ↑ probability of continued versus interrupted check sequence (single-choice regression model); ↑ rate of pressing inactive hide button (vigor), ↓Hiding (% time spent hiding (regression model), predator is already closer when hiding (regression model), ↓ proportion hide versus not hide actions (single-choice regression model), ↓ time to return from hiding (average)); ↑ modulation of % time spent hiding by threat (regression model)
	Self-reported reduced time spent checking	Post-task self-report: ↓ time spent checking
	Self-reported reduced excitement	Emotions during the task:↓ excitement
Anhedonia	Self-reported reduced task engagement	Post-task self-reports:↓ self-reports of feeling excitement and of feeling nervous when seeing the predator; ↓ trying to find the predator quickly, ↓trying to gather as much food as possible and time spent checking; emotions during the task: ↓ excitement
	Increased foraging at high reward levels	↑Impact of reward on % foraging and on % hiding in regressions (data visualization: ↑foraging and ↓hiding especially when reward is high)
	Other	↓ time to return from hiding [reg.r model];
Compulsive checking	Reduced foraging and increased hiding	↓Foraging (% time (regression model and average), more transitions from foraging to checking (regression model), shorter forage sequences (regression model)); ↑ later to return from hiding (average)
	Self-reported disorganized checking	Post-task self-report:↑ not checking to avoid having to make a decision, ↑ checking again (even though just checked)
	Self-reported increased vigilance and hiding (threat avoidance)	Post-task self-report: ↑taking longer breaks (before and after a block with higher threat because tired)
	Reduced correlation between excitement and stress during task	↓ Correlation excitement and stress during the task
Anxiety (somatic)	Self-reported reduced checking due to fear	Post-task self-report: ↑ nervous after seeing predator, ↑ finding threat distracting, ↑ avoid checking to avoid having to make a decision
	Self-reported reduced foraging due to threat	↑Reducing effort during stressful rounds, ↑ no foraging until predator discovery
	Reduced foraging	↓ Start epoch foraging (time to first forage (average), probability of foraging at all before predator discovery (average))
Intolerance to uncertainty (prospective)	Increased self-reported stress	Post-task self-report: ↑ finding predators distracting, ↑ trying to find predators as soon as possible, ↑ hiding earlier because stressed, ↑ stress after seeing predator, ↑ nervous after seeing predator
	Decreased modulation of behavior by reward	↓ Impact of reward in regressions on: % foraging, proportion of forage versus check choices (single-choice regression model), % checking (data visualization: absence of usual increase in foraging when reward is high)
	Reduced foraging versus checking	↑ start checking sooner in epoch (regression model, average); ↓ Probabiltiy of foraging at all before predator discovery (average)
	Other	Check frequency before discovery has ↓ impact on excitement

A list of measures with at least moderate (BF > 3) links to clinical subscales, combining data across the replication and discovery sample. The types of measures were post-task self-report; behavioral measures during the task, including the impact of task on behavior assessed through regressions; the behavioral measures aggregated across all epochs and blocks or the intercept of the regressions; and the single-choice regression model; moods during the task, including impacts of the task on mood, impact of mood on behavior and impact of behavior on mood. Links were checked for all task measures that had less than 20% missing data and, where applicable, showed significance on the group level (for example, impact of reward on percentage of checking); as this was exploratory, no correction for multiple comparisons was applied. Results here are from regressions controlling for other categories of clinical subscales (but not for subscales within the same categories; see Methods ‘Follow-up tests on individual behavioral measures’; for example, controlling for anxiety, but not for anhedonia, when assessing significant links for apathy) as well as demographics.

As an exploratory analysis, given recent reports of changes in mood over the duration of tasks,^47^ we examined the impact of block on mood (which was included in all mood models as a control regressor). We found that, for both stress and excitement, ratings decreased (excitement: *P* < 0.001 for *n* = 365 in the discovery sample and *P* < 0.001 for *n* = 698 in the replication sample; stress: *P* = 0.004 in the discovery sample and *P* < 0.001 in the replication sample; Supplementary Fig. 4b(iii), two-tailed). To exclude the possibility that this result captured an effect of ‘settling into doing a task,’ we separately analyzed data from only the second half of the experiment (starting block 10). We still found the same effect (hierarchical models were used as otherwise there was not enough data, Bayesian 95% credible intervals, significance defined as excluding zero for the regressor of event index: discovery sample: stress: –0.05 [–0.07; –0.03], excitement: [–0.07; –0.03]; replication sample: stress: –0.05 [–0.07; –0.03], excitement: –0.05 [–0.06; –0.03]). This suggests an effect of time on arousal, rather than on positive mood per se. See Supplementary Table 10 for an alternative, reviewer-suggested analysis that led to the same results and conclusions for all mood-related hypotheses.

#### Clinical hypothesis test results

**Extended Data Fig. 1:**
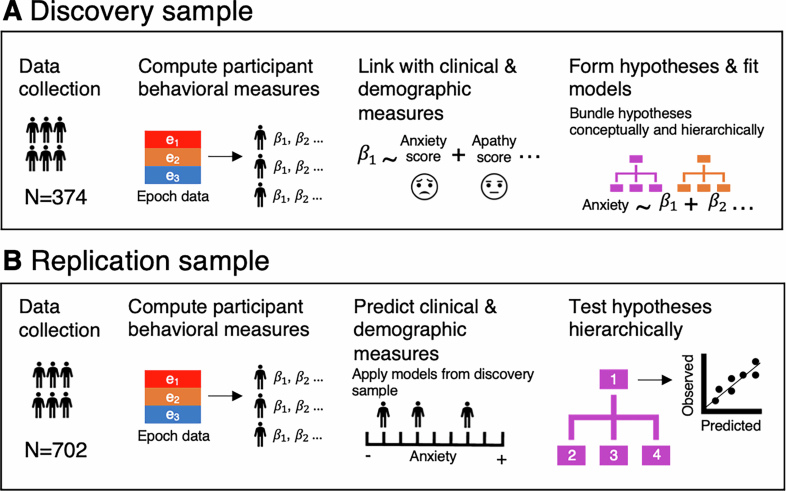
Principles of individual difference analyses. **A**) We extract task-based and post-task self-report measures for each participant in the discovery sample. For the behavior, we used epoched task data to regress environment factors (for example reward, predator speed) against task behavior (for example rate of foraging before discovery of the predator in each epoch). To identify unique links between real-life individual differences (questionnaire subscales and gender) and task behavior, we entered the measures as dependent variables in models where the independent variables were clinical scores and demographic measures. To establish hypotheses, we bundled significant findings by concept and arranged them hierarchically (from general to increasingly specific predictions). We expressed these hypotheses as regression models (clinical scores as dependent variables) and pre-registered them. **B**) To replicate, we collected an independent sample. We computed behavioral measures as for the discovery sample. To predict clinical scores and demographics for each person, we applied the hypothesis regression models fit on the discovery sample to the replication sample. We tested our hypotheses in a hierarchical manner (starting with the most general hypothesis first and proceeding to test its sub-hypotheses only if the general hypothesis was significant) by testing the goodness of our predictions. This meant, for example, that when many individual task measures could have been linked to a questionnaire subscale and were conceptually related to one another, their predictive power was combined in a single regression analysis, thus meaning that a single statistical test was used to test the superordinate hypotheses and only then follow-up tests were used to test subordinate hypotheses.

We investigated whether there was a replicable relationship between participants’ individual variation in traits across both healthy and clinically relevant ranges, as assessed by scores on psychiatric questionnaires, and their (self-reported) task behavior (H4–6). We did this in three ways. First, we pre-registered hypotheses combining several behavioral measures into conceptual groups (Table 2 and Supplementary Tables 11 and 12 for additional control and follow-up analyses). Second, we used an exploratory machine-learning approach (see the following). Third, we provided an exhaustive list of all associations found (Table 3). The pre-registered hypotheses were expressed as regression analyses, trained on the discovery sample and tested on the replication sample (Extended Data Fig. 1). These approaches together allowed detection of unexpected results (as were indeed found, especially for the apathy/anhedonia dimension) while also incorporating robust statistics. We replicated all pre-registered general hypotheses and many sub-hypotheses (Supplementary Table 11 has individual measures). However, we also noticed that while many compound hypotheses were significant, many individual, subordinate, measures were not, indicating that information was distributed across several measures and could most effectively be extracted using our compound hypothesis method.

**Extended Data Fig. 2:**
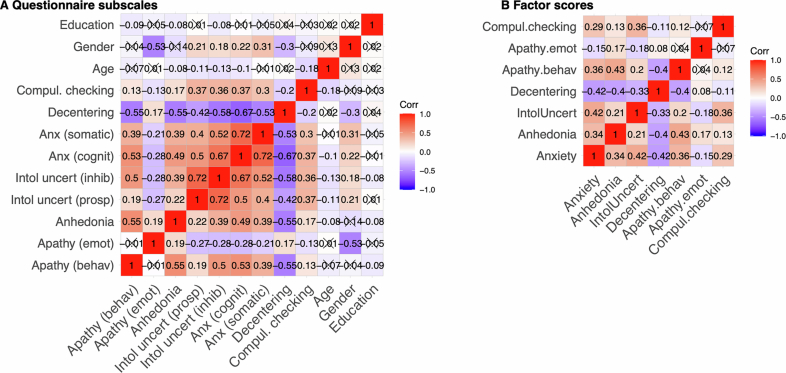
Correlations among questionnaire subscales and factors. Correlations (Pearson’s r) among the clinical subscales and demographics (**A**) and the factor scores (**B**) for the replication sample (non-significant correlations highlighted by an X). Note that for middle school education was scored zero, high school education as one, BSc [or equivalent] as two, MSc [or equivalent] as three, PhD as four). Sample size (values indicate the minimum per panel as pairwise complete observations used): **A**: n = 695 (for gender: 685, as omitting gender that was not female or male), **B**: n = 702 (as data imputed before factor analysis, thus including all participants).

**Extended Data Fig. 3:**
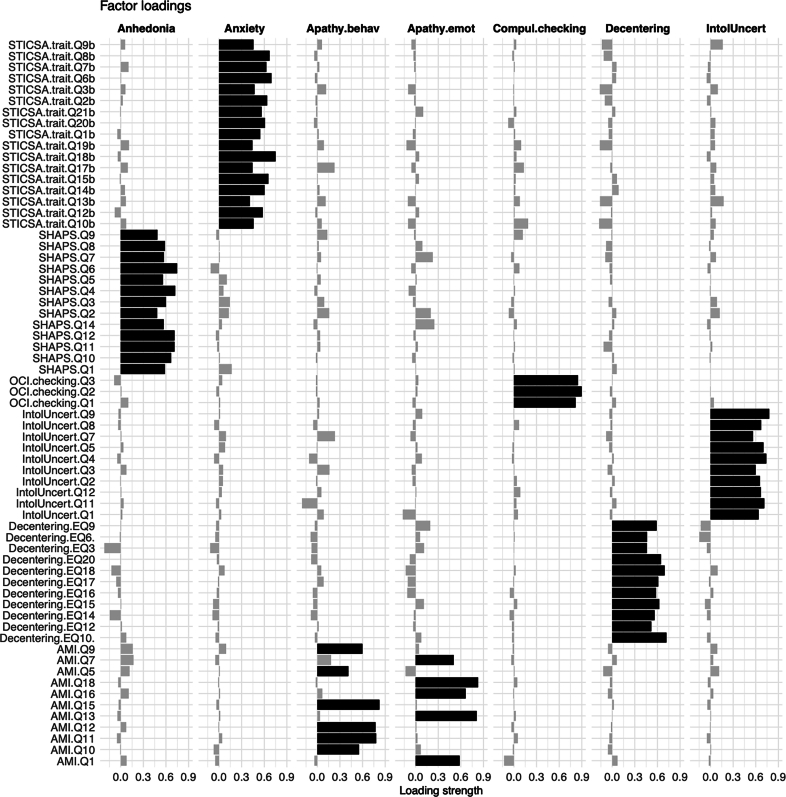
Factor loadings. Exploratory factor analysis (on the replication sample) revealed that for most questionnaires (anhedonia [SHAPS], compulsive checking [OCI], decentering, apathy [AMI]) the factors grouped the questionnaires together as intended in the questionnaires. The only exceptions are the anxiety (STICSA) and intolerance to uncertainty scales (IntolUncert), which grouped all items together, rather than separating them into separate factors. Abbreviations: questions for each questionnaires are counted as in the original papers (for example SHAPS.Q9 for question 9 from the SHAPS questionnaire). Confirmatory factor analysis in the discovery sample revealed moderately good fits (Comparative Fit Index: 0.808, Root Mean Square Error: 0.054), with no negative variance. All regression weights showed the expected direction for relating to the factors. See Extended Data Fig. 2 for correlations between the factors. N = 702

Before relating clinical subscales to the task measures, we inspected their correlations (Extended Data Fig. 2a). Especially within the anxiety measures, there were high correlations (for example, somatic to cognitive anxiety *r* = 0.72). But even between relatively distinct concepts (for example, behavioral apathy and somatic anxiety), associated with no overlapping questionnaire items, correlations were relatively high, and in fact higher than what we had observed in the discovery sample (Supplementary Fig. 7b). This could be partly explained by an accidental change in the age range of included participants (Supplementary Fig. 7c and Supplementary Table 12). A notable exception to this general pattern of positive correlations between clinical subscales was emotional apathy, which showed only weak, or even negative, correlations with other traits. Demographics, in particular gender, also showed correlations with clinical subscales (for example, women reported increased anxiety). To control for these correlations, we performed strict control analyses that allow looking at not only whether task-based measures are related to clinical subscales, but also whether this is unique to a specific subscale (Supplementary Table 12). Where results did not hold up to this strict correction, we note this below. On reviewer request, we also summarized separately clinical questionnaires with a factor analysis (Extended Data Figs. 2b and 3 and Supplementary Fig. 7a). For the questionnaires, this mostly reproduced the original questionnaire subscales, but with improved (that is, reduced) correlations between the dimensions (Extended Data Fig. 2b and Supplementary Fig. 7a), thus making controlling for different psychiatric dimensions easier. We explored links between these questionnaire factors and task measures using machine learning. We also replicated our findings (Supplementary Table 12) when excluding participants even more stringently for making any errors on the questionnaire check questions (*n* ≈ 290 participants), to correct for the relationship between making errors on the questionnaire check questions and higher psychopathology scores (Supplementary Fig. 2). Finally, correlations between all task measures used in the individual difference hypotheses are shown in Supplementary Fig. 8.

### Compulsive checking and response to threat

For compulsive checking (Obsessive–Compulsive Inventory (OCI) checking subscale), all overall hypotheses, but not all sub-hypotheses, were confirmed (Table 2), and several individual regressors making up the hypotheses also replicated (Supplementary Table 10). Compulsive checking was linked to a change in responses to threat (H4), in particular as captured by self-reported disorganized checking (hypothesis 4Aii (*P* < 0.001)) and as captured by individually significant items ‘I sometimes checked again for a predator even though I had just finished checking in all directions’ (*P* = 0.003; all *P* values for individual measures are one-tailed and from regressions correcting for other clinical subscales and demographics) and ‘I avoided checking for the predator because I didn’t want to make a decision about when to hide’ (*P* < 0.001). We also replicated the finding that compulsive checking was related to increased threat avoidance (hypothesis 4B, *P* < 0.001), captured by individually significant items such as post-task self-report ‘I hid for longer than necessary because I was afraid to leave the hiding place’ (*P* = 0.03) and the corresponding behavioral measure of slower time to return from the hiding place (*P* = 0.04) and, in exploratory analyses (Table 3), an increased percentage of total time spent hiding (significant when not correcting for other clinical dimensions). We also replicated compulsive checking being linked to increased arousal (hypothesis 4C, *P* < 0.001), but this was no longer significant correcting for all other clinical traits (Supplementary Table 12, *P* = 0.18), which in parts showed high correlations, thus representing a very strict test. The only individually significant measure was higher excitement ratings throughout the task (*P* = 0.004).

In terms of exploratory findings (Table 3) not included in our original hypotheses, we note very strong (BF > 100) evidence for a reduction of the usually positive correlation between excitement and stress during the task.

While the hypothesis subgroups 4Ai and 4Aiii did not replicate collectively (*P* = 0.35 and *P* = 0.15, respectively), individual measures were nevertheless replicated; that is, a decrease in the total percentage of time spent foraging was associated with compulsive checking (*P* = 0.009), and the last action before hiding was less likely to be a forage action as compulsive checking measures increased (*P* = 0.03).

### Somatic anxiety is linked with self-reported worry

Overall, we replicated hypothesis 5, that somatic anxiety (State–Trait Inventory for Cognitive and Somatic Anxiety (STICSA)^42^) and intolerance to uncertainty (Intolerance of Uncertainty Scale Short Form (IUS-12)^43^) are jointly linked to changes in (self-reported) task performance (*P* = 0.036 for overall hypothesis 5). The sub-hypothesis that somatic anxiety is linked to decreased self-reported vigilance due to worry was replicated (H5B, *P* < 0.001). This was captured by (Supplementary Table 11) ‘I avoided checking for the predator because I didn’t want to make a decision about when to hide’ (*P* = 0.008) and ‘After seeing a predator I felt more nervous than before’ (*P* = 0.004).

The sub-hypothesis of intolerance of uncertainty being linked to changed adaptation of the forage/check balance to the environment or mood was not replicated (H5A, *P* = 0.5), and no individual regressor making up this hypothesis was replicated individually (Supplementary Table 11). This was despite the effects in the discovery sample being so strong that even across both samples together (Table 3), BFs for several measures related to reduced reward sensitivity were above 10.

### Apathy and anhedonia and appetitive behavior

Behavioral apathy (Apathy Motivation Index (AMI), Behavioral subscale^40^) was generally associated with task-related behavior (H6; *P* < 0.001). Consistent with our hypothesis (H6A, *P* = 0.003), behavioral apathy was associated with a reduction in both vigilant and threat-avoidant behavior in favor of foraging (H6Ai, *P* = 0.01) despite increased emotional sensitivity to threat (H6Aii, *P* = 0.03, although no longer significant after correcting for other clinical subscales (Supplementary Table 12), but given the high correlation between clinical subscales, this was a very strict test). Individually significant measures (Supplementary Table 11) included an earlier return from hiding (only significant when not correcting for other subscales, *P* < 0.05) and self-reports, for example, ‘I avoided checking for the predator because I wanted to keep diving for food’ (*P* = 0.045) or ‘After seeing a predator I felt more stressed than before’ (only significant not correcting for other subscales, *P* < 0.05). Exploratory analyses (Table 3) revealed in addition very strong evidence (BF = 687) for reduced excitement during the task and strong evidence for improved checking behavior, as evidenced by a larger tendency to finish a round of checking the screen once started rather than switching back and forth (‘C1 versus F0 pre (check. Start),’ BF = 14), overall improved performance (higher total earnings, BF = 86) and increased percentage of time spent foraging (BF = 11). The relationship between apathy and better task behavior was not mediated or moderated through changes to excitement (all *P* > 0.2 for apathy*excitement interaction terms and all main effects of apathy still significant while excitement effects not significant in regressions of form behavior ≈ apathy*excitement + control variables).

Anhedonia (Snaith–Hamilton Pleasure Scale (SHAPS)^41^) was associated with a reduced feeling of task engagement (H6B, *P* < 0.001; H6Bii, *P* < 0.001). For sub-hypothesis H6Bii, individual items that were replicated included (Supplementary Table 11) a decrease in the post-task self-reports “I tried to find the predator as early as possible” (*P* < 0.05) and ‘I tried to gather as much food as possible right before hiding’ (*P* = 0.01) and a finding of reduced excitement during the task (*P* < 0.001). The surprising finding in the discovery sample, that anhedonia was linked with behaviorally measured increased sensitivity to reward, did not quite replicate (H6Bi, *P* = 0.07; *P* = 0.38 when controlling for other clinical dimensions; Supplementary Table 12), with a single individual behavioral effect—an increased impact of reward on time to return from hiding—replicating (*P* = 0.04). In the exploratory machine-learning analyses that follow, we return to a more detailed examination of this effect.

### Gender is linked with task performance

In addition to psychiatric scores, we confirmed that gender affected task behavior (Table 2 (H7, *P* < 0.001) and Supplementary Table 12 for gender effects controlling for all clinical subscales; Supplementary Table 11 for an extensive list of all individually significant measures). Women performed worse (H7A, *P* < 0.001), including in terms of individual items such as reduced foraging before (*P* < 0.001) and after (*P* < 0.001) the discovery of the predator, as well as increased checking (percentage total (*P* < 0.001) and earlier discovery of the predator (*P* = 0.02)). Despite this, they also exhibited an increased tendency for their avatar to be caught by the predator (virtual death; *P* < 0.001). To understand how increased checking could happen in the presence of increased virtual death, we carried out an exploratory analysis, in which we predicted being caught, including (beyond task factors as for all analyses) the checking rate in the previous and in the next epoch (in the same block). This revealed that checking reduced the risk of subsequently being caught (95% Bayesian CI: [–1.28; –0.76], hierarchical model), while being caught increased subsequent checking (95% Bayesian CI: [0.73; 1.11]). As only few data were available for this analysis (as most participants did not get caught sufficiently often for this analysis), we could not investigate the effect of gender on this.

Women were also more stressed in the task (H7B, *P* = 0.02, although this result did not withstand inclusion of all clinical subscales; Supplementary Table 12), including individual items such as stress ratings during the task (*P* < 0.05, but only significant when not correcting for clinical subscales). Women showed reduced response vigor (H7C, *P* < 0.001) as measured by individual significant items such as reduced presses of the foraging button when it was inactive (*P* < 0.001) and reduced impact of reward on pressing inactive forage or check buttons (*P* < 0.001).

Note that clinical and gender analyses () diverged from the pre-registration in one way. Specifically, pre-registered tests included all other demographic and clinical variables as regressors; results reported here are from models without these additional predictors. For comparison, the original tests are reported in Supplementary Table 12. We note that while conceptually appearing similar, these tests differ in that the tests in Table 2 ask whether relationships detected in the discovery sample replicated, while the tests in Supplementary Table 12 examine whether the task behaviors grouped into specific hypotheses each explain variance in clinical scores beyond the variance explained by other clinical scores (even though these clinical scores may well be correlated; see correlations between clinical scores in Extended Data Fig. 2a). Supplementary Table 12 also shows an alternative way of controlling for correlations between clinical scores by pitting clinical subscales against each other in a regression predicting the clinical scores generated from applying the pre-registered hypothesis models (but trained on orthogonalized behavioral measures that have variability due to the irrelevant correlated clinical subscales and demographics removed).

### Exploratory analyses

In the preceding analyses, a few links between clinical subscales and behaviors (hypotheses) were not robust to inclusion of other clinical subscales. This was potentially due to the presence of correlations between the subscales themselves (Extended Data Fig. 2a). To address this, we performed a factor analysis on the individual items of all questionnaires. This resulted in questionnaire factors that, although mostly recapitulating the original questionnaire subscales, showed lower cross-factor correlations (Extended Data Figs. 2b and 3 and Supplementary Fig. 7a).

To test whether task-based measures were uniquely predictive of these new clinical factor dimensions, we used machine learning with separate training and hold-out (test) samples. We fit regularized Bayesian regression models on the larger replication sample, reserving the smaller discovery sample as a test set. See Fig. 3d for a summary and Extended Data Table 1 for detailed statistics.

We first tested what the best possible out-of-sample prediction could be combining all task-based measures (behavioral measures as in the preceding in addition to single-choice computational measures) and post-task self-reports. For all clinical factors, out-of-sample predictions were significant (Extended Data Table 1b), with greater strengths (*r* between 0.23 and 0.29) than for individual items or a small number of conceptually combined measures (). For some clinical factors (for example, compulsive checking), this appeared to be driven by post-task self-reports (*r* = 0.34 versus *r* = 0.11), while for others (for example, anhedonia), effects were driven by task-based measures (*r* = 0.23 versus *r* = 0.12); and yet for others (for example, cognitive anxiety), strongest predictions were obtained combining both types of measures. In general, predictions appear stronger for the richer set of task-based measures derived from epoch-wise analyses (for example, impact of reward on foraging rate or impact of the task on momentary mood) than for computational measures of a more traditional individual-choice model (predicting, for example, each choice to forage or check before predator discovery). Next, we examined whether task-based and post-task self-report measures explained variance in clinical self-report measures beyond that explained by other clinical measures (Extended Data Table 1a). This was the case for all clinical factors when combining across all types of task-based measures and post-task self-report.

**Extended Data Fig. 4:**
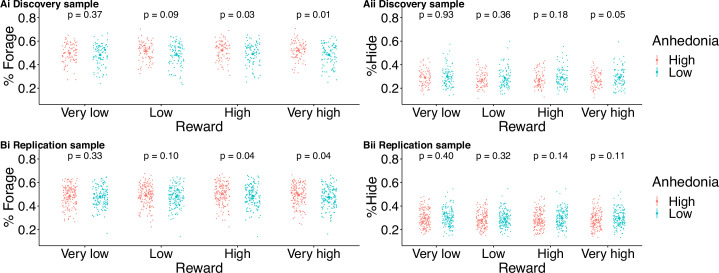
Interaction between anhedonia and reward on % foraging and hiding. A + B) To illustrate the relationship between anhedonia and reward sensitivity (main text, exploratory machine learning results), we split participants by their anhedonia score (SHAPS questionnaire), discovery sample in A and replication sample in B. The 20% of participants with highest anhedonia scores are shown in red (n = 87 discovery sample, n = 160 replication sample) and those with the 20% lowest scores in blue (n = 94 in the discovery sample and n = 149 replication sample). The raw behavior was binned by reward quartiles and the groups were compared for each bin using a two-tailed t-test. This analysis does not correct for other factors impacting the behavior (for example predator speed). For both % foraging and % hiding, the closest to significant are the bins with very high reward, with an absence of a significant difference for low reward values (And if anything – visually a trend for participants with high anhedonia to show more task engagement) suggesting that the effect of anhedonia could not be described as a difficulty motivating behavior at low rewards. Instead, it could be described as increased motivation with high rewards. Individual participants as dots, mean as a larger dot and error bars are the standard error (where the standard error is not visible, it is smaller than the size of the dot representing the mean).

We also used the machine-learning approach to examine whether (as was suggested by some weak evidence in the preceding) anhedonia was linked to a difference in reward sensitivity. We trained predictive models on all relevant behavioral measures capturing sensitivity of the behavior to reward. We found that, in fact, anhedonia can be predicted out of sample (*P* < 0.05, *r* = 0.12), even when correcting for all other clinical dimensions. Individually significant measures included increased impacts of reward on behavior (that is, steeper regression slopes) for percentage of time spent foraging and percentage of time hiding (also distance at hiding). Illustration of these effects by splitting participants by their anhedonia scores (Extended Data Fig. 4) revealed that this might be driven by more anhedonic participants exhibiting particularly high motivation levels when reward was high.

As a final exploratory analysis (Supplementary Fig. 9), we compared the effect sizes for individual difference analyses between the two samples. We found the well-established effect that when significant results were ‘cherry-picked’ on the basis of either sample, the effects tended to be smaller in the other sample. We also found that overall (and true separately for links to gender and to clinical subscales), the larger the initial effects, the larger the effects in the second sample (*r* between 0.47 and 0.88).

## Discussion

In this Article, we examined links between foraging under threat in a novel task and psychiatric traits, gender and mood. We explored relationships between many variables in a discovery sample (*N* = 374), pre-registered a series of hierarchical hypotheses on the basis of significant discovery sample tests and then tested them in an independent replication sample (*N* = 702). We used a transdiagnostic approach that analyzed behavioral patterns beyond conventional diagnostic boundaries.^15^ Our large sample of participants exhibited scores ranging from healthy to clinically significant on psychiatric questionnaires, allowing us to investigate partially correlated behavioral patterns that may feature in many psychiatric disorders. We found robust links between task-related behavior, metacognitive (post-task) self-report measures, mood and clinical traits.

In the task, participants played an avatar that had to forage for reward under threat of predation. To avoid being caught by the predator, they needed to check for predator presence (in a variable number of check directions) and, if appropriate, hide. Features of our task evoked stress and excitement (H1). Specifically, available reward, predator speed and number of check directions increased both excitement and stress ratings (H1A, H1B), suggesting a general arousal effect. In addition, being caught affected stress and excitement in opposite ways (H1C), with participants tending to report greater stress and less excitement after being caught by a predator, suggesting a valence effect.

Emotions have been suggested to be global states that help manage limited cognitive resources by preparing an individual to behave appropriately according to their circumstances. For example, emotions during daily life have been found to change behavior, which in turn then regulated emotions, suggesting a homeostatic process.^39,48–50^ Consistent with this, we found evidence of homeostatic interactions between stress and behavior (H2) even in the context of our well-controlled task. Specifically, stress had a homeostatic relationship with both reward- and threat-oriented behavioral modes (H2A): stress was linked with increased foraging and more efficient checking before predator discovery (H2Ai), and conversely, foraging and efficient checking were linked with reduced stress. We also found that stress increased hiding (that is, threat avoidance (H2B)). In exploratory analyses, we found that, in fact, hiding also showed a homeostatic effect (that is, decreasing stress). Finally, we also looked at whether there were changes in mood simply with time on task, following a recent report^47^ that emphasized decrements in mood (happiness) with time on task. As expected, we found that excitement decreased with time on task. However, we also found that stress decreased in the same way. We excluded the possibility that this was driven by the first blocks of the task showing a ‘settling in to doing an experiment’ effect. Together, this suggests that rather than a decrease in positive valence over time, the results can be better explained by a decrease in arousal over time.

Concerning individual differences in psychiatric traits, we found robust links with compulsive checking (H4), anxiety (H5), apathy (H6), anhedonia (H6) and gender (H7). Importantly, we found that dimensions that often co-occur in poor mental health (anxiety dimension: compulsive checking and anxiety; motivation dimension: apathy and anhedonia) had unique behavioral signatures. We will focus here mainly on a discussion of the most robust results due to space constraints. In particular, where a pre-registered hypothesis was relatively broad, we focus here on the specific aspects that were supported by our replication data.

In the motivation dimension, we measured anhedonia (example (reversed) item: ‘I would be able to enjoy my favorite meal’) and apathy (example (reversed) item for behavioral apathy: ‘I don’t like to laze around’), which co-occur in many disorders (for example, depression). We found distinct behavioral markers associated with each. Anhedonia was linked to a self-reported decrease in the feeling of task engagement. In exploratory analyses of the behavior, anhedonia was linked to an increased modulation of behavior by reward, driven by high behavioral engagement (more foraging, less hiding) when reward was high. This does not fit well with the clinical features of anhedonia (or depression), that is, an inability to enjoy, a lack of motivation to participate in typically rewarding activities^22,51,52^ and difficulty using reward-related information to guide decisions.^24,53,54^ However, related effects have also been previously found in a study on apathy (not measuring anhedonia per se), finding a stronger adjustment of action initiation to reward in people with higher apathy scores.^55^ Turning to apathy, we observed our most surprising, yet robust, transdiagnostic finding. Behavioral apathy was linked to increased foraging versus checking. This was reflected across several task-based measures, including earlier returning to the task from hiding and increased total percentage of time spent foraging, resulting in more overall reward foraged. This occurred in the presence of reduced excitement throughout the task. Participants’ self-reports also reflected the same finding (for example, ‘I avoided checking for the predator because I wanted to keep diving for food’). Exploratory computational modeling revealed apathy to also be linked to increased efficiency of checking (that is, reduced interrupted checking). This is surprising given that depression, apathy and anhedonia have been linked to avoidance of effort required to obtain rewards.^24,25,56^ One interpretation of our results is that apathetic participants were unwilling to switch between behavioral modes, perceiving behavioral switching as effortful. This is reminiscent of a previous finding in a different kind of foraging task where apathy was linked with decision inertia (perseverance in a default behavior rather than making the cognitive effort to switch to an alternative behavior that might ultimately be less physically effortful and time consuming^23^). In the present task, foraging was the ‘default’ action (that is, most time was spent foraging), and checking interrupted this default tendency. The fact that task performance was improved by this approach to the task highlights that the apathetic dimension does not always entail dysfunction. This is consistent with the ecological view that atypical computation styles are not bad by definition, and their utility depends on the individual’s goals and the environmental context.

In the anxiety dimension, we observed distinct findings for somatic anxiety (example item: ‘My heart beats fast’) and compulsive checking (example item: ‘I check things more often than necessary’). We found that the somatic anxiety subscale was linked to reduced self-reported checking behavior due to increased aversion to having to make a decision upon seeing the predator. However, looking across behavioral measures (exploratorily), if anything, we found increased checking (for example, more likely to check as a first action). This may, at first, seem counterintuitive. To understand it, we can consider the literature on attentional biases in anxiety disorders. Anxiety has been linked with two divergent attentional biases using paradigms such as visual search tasks (looking through a list of words for a target) and the dot probe paradigm (measuring reaction time after a target appears in the place cued by an emotional or neutral stimulus):^57,58^ first, hypervigilance and attentional bias toward threat, which can manifest in the absence of actual threat and involve excessive scanning of the environment and greater sensitivity to threat detection, and second, threat avoidance, which might even entail reduced vigilance because a person is actually worried about encountering the threatening item. In fact, participants with more somatic anxiety were more likely to endorse a statement that they avoided checking because they found it aversive (for example, feeling more nervous or indecisiveness about how to act after seeing a predator).

Compulsive checking (as measured by the OCI (revised) ‘checking’ subscale (OCI-RC)) was linked with increased behavioral (for example, returning later from hiding) and self-reported (for example, ‘I hid longer than necessary because I was afraid to leave the hiding place’) threat avoidance. This is in agreement with previous findings on harm avoidance,^59,60^ although it is of note that what previous studies have highlighted is a somewhat different type of behavior from that observed here, with a focus on active harm avoidance (that is, doing something to avoid harm rather than passively avoiding situations; see ref. ^61^ for a discussion) in contrast to the more passive avoidance observed here. Compulsive checking was also linked to increased self-reported disorganized checking (for example, ‘I sometimes checked again for a predator even though I had just finished checking’), although behavioral evidence for this was weak (the pre-registered overall statistical test did not replicate, even though some individual items making up this test did replicate). Self-reports of disorganized checking fit well with the clinical picture of repeated checking.^62^ Studies have highlighted memory distrust^63,64^ or increased decision thresholds^65^ as possible reasons. However, given the lack of behavioral evidence for this, it is possible that this is not in fact a general feature, but dependent on task factors. It is of note that both anxiety and compulsivity were associated with self-reports that are aligned with a ‘folk theory’ of the disease but sometimes not reflected in behavioral measures. This is in line with some findings of reduced metacognitive abilities^66^ although another study^67^ has found reduced metacognitive abilities associated with a compulsive dimension and improved meta-cognition with an anxiety/depression dimension. To test whether the reason is a greater sensitivity of the post-task self-reports or an actual absence of behavior, a future micro-phenomenological study^68^ that probes patients about the subjective specific events in the task could be illuminating. Alternatively, self-reports could be employed more frequently throughout the task. Finally, we found, in exploratory analyses, very strong evidence of a decreased link between stress and excitement throughout the task with increased compulsive checking. There is a sparsity of previous findings on this question to relate this to.

Finally, we followed previous work,^13^ examining gender differences in foraging (H7). Women performed worse overall (they obtained fewer rewards). They tended to favor vigilance over foraging, but despite checking more often and discovering the predator sooner, women were also caught more often. An exploratory analysis revealed that in the sample overall, checking and being caught were related: the more participants got caught, the more they then checked, which in turn then reduced the risk of being caught. Women also showed reduced response vigor, measured as reduced rates of button presses when the buttons were inactive after a previous response. These gender findings are consistent with more successful risk-taking in men.^69^ Men are less cautious and more successful in managing risk–reward trade-offs involving predatory threat^13^ and more tolerant of financial risk.^70^ Alternatively, male participants might have had more experience with this type of task in the form of video games, with previous work finding increased playing times, especially of ego-shooter games, which have been linked to increased performance across various cognitive tasks.^71^ Although of note, the task did not require difficult finger movements (for example, no fast mouse movements required) as there were only three response buttons.

To assess the strength of links between all of our task measures combined (rather than conceptual hypotheses or individual measures) and individual differences, we used machine learning in exploratory analyses. We found that for each clinical trait measured, the task showed significant out-of-sample predictive power. Often, this was even unique, that is, not capturing a general ‘mental health’ dimension, but specific clinical dimensions. For some clinical traits (anxiety, compulsivity, intolerance to uncertainty), the post-task self-reports were the portion of data leading to the strongest predictions; for others (anhedonia), it was the behavior during the task; for still others (behavioral apathy), both types of data were similarly predictive. Overall, these findings indicate the potential for this and similar tasks to be a valuable addition to the diagnostic toolbox. Specifically, this task could be included in clinical studies to provide objective, task-based measures of the clinical dimensions.

In terms of general methodological comments and limitations, first, the study here did not specifically select patients, but rather, clinical traits were captured using self-reports. However, in fact, a high proportion (17%) of participants reported using psychoactive medications, in particular antidepressants. This highlights the usefulness of general population online samples for studying clinically relevant traits. Second, while we find here that our task is robustly linked to mood and clinical traits, it would be important in the future to directly pit it against more standard tasks to assess whether in fact additional predictive power is offered. Third, while combining many different findings in the discovery sample into hierarchical conceptual hypotheses pre-empted problems of multiple comparisons, it meant that the interpretation of significant ‘overall’ hypotheses required detailed inspection of follow-up tests to clarify what drove effects. Fourth, our inability to replicate every single finding, even though the discovery sample had been of substantive size (374 usable datasets) and often producing highly significant results, highlights the advantages of collecting a replication sample and the danger of not doing so. Relatedly, while there was a general strong correlation between the effect sizes observed in the two samples, overall, we also observed the well-known pattern that replication sizes are smaller than in initial discoveries (especially when published in journals with a focus on novelty),^72^ suggesting that sample size calculations should take this into account. Fifth, we surprisingly observed larger correlations between different clinical subscales in the replication than in the discovery sample, making approaches to finding unique links between task measures and clinical traits more difficult, explaining why some findings did not replicate when controlling for other clinical dimensions. This was only in part explained through an accidental change in the age inclusion criterion (inadvertent removal of upper age limit of 40). Sixth, for several clinical dimensions, the strongest links (and out-of-sample predictive power in the machine-learning approach) were found for self-report post-task questions rather than task behavior. This could be due to demand characteristics affecting self-reports and clinical questionnaires similarly. However, more interestingly, the reason could also be that the self-reports allowed us to tap into behaviors that could not be measured with the current task version. For example, self-reports of ‘I avoided checking because I was worried’ could not be behaviorally tested with the sparse mood ratings currently used, but they open further research questions in a task design employing more continuous mood monitoring (for example, extracted from physiological measures).

In conclusion, we demonstrated in a large online study how mood and behavior have a homeostatic interplay. In addition, we showed that clinical traits, even those that superficially appear quite similar, have distinct, replicable patterns of behavior in a task that has potential for future clinical studies.

## Methods

All methods were as pre-registered except a variation of statistical testing that is described in Statistical tests of pre-registered hypotheses.

The experiment was coded in javascript, using jQuery, GreenSock animation platform and noUiSlider animations code. Web applications JATOS^73^ and Pavlovia^74^ and the jsPsych library version 6.0^75^ were used for experiment hosting and data collection.

Analysis was performed in R version 4.3^76^ using Rstan version 2.26.13,^77^ dplyr version 1.0.10,^78^ tidyverse version 1.3.1,^79^ ggpubr versions 0.4.0 and 0.5.0,^80^ compareGroups version 4.5.1,^81^ fuzzyjoin version 0.1.6,^82^ data.Table version 1.14.6,^83^ sjPlot version 2.8.12,^84^ brms version 2.18.0,^85,86^ Stan version 2.26.13,^87^ BayesFactor version 0.9.12-4.4,^88^ Rcpp version 1.0.9,^89–91^ stringr version 1.5.0,^92^ doParallel version 1.0.17,^93^ foreach version 1.5.2,^94^ loo version 2.5.1,^95^ DescTools version 0.99.47,^96^, ggplot2 version 3.4.0,^97^ bayesplot version 1.10.0,^98^ openxlsx version 4.2.5.1,^99^ lubridate version 1.8.0,^100^ jsonlite version 1.8.0,^101^ psych version 2.3.6,^102^ paran version 1.5.2,^103^ mice version 3.16.0^104^ and mifa.^105^

### Participants

We recruited participants (discovery sample: *N* = 419 collected, *N* = 374 included, age range 29.8 ± 6.16 years; replication sample: *N* = 780 collected, *N* = 702 included, age range 35.4 ± 11.0 years) using the online platform Prolific.co (https://www.prolific.co) that fulfilled inclusion criteria (Screening, pre-processing and data quality checks; Supplementary Table 2 has full demographic information). While participants were asked about age, gender and years of education, they were not asked about their ethnicity. This was because such data are regarded as a ‘protected characteristic’ under the UK Equality Act 2010 (UK Public General Acts, Chapter 15; https://www.legislation.gov.uk/ukpga/2010/15). At the time of initiating the study, our understanding was that protected characteristic data should be collected only when there are clear reasons to do so. We had no reason to suspect that the basic psychological processes that are the focus of our study should vary with ethnicity, and so we did not collect ethnicity data. Web applications JATOS^73^ and Pavlovia^74^ and the jsPsych library version 6.0 (ref. ^75^) were used for experiment hosting and data collection. Ethics approval for the study was given by Oxford University Central University Research Ethics Committee (reference numbers: R54722/RE001 and R77387/RE003). All participants provided informed electronic consent before taking part (by ticking a box on the study website; this did not involve a physical signature). Participants could proceed in the study only after giving consent. Data were collected from 11 May 2022 until 7 November 2022. The pre-registration was deposited on 25 November 2022. Data analyses started on 28 November 2022 (other than checks that data were recording correctly on the first ~20 participants). Participants were financially reimbursed for their participation in the study (following Prolific.com guidelines of £6 h^–1^ plus a bonus payment for performance in the task (‘lives’ obtained) of up to £3 h^–1^).

### Sample size

After discovery sample analysis, replication sample size was computed for each clinical hypothesis using the WebPower^106^ package in R. For this, we computed Cohen’s *f*^2^ for each hypothesis (Supplementary Fig. 6). We computed the number of participants required for power between 0.80 and 0.95. This suggested that 580 participants should provide 95% power for all analyses (for *P* < 0.05, one-tailed). We planned to collect 830 participants due to available funds for this project (due to increase in testing time and fees, as well as technical problems with recording some datasets, funds covered 780). Of these, 402 were recruited under ethics approval R77387/RE003, and the rest were recruited under approval R54722/RE001. Our planned replication sample size exceeded the number dictated by power analysis to use the remainder of money set aside for data collection.

### Study outline

Participants first provided informed consent, then completed a demographic questionnaire (age, gender, education, browser), then read the task instructions, then did practice trials, then completed a multiple-choice test (see Supplementary Methods for exact questions asked) to ensure understanding of the instructions, then the main task, then a questionnaire (debrief) about their experience of the task (including a question about problems with the task and otherwise ratings scales) and finally the questionnaires described in the following. If participants did not pass the multiple-choice test, they had to reread the instructions and re-attempt the multiple-choice test (after every attempt, they were shown which question was not answered correctly).

### Task

We designed a gamified online foraging task in which participants freely made a series of choices with the goal of earning as much money as possible. During the task, participants used arrow keys to control an animated fish in an ocean environment where there was rewarding food (later translated to money), threatening predators (leading to large point loss if they ‘caught’ the fish) and a hiding space (where participants could hide from the predators; Fig. 1). Participants could choose freely between three actions: ‘forage’ for food, ‘check’ for predators and ‘hide’ in a safe space. Task instructions are included in the supplementary methods.

#### Forage

The average amount of food available varied randomly (range: 0–90 units; samples were drawn from a normal distribution around the mean with *σ* = 2 s^–1^) by predator epoch (the time starting from either block start or previous predator exit and ending with either the fish being caught or the predator exiting the environment). Participants could always see how much food was available (height of the green bar at the bottom and additionally shown as a number when foraged). If participants ‘foraged’ by pressing the right arrow key, the fish dived down to obtain food, thereby increasing its supply of energy; when an energy bar at the left of the screen was full, the fish gained a ‘life,’ which corresponded to extra payment. The total reward bar carried over between blocks, meaning that even at the end of a block, participants were still incentivized to forage.

#### Check

Predators were hidden from participants’ view unless they ‘checked’ a specific portion of the surrounding area. Predators appeared at the edge of the screen and moved toward the fish’s location at the screen center. When the predator reached the center of the screen, it either caught the fish (causing the participant to lose one life) or, if the fish was in hiding (see the following), it quickly exited the environment. By checking, participants could see whether a predator was in a specific area of the environment and how close it was. To check, participants pressed the up-arrow key. If the current predator was undiscovered, the section being checked advanced clockwise to the next section of the environment at each key press; after the participant discovered the predator’s location, successive up-arrow key presses revealed the predator’s location. There were three types of predator, which differed in speed (10 s, 15 s and 20 s to reach the screen center). Each block (see the following) had only one type of predator, appearing one at a time, and participants were informed which before the block start. There was a random delay (2.0–10.5 s) between the block start or previous predator disappearance and the appearance of the subsequent predator.

#### Hide

Pressing the left arrow key caused the fish to escape to a safe space in a corner of the screen where it could not be caught by the predator. From the cave, the center of the screen was always visible; therefore, the participant could see the predator reach the screen center and then retreat. While hiding, the participant could press the left arrow key again to return the fish to the center.

### Blocks

Each of 20 blocks lasted 90 s and had a different combination of (1) predator speed (slow, medium, fast) and (2) number of segments in which participants could check for predators (range 1–4). With fewer segments, more of the environment was visible during each check action, and therefore fewer checks were required to survey the entire surrounding area. Each participant received one of two schedules (due to a coding error, schedules were distributed to one-third and two-thirds of participants, respectively), each having block and reward conditions randomly generated, with the absolute value of all correlations between task features kept below *r* = 0.3. Within each block, there were several predator epochs (that is, from the beginning of a block or the disappearance of the previous predator until the arrival of the predator in the center).

### Mood ratings

After each block, participants answered two questions about how they perceived the block: (1) ‘How stressful was the last round?’ and (2) ‘How exciting was the last round?’ Participants responded by moving a slider between 0 and 100.

### Timings

Each action involved a time cost: foraging took 1.5 s, checking took 0.5 s, hiding took 0.5 s, and returning from hiding took 2.0 s. Only after this duration were buttons active again (although button presses while the buttons were inactive were recorded). Blocks lasted 90 s, and inter-block time was variable (however long participants took for the mood ratings or any other breaks they wished to take). The delay before appearance of a new predator was random (range: 2.0–10.5 s). Reward magnitude fluctuated at each second.

### Training

Before entering the experimental blocks, participants clicked through task instructions, completed two practice blocks and completed a multiple-choice quiz testing their task understanding (they had to answer correctly to advance).

#### Questionnaires

Before the task, participants were asked for demographic information. Demographics were coded as follows: gender was coded as 1 for male, 2 for female, and 1.5 for ‘other’ (in regression analyses where gender was a covariate of no interest, ‘other’ was coded as 1.5; when predicting gender, ‘other’ was omitted and gender was binary); and education was coded monotonically (options: GCSE or middle school graduation (education up to age 15/16), A-levels or high school diploma, bachelor’s degree, master’s degree, doctorate or similarly advanced qualification).

After the task, participants completed questionnaires to measure psychiatric symptoms and traits. First, they rated how much they identified with statements on the AMI, including both the behavioral subscale (which posed statements such as ‘I enjoy doing things with people I have just met’) and the emotional subscale (included statements such as ‘I feel bad when I hear an acquaintance has an accident or illness’).^40^ Next, they indicated the degree to which each statement was true of them on the STICSA, which included ‘somatic’ items such as ‘My muscles feel weak’ and ‘cognitive’ items such as ‘I think that the worst will happen.’^42^ Third, they indicated their agreement with statements on the SHAPS, which included statements such as ‘I would enjoy my favorite television or radio program.’^41^ Fourth, they indicated how much particular experiences related to daily life checking behaviors (for example, ‘I check things more often than necessary’) bothered them in the last month on the OCI-RC.^44^ Fifth, they responded to statements such as ‘I am better able to accept myself as I am’ on the decentering subscale of the Experiences Questionnaire.^45^ Finally, participants indicated their agreement with statements on the IUS-12,^43^ including its prospective subscale (P-IU; for example, ‘Unforeseen events upset me greatly’) and inhibitory subscale (I-IU; for example, ‘When it’s time to act, uncertainty paralyzes me’).

To screen for data quality, one question from each of the three AMI, SHAPS and OCI-R questionnaires was repeated at the end of the questionnaire session (‘consistency questions’; see the following for exclusion criteria). During analysis, all questionnaire subscales were analyzed separately.

Participants also completed a 29-item debrief questionnaire post-task in which they reported their metacognitive awareness of their task behavior (Supplementary Table 3). This included marking how often each statement was true for them (response options were a seven-point scale from 0/never to 6/very often).

Relationships between questionnaire subscales were visualized using correlation matrices. Given our large sample size, we performed Pearson correlations despite non-normality of questionnaire distributions.^107^ Repeating analyses with Spearman correlations revealed similar results, with correlation coefficients differing by no more than *r* = 0.04.

#### Screening, pre-processing and data quality checks

On the online platform (Prolific), the study was advertised only to participants fitting the age (18+) and language comprehension (English) requirements. To ensure data quality, data were screened for indicators of inattentiveness, poor performance and errors in recording due to technical problems. Screening criteria are described in the following (see Supplementary Fig. 1 and Supplementary Table 1 for amount of data omitted per criterion).

### Pre-screening

Participants were at least 18 years old and were native English speakers. For the replication sample, participants recruited under ethics approval R77387/RE003 were pre-screened on the basis of attentiveness and data quality in two unrelated online tasks as part of a larger study (they had access to the present task only if they performed well in the other tasks). They were also pre-screened using measures of anxiety from the STICSA questionnaire,^42^ measures of apathy from the AMI questionnaire and measures of anhedonia from the Temporal Experience of Pleasure Scale.^108^ Participants were selected such that scores in the final sample were evenly distributed across the lowest, middle and highest thirds of the possible score range for both anxiety and apathy/anhedonia averaged.

### Inattentiveness and poor performance

Participants were excluded if they had too many (>6) epochs in the experiment without any actions, if they checked too rarely (<40 times), if they were caught too often (>9 times), and if they earned fewer than 5 extra lives. To check attentiveness to questionnaires, three questions were repeated, and participants were excluded if the average absolute difference in repeated question scores was larger than one.

In addition, epochs were screened for measures of inattentiveness. Problematic epochs were either excluded from certain behavioral measures or excluded altogether. Epochs were excluded entirely if participants stayed in hiding at the beginning of the epoch for more than 2.2 s or if participants hid more than once. Behavioral measures were computed from epochs depending on the epoch outcome: if participants discovered the predator and successfully hid, epoch data were used to compute all behavioral measures; if participants discovered the predator but the block ended before they could hide, epoch data were used to compute all pre-discovery behavioral measures; if participants did not discover the predator in an incomplete epoch with at least 10 s of data, epoch data were used to compute the ‘rate of inactive button presses’ and ‘time to first action’ measures; if participants did not discover the predator in an incomplete epoch with less than 10 s of data, epoch data were used to compute only time to first action; if participants were caught by the predator, all within-epoch behavioral measures were computed, but the data were not used to compute measures across epochs (for example, time to first action after the predator leaves).

### Technical errors

Participants were excluded if they reported technical problems with the task, including the predator or the reward animations showing incorrectly. In addition, data were screened for unreported technical errors. If errors were detected in a predator epoch, that epoch and all subsequent ones in that block were removed from data analysis. If too many epochs were removed, the participant was excluded. The cut-off for what was considered too many removed epochs was determined on the basis of the replication sample, such that at most 2.5% of participants were removed by a single behavioral measure.

Two indicators of technical errors were used to exclude data. (1) Any actions that happened in an epoch after a predator caught the participant were removed (for example, a participant might have pressed the forage button while on the screen showing they had been caught). (2) After each action, there was an enforced delay before the buttons became active again (1.5 s for forage, 0.5 s for check). Technical problems sometimes meant that this was not respected. We used a difference by more than 100 ms as a cut-off for exclusion.

### Questionnaire quality

Before completing the questionnaires, participants were reminded that this was an important part of the study and that some questions would repeat to check for data quality. At the end of the questionnaires, participants were shown three repeat questions: SHAPS Q4 (‘I would find pleasure in my hobbies and pastimes’), AMI Q13 (‘I feel awful if I say something insensitive’) and OCI-R Q2 (‘I check things more often than necessary’). These questions were selected on the basis of researcher judgements that they were questions participants were likely to have a strong opinion about. In fact, all of these questions showed a strong leftward skew, with the lowest (that is, least psychiatrically symptomatic) answer being the most frequently picked, meaning that—in contrast to questions where most answers are scored in the middle of the range—divergences between the question repeats were more easily detectable. We computed for each participant the average of the absolute difference between their repeat answers for each pair of questions.

#### Statistical tests of pre-registered hypotheses

In summary (Extended Data Fig. 1), to generate hypotheses, we first tested all possible links among collected measures in the discovery sample with regressions linking task measures to mood, clinical scores or gender. Then we grouped the findings in conceptual hierarchical hypotheses. Then we pre-registered the hypotheses (https://osf.io/3gb8n, discovery sample results can be found there as well). Then we tested them in the replication sample.

#### Task-based measures

First, we extracted the behavioral measures (Supplementary Table 5 has a full list). To capture the interplay between emotions, environment, behavior and psychiatric traits, we extracted six types of behavioral measure: (1) impact of task features (for example, speed) on behavior; (2) impact of task features on mood; (3) impact of mood on behavior; (4) impact of behavior on mood; (5) post-task self-reports; (6) behavioral measures pertaining to the overall experiment (non-blockwise). Details of each measurement type follow.

### Impact of task features on behavior

We ran regression analyses as non-hierarchical Bayesian analyses, linking behavioral measures to task features (for all clinical hypotheses, H4–7). A regression model for any behavioral measure *v* that was obtained in each epoch before predator detection was formulated:

$$\begin{array}{l}v={\beta }_{0}+{\beta }_{1}{{\mathrm{reward}}}+{\beta }_{2}{{\mathrm{mo}}}\left({{\mathrm{speed}}}\right)\\\quad+\,{\beta }_{3}{{\mathrm{mo}}}\left({{\mathrm{checkDirections}}}\right)+{\beta }_{4}{{\mathrm{delay}}}\\\quad+\,{\beta }_{5}{{\mathrm{block}}}+{\beta }_{6}{{\mathrm{firstEpoch}}}\end{array}$$

where measures of interest included reward, indicating average epoch reward magnitude; mo(speed), indicating predator speed modeled as a monotonic variable (see ref. ^86^) mo(checkDirections), indicating the number of directions for checking modeled as a monotonic variable; and the intercept. Measures of no interest included delay, indicating the random delay before the predator entered the environment in each epoch; block, corresponding to block number; and firstEpoch, indicating whether the epoch was the first in the block. A regression model for any behavioral measure relating to foraging or checking obtained in each epoch after predator detection was formulated similarly but with the addition of another regressor of no interest corresponding to how much time in seconds remained until predator arrival at the time of predator discovery in that epoch (where a higher value indicates earlier predator detection). In later analyses relating these measures to questionnaire scores, we additionally included the mean of each behavioral measure.

We used different types of link functions available in brms, depending on the distribution of the behavioral measures, including Bernoulli (for binary outcomes, for example, getting caught or not), shifted log normal (for reaction time-like measures that were bounded at zero, for example, time until predator discovery), poisson (for counts, for example, the maximum length of forage sequences before predator discovery) and normal (for all other measures). All regressors were *z*-score normalized, and weak priors were set (normal distribution with mean 0 and standard deviation 3). Predicted variables were not *z* scored to allow the intercept to capture individual differences (for example, main effect in rate of checking). Four chains were run with 4,000 iterations and adapt_delta set to 0.8. Model fit was checked using Rhat < 1.1 and the absence of divergent samples.^77^ If this was not the case, the number of samples and adapt_delta were increased (adapt_delta up to 0.9875 and number of samples up to 64,000). If Rhat or divergence was still not appropriate, the data were excluded from further analyses. Regressions were run only if at least one epoch was available for all conditions of predator speed and directions for checking, if more than 15 epochs were available overall, and if the behavior was not the same in all epochs (as could be the case, for example, for having been caught, which might have never happened). Otherwise, this specific behavioral measure was recorded as missing for this participant and omitted from analyses (complete case analysis).

### Impact of task features on mood

Participants rated mood (stress and excitement) after each 90 s block. We fit regressions separately for each participant predicting mood ratings (for mood hypotheses H1A–C and all clinical hypotheses H4–7). For example, stress ratings given after each block *s* were modeled:

$$\begin{array}{l}s={\beta }_{0}+{\beta }_{1}{{\mathrm{rewardMax}}}+{\beta }_{2}{{\mathrm{rewardMin}}}+{\beta }_{3}{{\mathrm{mo}}}\left({{\mathrm{speed}}}\right)\\\quad+\,{\beta }_{4}{{\mathrm{mo}}}\left({{\mathrm{checkDirections}}}\right)+{\beta }_{5}{{\mathrm{livesGained}}}\\\quad+\,{\beta }_{6}{{\mathrm{block}}}+{\beta }_{7}{{\mathrm{caught}}}\end{array}$$

where rewardMax and rewardMin indicate range of reward in the block, mo(speed) indicates predator speed modeled as a monotonic variable, mo(checkDirections) indicates the number of directions for checking modeled as a monotonic variable, livesGained indicates extra lives gained by foraging, block corresponds to block number, and caught indicates whether the fish was caught by the predator at any point during the block. Note that this is different from the formula reported in the pre-registration document, which was accidentally reported wrongly. The formula here was used in both the discovery and the replication sample. Other mood ratings (for example, post-block excitement) were modeled similarly. All regressors were *z* scored across participants. Settings for brms models were as in the preceding. Because not all participants were caught during the task, for those that were never caught, the regression coefficients for the impact of having been caught on mood were not extracted (due to the use of priors, regressions overall could be estimated even without variability in the regressor for having been caught).

### Impact of mood on behavior

We analyzed the influence of pre-block mood on task behavior (for mood hypotheses H2Ai, H2B and H3A). For this, we predicted the residuals of regressions predicting behavior on the basis of task features (described in the preceding; Fig. 1a). These were averaged within each block to match the number of behavioral measures and mood ratings. We included as predictors mood ratings before the block from which the behavior was taken coded in different ways: (1) including only stress or excitement (for H2B) and (2) including both stress and excitement, that is, measuring the independent contributions to behavior (for H2Ai and H3A and exploratory machine-learning analyses). By using the residuals of the regression of task features to behavior, we ensured that we tested the impact of mood beyond the impact of task features on behavior. For example, the residual *y* of a regression predicting a behavioral measure on the basis of task features was modeled as a dependent variable explained by pre-block stress and excitement:

$$y={\beta }_{0}+{\beta }_{1}{{\mathrm{preblockStress}}}+{\beta }_{2}{{\mathrm{preblockExcitement}}}$$

All predictors were *z*-score normalized across all participants. Settings for the brms fitting procedure were as defined in the preceding.

### Impact of behavior on mood

We examined the impact of behavior on post-block mood (for mood hypotheses H2Aii and H3B) by relating behavior during each block to the mood rating given right after each block. Specifically, we predicted the residuals of regressions predicting mood on the basis of task features described in the preceding (Fig. 1b). As predictor, we included in a separate analysis each behavioral measure (for example, rate of foraging). The analyses were run as separate regressions for each person (settings for brms as in the preceding):

$$r={\beta }_{0}+{\beta }_{1}{m}_{i}$$

where *r* is the residual of a regression predicting mood on the basis of task features and *m*_*i*_ is a behavioral measure such as foraging rate. We also included the mood before the block. Sometimes, we also included the other emotion (pre and post) as predictors. In the results, we note when this was done or not. We pre-registered for which analyses to do this or not to do this and followed this strictly in the replication sample.

Note that in the discovery sample, from which we derived the hypotheses of the confirmation sample, we additionally performed an analysis predicting mood reported not immediately after the block (as done here), but 90 s later (that is, after the next block). Only findings that were significant in both types of analyses were included in the pre-registered hypotheses. This was done to exclude the possibility that the mood reported directly after the block had in fact already changed during the block; in other words, to focus on mood that influenced behavior rather than mood that resulted from behavior. For the replication sample, the analysis of these later moods was used only for the exploratory analyses of whether hiding decreased subsequent stress.

### Post-task self-reports

After the end of the experiment (for all clinical hypotheses), participants completed 29 questions about their behavior in the task (‘debrief’; Supplementary Table 3). These were extracted without further pre-processing and related to participants’ questionnaire answers.

### Behavioral measures pertaining to the overall experiment

Some behavioral measures (for all clinical hypotheses), such as total payment at the end of the experiment, could not be computed in a blockwise manner. Those were extracted without further pre-processing and related to participants’ questionnaire answers.

#### Hypothesis testing

Once each behavioral measure had been extracted, we tested the pre-registered hypotheses in a hierarchical manner: we first tested general hypotheses (for example, compulsive checking is related to task behavior) before then tested the more specific sub-hypotheses if the overall hypothesis was significant (with this hierarchical method, we addressed the problem of multiple comparisons). This was done in very subtly different ways for the mood-related hypotheses (H1–H3) and the individual differences hypotheses (H4–H6). For the mood-related hypotheses, we used the discovery dataset to design a test that specified which regressors should be significant (and in which direction) and then perform a test across their average when analyzing the confirmation sample. By contrast for H4–H6, we designed regression models, trained them on the discovery sample and applied them to the replication sample to generate predictions (for example, predicted anhedonia). These predicted scores were then compared with the actual scores.

### Mood hypothesis testing

We tested whether there were interactions between the task, evoked mood states and task-related behavior. Each hypothesis and sub-hypothesis was then tested using one-sided *t* tests or Wilcoxon tests (choice of test was pre-registered for each hypothesis) with significance determined at *P* < 0.05 one-tailed. Specifically, for hypotheses H1 and H2, Wilcoxon tests were used, and for H3, *t* tests. In some cases, a hypothesis test involved several regression weights (for example, see H1A, which is composed of three regression weights). In this case, the weights were added together before testing, taking into account whether their signs needed to be flipped due to how the variables were coded (based on the discovery sample). The only exception from this approach was H1C, which specified that stress and excitement were affected in opposite ways by having been caught. That statistic was computed as the least significant *P* value of the two individual tests.

### Clinical and demographic hypothesis testing

On the basis of the discovery sample results, we generated clinical hierarchical hypotheses for all subscales (compulsive checking, state and trait anxiety, intolerance to uncertainty, apathy and anhedonia) and gender. A fourth group containing miscellaneous hypotheses (those that we could not group by concept) was also pre-registered for completeness but not included in hierarchical tests. Clinical hypotheses were tested by applying the regression models fit to the discovery sample to the replication sample, generating clinical score predictions for each person:

$$y \approx {\beta }_{0}+{\beta }_{1}{m}_{1}+{\beta }_{2}{m}_{2}+{\beta }_{3}{m}_{3}\ldots$$

where *y* is a clinical or demographic measure and *m*_i_ are task-related measures. These models were fit in brms in refs. ^85–87^ with 2,000 iterations, 4 chains, weak priors (normal(0,1) for intercept and parameters), *z*-scored parameters and family function Gaussian.

Significance of model predictions was assessed with correlations (Pearson’s *r*) for continuous or percentage (%) accuracy for binary predicted variables, out-of-sample *R*^2^, BFs and regressions.

For out-of-sample *R*^2^ (${R}_{{{\mathrm{OOS}}}}^{2}$; see ref. ^109^), model predictions were compared with predictions from a control model (including only the intercept *β*_0_):

$${R}_{{{\mathrm{OOS}}}}^{2}=1-\frac{{{{\mathrm{MSE}}}}_{{\mathrm{m}}}}{{{{\mathrm{MSE}}}}_{{{\mathrm{null}}}}}$$

where MSE_m_ is mean squared error of the model of interest ((true score – predicted score)^2^), and MSE_null_ is the same for the control model. When a hypothesis encompassed several regressions (for example, hypothesis 6B encompasses regressions predicting different clinical subscales), the ${R}_{{{\mathrm{OOS}}}}^{2}$ terms were averaged. ${R}_{{{\mathrm{OOS}}}}^{2}$ significance was assessed with permutation testing (*N* = 10,000 shuffles of the clinical scores; see ref. ^110^). As permutation testing is non-parametric, no assumptions were checked. Significance was defined as one-tailed 95% (that is, null hypothesis was rejected if the true ${R}_{{{\mathrm{OOS}}}}^{2}$ was larger than 95% of ${R}_{{{\mathrm{OOS}}}}^{2}$ derived from predicting the permuted scores). For correlations (Pearson’s *r*), if nested hypotheses involved more than one clinical subscale, we computed the average correlation after Fisher’s *Z* transformation. Correlation significance was determined using permutation testing (*N* = 10,000, significance level defined as one-tailed 95%). Finally, BF was computed for each model to quantify evidence for and against the null model.^88,111^ For models with clinical score as the dependent variable, a one-tailed BF test for linear correlation was conducted with Jeffreys prior associated with the beta distribution^112^ and rscale = 1.

Hypothesis tests linking demographics with task-related behavior in the replication sample were conducted in a similar way to the clinical tests (see the preceding). However, the only dependent variable tested in demographics hypotheses was gender. Participants reporting ‘other’ as gender were omitted, and the variable was treated as binary. Regression models were fit with family function Bernoulli.

Out-of-sample *R*^2^ (${R}_{{{\mathrm{OOS}}}}^{2}$) for models predicting gender:

$${R}_{{{\mathrm{OO}}S}}^{2}=1-\frac{{{{\mathrm{LL}}}}_{{\mathrm{m}}}}{{{{\mathrm{LL}}}}_{{{\mathrm{null}}}}}$$

where LL_m_ is the log-likelihood of the model of interest and LL_null_ is the same for the control model. To measure goodness of the prediction in these models, we compared predicted and true clinical scores by computing accuracy (percentage correct classification). Significance for ${R}_{{{\mathrm{OOS}}}}^{2}$ and accuracy were assessed with permutation testing as in the clinical models described in the preceding. BF was computed using a one-tailed BF test of single proportions on the number of correct classifications made by each model.^88^

As an additional control analysis, we built regression models similar to the correlations between predicted and actual clinical scores in the replication sample, but additionally including all clinical measures and gender as predictors of predicted clinical scores (obtained from the models trained on the discovery sample). This allowed assessing whether the links between behavior (and thus predicted clinical scores) and actual clinical scores were robust to controlling for other clinical measures. Note that linear regression models, especially for large sample sizes (>10 observations per regressor) as used here, are robust to violations of assumptions.^113^ We therefore did not test assumptions.

### Follow-up tests on individual behavioral measures

To identify links between clinical scores/gender and the task in the discovery sample, we used linear non-Bayesian regression analyses that allowed parsing out unique contributions of clinical subscales by controlling for demographics and clinical variables of no interest. For example, for analyses where the main variable of interest was any of the anxiety subscales, we controlled for all apathy, anhedonia and compulsivity subscales and demographical measurements (age, gender, education):

$$\begin{array}{l}{m}_{i} \sim {\beta }_{0}+{\beta }_{1}{{\mathrm{traitAnxiety}}}+{\beta }_{2}{{\mathrm{apathy}}}\left({{\mathrm{emotionatl}}}\right)\\\quad\;+\,{\beta }_{3}{{\mathrm{apathy}}}\left({{\mathrm{behavioral}}}\right)+{\beta }_{4}{{\mathrm{anhedonia}}}\\\quad\;+\,{\beta }_{5}{{\mathrm{compulsive}}\; {\mathrm{checking}}}+{\beta }_{6}{{\mathrm{gender}}}+{\beta }_{7}{{\mathrm{education}}}+{\beta }_{8}{{\mathrm{age}}}\end{array}$$

where *m*_*i*_ is a task-based measure and ${\beta }_{1}{{\mathrm{traitAnxiety}}}$ is the clinical variable of interest. For analyses where the main interest was any apathy or anhedonia subscales, we controlled for all anxiety and compulsivity subscales (for IUS-12, only the P-IU subscale was used as a control because it highly correlated with the I-IU anxiety subscale) and demographics. For analyses focused on OCI-RC score, we controlled for all apathy, anhedonia and anxiety subscales, as well as demographics. For analyses focused on demographics, we controlled for all anxiety, apathy, anhedonia and compulsivity subscales, as well as all other demographics. When conducting regressions involving debrief questions 1–25, we additionally controlled for the sum of the participant’s response to debrief questions 27–29 (‘How much time did you spend performing each of these actions?’ Q27: ‘Eating food,’ Q28: ‘Checking for a predator,’ Q29: ‘Hiding from a predator’); this was to account for an individual’s tendency to use the right- or left-hand side of the scale more often. Significant results from these regressions were grouped by concept, arranged hierarchically from general to specific and pre-registered as hypotheses. In the replication sample, we repeated these analyses as a post hoc test for each measure making up the hierarchical analyses (Supplementary Table 11). In exploratory analyses, we tested for links to questionnaire subscales or questionnaire dimensions for all behaviors across the two samples, computing BFs comparing regression models with and without the clinical subscale/dimension of interest, separately correcting for all other clinical dimensions or correcting only for demographics. Results were then selected on the basis of BF being at least larger than 3 (standard cut-off for ‘moderate’ evidence).

#### Summary of divergence from pre-registration

Note that clinical and gender analyses () diverged from the pre-registration in one way. Specifically, pre-registered tests included all other demographic and clinical variables as regressors; results reported here are from models without these additional predictors. For comparison, the original tests are reported in Supplementary Table 12. We note that while conceptually appearing similar, these tests differ in that the tests in Table 2 ask whether relationships detected in the discovery sample replicated, while the tests in Supplementary Table 12 test whether the task behaviors grouped into specific hypotheses each explain variance in clinical scores beyond the variance explained by other clinical scores (see correlations between clinical scores in Extended Data Fig. 2). In retrospect, and shown as exploratory analyses in the following, a better way to test whether the task overall (rather than just small selections of specific behaviors) explains variance beyond other questionnaires uses data mining (rather than conceptual hypotheses). Supplementary Table 12 also shows an alternative way of controlling for different questionnaire scales using regression residuals. This correction replicates the findings for most hypotheses. An additional minor change is that the main text now reports the results in terms of correlation between predicted and actual individual difference scores. *R*^2^ are shown in Supplementary Table 12 and lead to the same statistical conclusions.

In addition, while preparing revisions, we noted a bug in the code (after improved warning messages due to dplyr software update), which affected <2% of participants. We corrected this. After this correction, all analyses were rerun and the following changes observed: the impact of stress on whether any checks occurred post-discovery were no longer significant in the discovery sample; this measure was nevertheless included in all pre-registered analyses.

#### Exploratory analyses

In addition to the pre-registered analyses, we performed factor analysis on task and questionnaire data, machine-learning, single-choice decision-making computational models.

##### Factor analysis

On the basis of reviewer advice, we sought to test whether the data can be simplified using factor analysis as a method of dimensionality reduction, without loss of information. For this, we performed two separate factor analyses on the questionnaires and the behavioral measures of the replication sample.

First, for the task-based data, the data included the post-task debrief self-reports, the impact of task on behavior, the impact of mood on behavior, the impact of behavior on mood and the regression weights from the single choices decision-making models (see the following). Data were further pre-selected according to all measures that had been included in the pre-registration; only measures that were significant on the group level (for regression weights, for example, amount of reward on foraging rate); measures having less than 20% missing data (for example, if a participant never got caught, the impact of reward on getting caught could not be estimated); measures showing correlations (Pearson’s *r*) below 0.9 between measures (for this, measures were iteratively included, checking against already included measures). This meant a total of 207 measures were available.

When deciding how to run the factor analysis, we considered several options: first, performing factor analysis separately on each of these types of measures (for example, all self-report measures separately) then combining the factors; second, performing factor analyses on all data combined; third, performing factor analysis on all types of data combined, but using only a pre-selection of items, based on factor solutions of method one (that is, separately for each measure). To identify the best method, while not affecting statistical validity, we used only the discovery sample to decide on the method and limited statistical tests that the chosen model was then applied to the replication sample. For exploratory factor analyses (discovery sample), we used the psych and the paran packages in R. We determined the numbers of factors using a version of parallel analysis,^114^ proposed by ref. ^115^, which has the advantage that fewer factors are proposed (cut-off is factor significance compared with 99% percentile, based on 5,000 iterations). In the creation of questionnaires based on factor analysis^116^, a common method is to include items only if they show a clear loading onto a factor (loading > 0.4) and no significant cross-loading (that is, no other loadings > 0.4). In the cross-validation procedure, we compared these methods (‘with item pruning’, ‘without item pruning’). Specifically, for the item pruning, we checked after fitting the factor analysis (generalized weighted least squares, oblimin rotation, that is, not enforcing orthogonal factors) the loadings and removed items with low loadings or cross loadings. Then, if items were removed, parallel analysis and factor analysis were repeated. Data were not *z* scored (as factor analysis was run on the covariance matrix), and missing data were replaced using multiple imputation for factor analysis (mifa and mice package); clinical and demographic data were not included during the imputation. We used threefold cross-validation of the discovery sample to check which factor analysis method was best. Specifically, for each of ten questionnaire subscales (or gender), we trained a model on two-thirds of the data, including factor scores from the different approaches. All data were *z*-score normalized. We then computed predictions for the test data. Putting all three sets of test data together, we then computed correlations (or accuracy for gender) for predicted relative to real scores. In the discovery sample, we then applied the best method, which was performing a single factor analysis across all items on items pre-selected on the basis of pruning of factor analyses on different types of data, to assess goodness of fit using confirmatory factor analysis with the lavaan package. We quantified goodness of fit using standard criteria (comparative fit index and root mean square error).

For the questionnaire data, applying the preceding procedure (separate factor analysis first within each questionnaire) removed only very few (two) items. Therefore, we instead directly performed factor analysis on all raw items across all questionnaires combined, again iteratively removing items and recomputing the appropriate number of factors until each item had a single loading above 0.4.

In all cases, factor scores were extracted using the Bartlett method,^117^ ensuring that correlation structures between the raw questionnaire subscales were reflected in the correlations among the factor scores (Supplementary Fig. 7). This was not the case with the default method in the psych package (Thurstone^117^).

To understand whether the behavioral factors explained individual differences in clinical scores better than (or at least as well as) the rawer behavioral measures, we included them in the following exploratory machine-learning analyses. To illustrate the findings, we also (see machine learning below) extracted the links between task factors and questionnaire factors that were most robust across discovery and replication sample.

##### Single-choice regressions

In addition to the pre-registered aggregate behavioral measures, the exploratory analyses included parameters estimated from individual-choice regression models, separately for responses before and after the predator was discovered. For button presses occurring before the predator had been discovered (‘pre-discovery phase’), we included only choices that were check or forage:

$$\begin{array}{l}{{\mathrm{Choice}}}\sim{{\mathrm{Reward}}}+{{\mathrm{time}}\;{\mathrm{pressure}}}\\\qquad\quad\;+\,{\mathrm{ch}}{{\mathrm{eck}}\;{\mathrm{sequence}}\;{\mathrm{started}}}+{{\mathrm{predator}}\;{\mathrm{speed}}}\\\qquad\quad\;+\,{{\#}}{{{{\mathrm{comes}}}}}+{{\mathrm{env}}\;{\mathrm{index}}}+{{\mathrm{first}}\;{\mathrm{Epoch}}}{\rm{Yes}}/{\rm{No}}\end{array}$$

Reward magnitude is the reward available (always visible on-screen*)*. Time pressure, a measure of threat, is how close an unseen predator might be:

$${\rm{Time}}\; {\rm{pressure}}:\,f(t)=\left(\frac{1}{\min {{\mathrm{Delay}}}+{{{\mathrm{speed}}}}_{{\mathrm{b}}}}\right)\times (t-{{{\mathrm{lastFullCheck}}}}_{t})$$

where minDelay is the minimum possible delay for all predators in this task (2.5 s), speed_b_ is the number of seconds that the predator type in block b takes to reach the center of the screen, and lastFullCheck_*t*_ indicates, for timepoint *t*, the timepoint at which the participant most recently completed checking all areas of the environment (that is, when they could be certain that no predator was present).

Check sequence started indicates whether participants had begun to check for the predator (that is, variable was set to 1 once participants had started and again set to 0 when they checked the last area on the screen); participants could not check the same area repeatedly because when pressing the check button, it automatically selected the next area clockwise on the screen. Predator speed (coded as a monotonic factor across the three predator speeds) is the speed of the predator. #Cones indicates the number of areas that can be checked, also coded as monotonic factors. Env index is the index of the environment in the experiment. First Epoch Yes/No indicates whether the current choice is in the first epoch of an experiment (that is, where participants automatically started in the middle of the screen, rather than themselves having to first return there from hiding).

For button presses occurring after the predator had been discovered (‘post-discovery phase’), we did two separate binary regressions, first coding choice as whether to hide or not.

$$\begin{array}{l}{{\mathrm{Choice}}}\sim{\rm{Reward}}+{\rm{Position}}\;{\mathrm{uncertainty}}+{\rm{proximity}}+{\rm{predator}}\;{\mathrm{speed}}\\\qquad\quad+\,\#{\rm{cones}}+{\rm{envIndex}}+{\rm{first}}\;{\rm{Epoch}}\;{\rm{Yes}}/{\rm{No}}\end{array}$$

Position uncertainty indicates how long it had been since the participant had last seen the predator relative to the predator’s speed:

$$f(t)=\frac{t-{{{\mathrm{lastCheck}}}}_{t}}{{{{\mathrm{speed}}}}_{{\mathrm{b}}}}$$

where lastCheck_*t*_ indicates, for timepoint *t*, the timepoint when the participant last saw the predator, and speed_b_ is the speed of the current predator type at block b.

Proximity captures the amount of time until the predator would arrive,:

$${\rm{Proximity}}:f(t)=t-\left({{{\mathrm{delay}}}}_{{\mathrm{e}}}+{{{\mathrm{speed}}}}_{{\mathrm{b}}}\right)$$

where delay_e_ is the scheduled delay for the predator in epoch e and speed_b_ is the speed of the current predator type at block b.

Second, we analyzed choices that were not hide choices as to whether they were forage or check. We included the same regressors as in the analyses of hide versus not hide.

All regressions predicted a binary choice. Regressors were *z*-score normalized, and weak priors were set (normal distribution with mean 0 and standard deviation 3). All analyses of choice data were computed as non-hierarchical Bayesian regression models using the package brms.^85,86^ Four chains were run with 8,000 iterations and adapt_delta set to 0.9. Model fit was checked using Rhat < 1.1 and the absence of divergent samples. Model fits that did not meet these criteria were rerun with increased samples and adapt_delta.

To validate the models, we coded generative models that ‘played’ the task; that is, they used the same regression equations as described in the preceding to make choices throughout each block of the task. Fitting these models directly to participants’ behavior turned out to be difficult due to the very slow evaluation times of a single iteration of a parameter combination, compared with the regression models described in the preceding. Therefore, we instead simulated with the generative models, using parameters derived from real participants’ ranges. A total of 200 sets of parameters were drawn randomly from the parameters estimated on the replication sample participants without respecting correlations between parameters (that is, for each parameter, a random value was drawn, rather than a random participant with all of their parameters). To check parameter recovery, behavior was then fit with the logistic regression models, using the same approach as for the real data. To illustrate how well our models captured real participants’ data, we also performed a simulation using the exact same parameter combinations as real participants and then computed aggregate measures.

##### Machine learning

We used a machine-learning approach, regularized Bayesian regression with predictor selection,^118^ to explore whether task behavior—combined across all measures obtained from the task, rather than grouped into conceptual hypotheses—could predict clinical scores/demographics out of sample. To fit the best possible model, models here were trained on the larger (replication) sample. The discovery sample was used as an independent hold-out dataset, in which predictions of the trained models (for example, predicted behavioral apathy scores) were related to actual scores using Pearson’s *r* correlations (for all clinical scores) or percentage accuracy (for gender).

The data included aggregate measures (as described in the preceding), that is, mood as affected by task, behavior as affected by task (reward, predator speed, number of areas to check and index of the block in the task, that is, time), behavior affected by mood or mood affecting behavior; parameters from the single-choice computational models (see the preceding); and post-task self-reports. This resulted in 424 variables. Of these, we further selected only variables that (where appropriate) were significant on the group level (for example, impact of reward on rate of foraging) and had less than 20% of data missing. This resulted in 257 variables. Across all participants, not including questionnaire scores or gender, missing values were then imputed using the mice package. All predictors were *z* scored. Details of fitting are given in the following.

For this method, we built several types of models. (1) To test our overall ability to predict clinical subscales or gender, we tested models including all behavioral measures (including post-task self-reports) as predictors. (2) To measure the unique predictive power of various task elements, we tested models with various non-clinical and non-demographics measures as predictors: (a) task behavior, excluding mood or its interactions, computed as ‘aggregate measures’ (see the preceding), (b) aggregate task behavior interacting with mood, (c) task behavior as captured by decision-making model of single choices, (d) post-task self-reports, as well as various combinations of these types of data together.

To summarize the method, a regularized model was fit, and the most predictive features were selected.

#### Modeling details

We used brms and Stan together to fit a regularized Bayesian regression. This was implemented with a horseshoe prior on all regressors, with expected significant parameter ratio set to 20%^119^ and a normal(0, 1) prior for the intercept. The expected parameter ratio was not further tuned (using, for example, cross-validation, which could have improved already good predictions even more by reducing some over-fitting risks). Models were fit to the replication data due to the larger sample size. The discovery data were used as an independent hold-out dataset. The variable to be predicted was a single clinical/demographic feature (for example, compulsive checking, a questionnaire measure). Other brms settings were 4,000 iterations, 4 chains, parameters to reduce divergence: target average acceptance probability (adapt delta) = 0.99, max tree depth = 12. Models were checked for fit (Rhat < 1.1), while we note that a very low number of divergences persisted (<20), which is expected given the large number of regressors. In the replication sample, fit of this model was compared with a null model (containing intercept only) using efficient approximate leave-one-out cross-validation (loo; ref. ^120^). Moment matching was used when Pareto *k* diagnostic values were high (*k* > 0.7).

To understand which behavioral measures drove the machine-learning performance, we first wanted to select the most predictive regressors using the projpred package.^118,121^ Predictor selection was necessary because, while a Bayesian regularized model might show an overall better fit to the data than a null model, all of the individual predictors might not be significant. However, visual inspection of criteria for feature selection was sometimes indicative of problems. We therefore used a different approach, that is, extracting, for each clinical dimension or gender, the task-based/self-report measures that showed BF > 3 (that is, at least moderate evidence of an effect).

### Reporting summary

Further information on research design is available in the Nature Portfolio Reporting Summary linked to this article.

## Supplementary information

Supplementary Information Supplementary Figs. 1–9, Tables 1–12 and Methods (task instructions, multiple-choice quiz).

Reporting Summary

Supplementary Video 1 Video of task

## Figures and Tables

**Extended Data Table 1:**
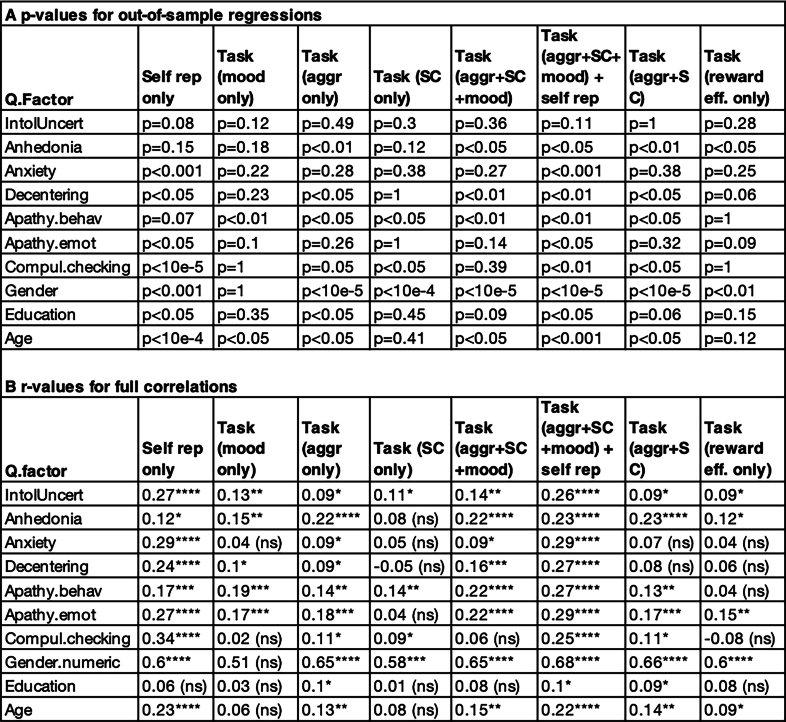
Machine learning, detailed results

Results of applying the regularized regressions trained on the replication sample to the discovery sample. A) Predicting the predicted clinical factor based on all measured clinical factors and demographics (age, gender, education [labeled as ‘education.numeric’ as categories were treated as linear to allow fitting linear models]) in linear regressions – one-tailed p-values (as only positive relationship between predicted and actual were considered, p = 1 for negative regression coefficients); no adjustments for multiple comparisons. B) Same as A, but full correlations (Pearson’s r) or % accuracy (for gender). The measures included were (combinations of): post-task self-reports (‘self rep’), aggregate behavioral measures related to only the task features (‘aggr’) or also to (‘mood’) or obtained from the single-choice regression models (‘SC’). Throughout all analyses (pre-registered or exploratory), we controlled for gender, age and education. While not of main interest for the study, we also tested the predictive power of our task for age and education here. Age was significantly predicted based on all types of task measures, other than the single-choice models, even though the variance explained (out-of-sample R^2^) was negative. This is potentially explained by the fact that in the discovery sample in which significance was assessed, the variance in age was small (18 to 40). When looking at which measures individually related significantly to age, controlling for clinical factors and the other demographics (measured here selected as worth mentioning if the Bayes Factor was above 3), we found that older participants showed worse performance (lower total earning), and, at a behavioral level, this was explained by reduced actions: reduced checking, reduced foraging, earlier and longer hiding, and reduced inactive button presses. In older participants, behavior, (for example % of time spent foraging or checking, rate of foraging, time to the first check in an epoch) was less modulated by the reward or threat. Education level was also predictable from the task, with the only individually significant behavioral measures being an increase in modulation of the % time spent foraging and whether any foraging occurred after the predator discovery by the number of directions to be checked.

## Data Availability

Data available via OSF (10.17605/OSF.IO/NTB5E).

## Code availability

Code available via OSF (10.17605/OSF.IO/NTB5E).
