# Supplementary material for: Emotions and individual differences shape human foraging under threat
Source: Nat Ment Health. 2025 Mar 12;3(4):444–65. doi: 10.1038/s44220-025-00393-8 (PMC11978516; doi:10.1038/s44220-025-00393-8)
Supplement: Supplementary file 1 — Supplementary Figs. 1–9, Tables 1–12 and Methods (task instructions, multiple-choice quiz). [file 44220_2025_393_MOESM1_ESM.pdf]

nature mental health

Supplementary information https://doi.org/10.1038/s44220-025-00393-8

# Emotions and individual differences shape human foraging under threat

In the format provided by the authors and unedited

# Table of Contents

*Supplementary Figures* ........ *2*

**Figure S1. Data quality checks:** ........ 2

**Figure S2. Relationship questionnaire performance and clinical scores.** ........ 4

**Figure S3. Individual participants (related to figure *2A*)** ........ 6

**Figure S4. Participants perform better over time.** ........ 7

**Figure S5. Histogram of selected behavioural measures comparing real and simulated data (n=702, replication sample).** ........ 8

**Figure S6. Power calculation.** ........ 9

**Figure S7. Correlations among questionnaire factor scores and subscales (related to extended data figure 2+3).** ........ 11

**Figure S8.** ........ 12

**Figure S9. Correlation between effect sizes of the two samples.** ........ 14

*SUPPLEMENTARY TABLES* ........ *15*

**Table S1. Participant exclusion criteria** ........ 15

**Table S2. Replication and Discovery Sample Demographics and Clinical Scores.** ........ 16

**Table S3. Psychoactive Medication Reported in Replication Sample** ........ 17

**Table S4. Debrief Questionnaire Items.** ........ 18

**Table S5. Explanation of all behavioural measures** ........ 19

**Table S6. Single-Choice Regression Analyses: Group-Level Results.** ........ 21

**Table S7. Parameter recovery.** ........ 22

**Table S8. Impact of task features on behaviour (related to figure 2B)** ........ 24

**Table S9. List of predictors included in mood-related hypotheses (related to table 1).** ........ 26

**Table S10. Alternative statistical procedures for mood hypotheses** ........ 27

**Table S11. List of predictors included in individual difference (clinical, age) hypothesis (related to table 2).** ........ 29

**Table S12. Additional Statistics Clinical and Demographic Hypotheses (Related to Table 2)** ........ 30

*Supplementary methods* ........ *32*

**[1] Task instructions** ........ 32

**[2] Multiple choice quiz** ........ 34

*References* ........ *35*

**Supplementary Figures**

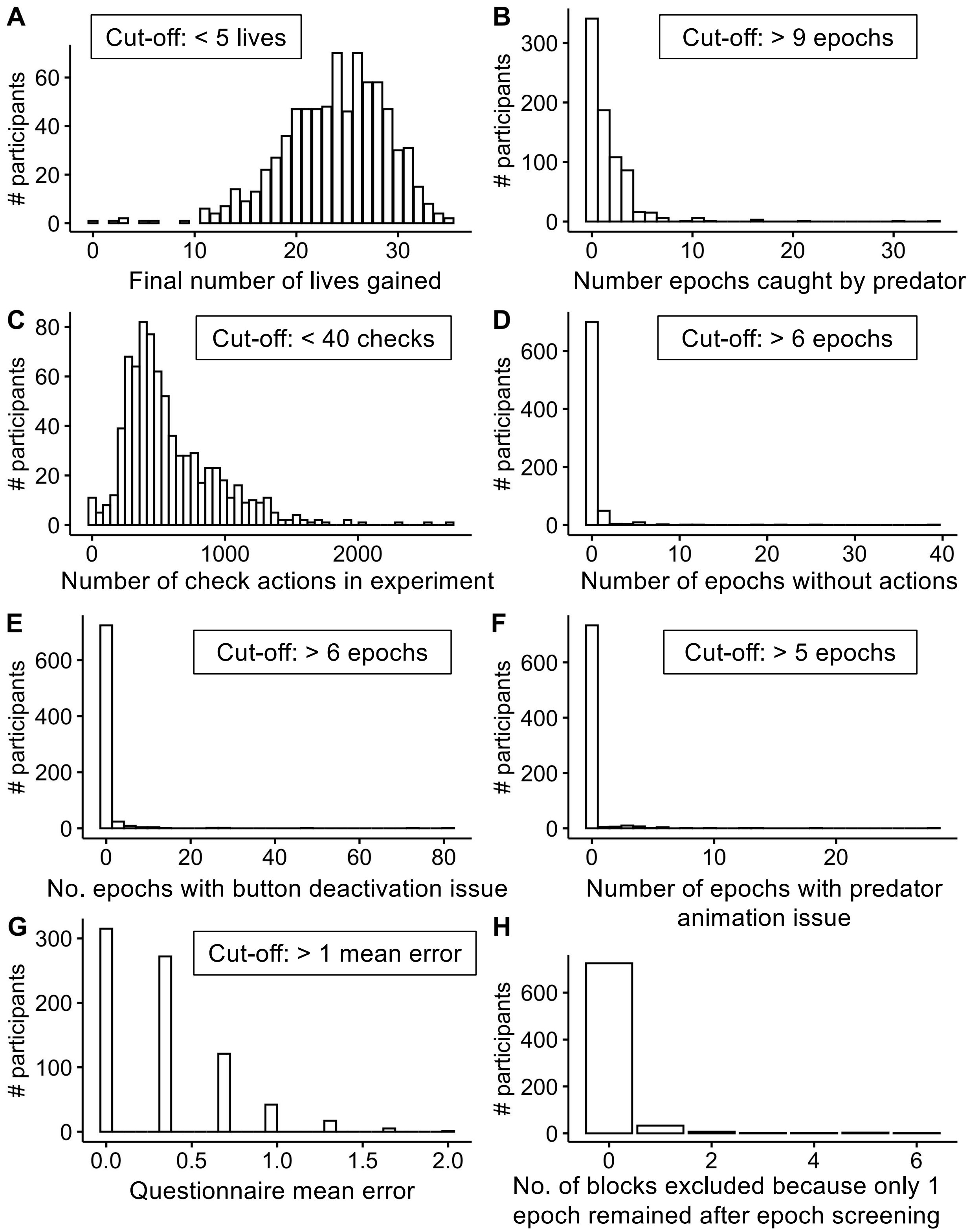


**Figure S1. Data quality checks:**

Histograms showing the distribution of values on exclusion criteria in the replication sample for different exclusion criteria. A) Total number of extra lives gained across participants, with a cut-off of <5 lives (excluded: 4 participants – note that participants might fail more than one data quality check). B) Total number of epochs in which each participant was caught by a predator, with a cut-off of >9 epochs (excluded: 13). C) Total number of check actions in experiment across participants, with a cut-off of <40 checks (excluded: 13). D) Total number of

epochs without actions across participants, with a cut-off of >6 epochs (excluded: 8). E) Total number of epochs in which participants experienced a button deactivation issue, with a cut-off of >6 epochs (excluded: 16). F) Total number of epochs in which participants experienced a predator animation issue, with cut-off of >5 epochs (excluded: 0). All task-behaviour checks together excluded 50 (6.5%) of participants. G) Mean error across attention check questions (i.e. repeated questions) for all participants, with cut-off at >1 mean error (excluded: 23, i.e. 3%). H) Number of blocks excluded because after removing epochs with problems, only one epoch remained in the block. Cut-offs shown in figure legends indicate the x-axis values that were used to omit participants for low-quality data. Cut-off thresholds were chosen based on the discovery sample (chosen so that a single behavioural measure omitted at most 2.5% of participants). Combining these criteria excluded 71 (9%) of participants. n=780.

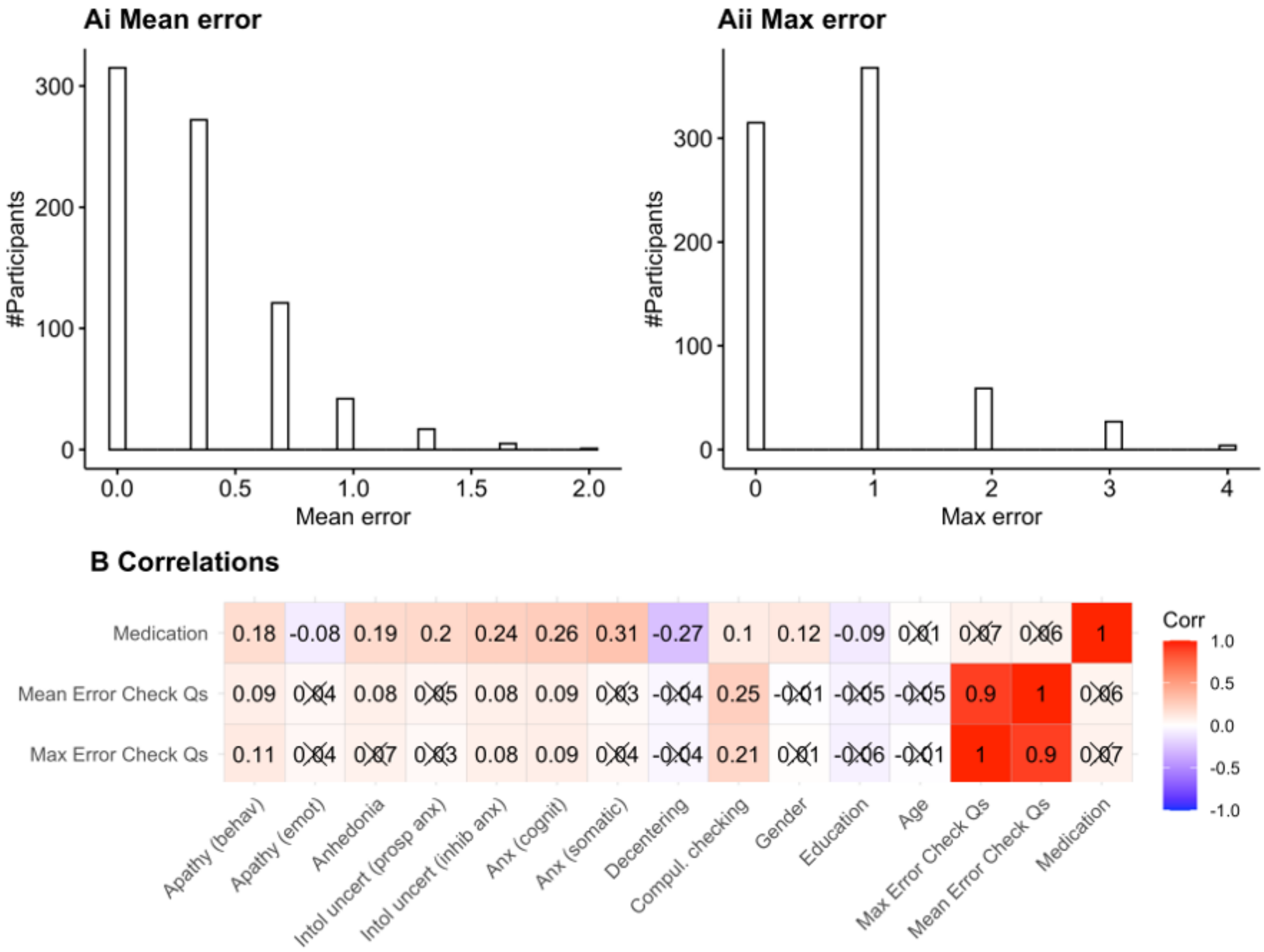

**Figure S2. Relationship questionnaire performance and clinical scores.**

A) In the pre-registration, we decided to exclude participants based on a mean check question error above 1 (specifically, mean of the error across the three check questions). An alternative exclusion criterion could have been based on the maximum error (i.e. the maximum error across any of the three check questions). We show here the histogram of participants scored according to these different criteria (mean error, Ai), max error (Aii). B) Correlations between the mean and max errors, clinical subscales, demographics and medication (yes/no). Due to correlations between clinical subscales and the errors, but no correlations with medication status (though we did not further split this by type of medication), we conducted control analyses (table S12) repeating our key results excluding participants based on any errors in the questionnaire check questions. We also note the positive relationship between medication status and most clinical subscales, giving further support for the validity of the subscales as measuring clinical traits of real-life relevance (i.e. requiring doctor visits and medications). N=702. 'X' indicates non-significant correlations in B, based on two-tailed correlations with Pearson's r, $p<0.05$, no corrections for multiple comparisons.

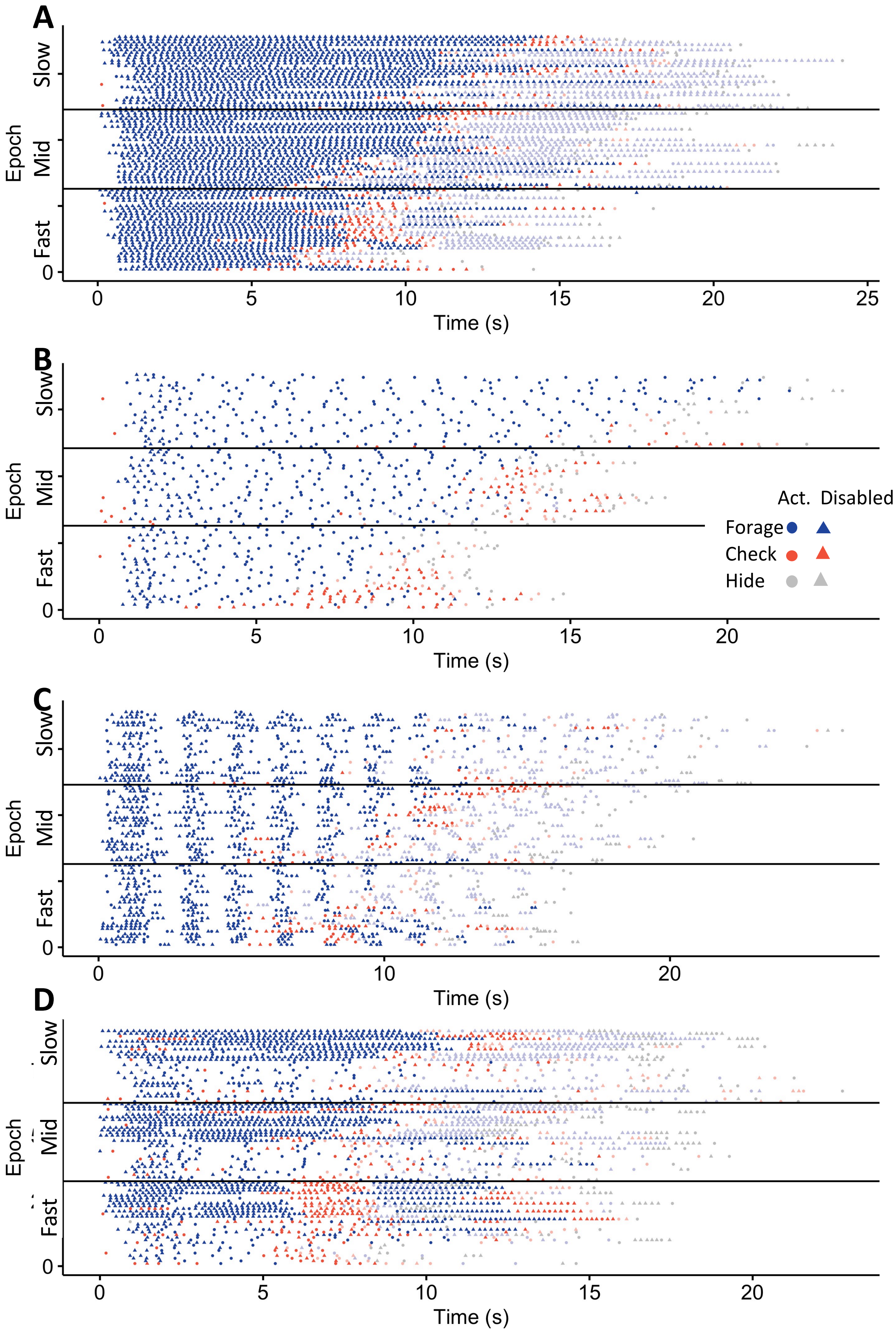
A
B
C
D
Epoch
Slow
Mid
Fast
Time (s)
Act. Disabled
Forage
Check
Hide

**Figure S3. Individual participants (related to figure *2A*).**
Data from four example participants showing behaviour (forage [blue], check [red], hide [grey]), when buttons are active again after fixed delay of 0.5s for check actions and 1.5s for forage actions (round) or still disabled (triangle). Lighter shades show forage and check actions after the predator has been discovered. Behaviour is shown throughout the predator epochs (time in epochs on x-axis) across all epochs of the task (y-axis, epochs sorted for display only by speed of predator (slow, mid, fast). Participants showed a lot of variability in their task behaviour: A) Participant foraging mostly with many button presses (also when buttons inactive) and only checking very late, if at all. B) Participant also only checking very late, but not showing many button presses when buttons disabled. C) Participant showing very regular patterns of forage actions across epochs. D) Participant showing large differences in behaviour early in the task (lower part of each predator speed) compared to later (upper part of each predator speed).

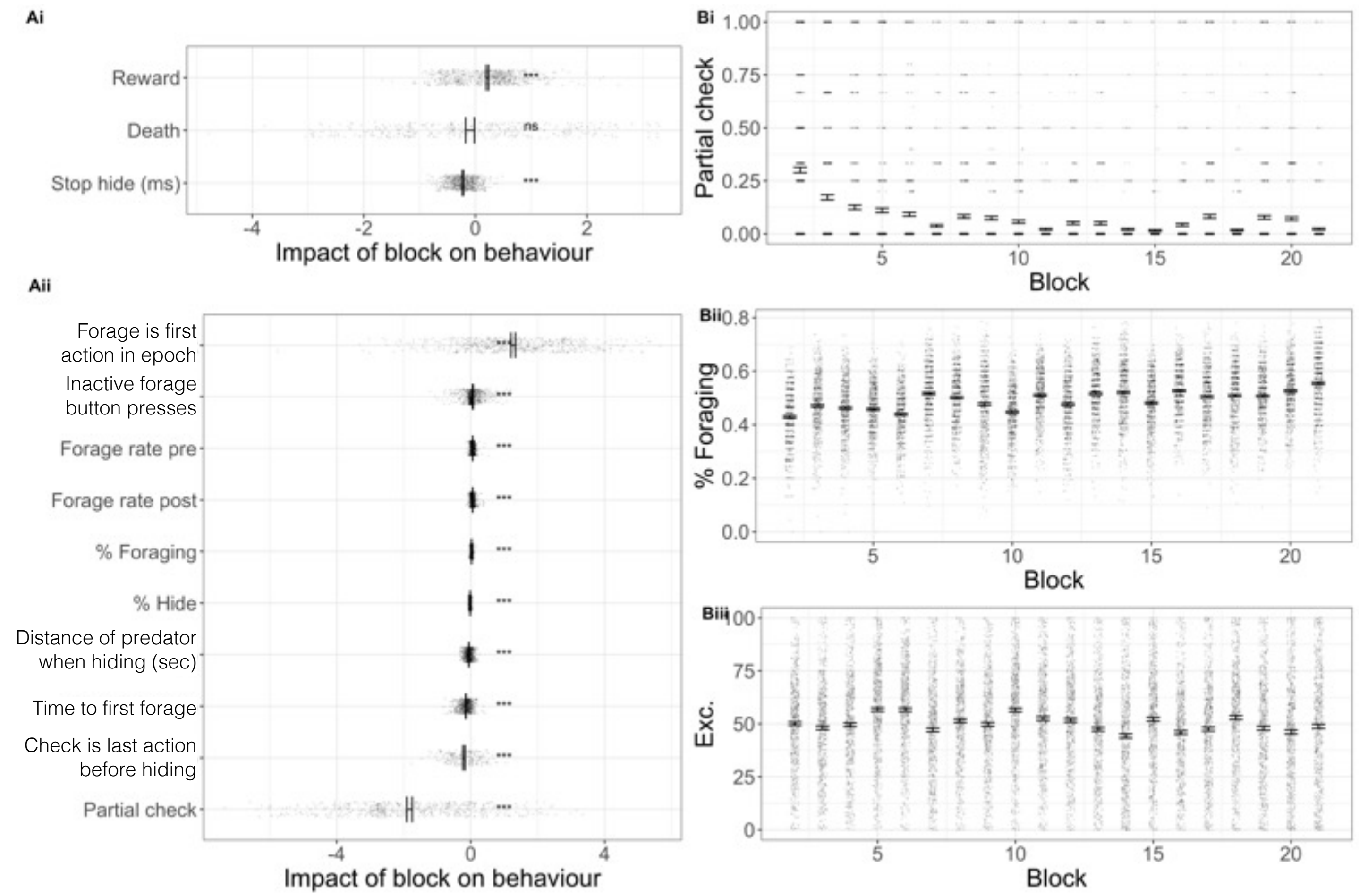


**Figure S4. Participants perform better over time.**

Some behaviours change over the course of the experiment others do not, here we show behaviours that change – assessed as effect of block index in the regressions of aggregate behaviours. Ai) Participants improve across the task, returning sooner from hiding (Stop hide (ms)), and overall gaining more reward, but they don't get caught less often. Aii) Other behaviours that are less clearly 'optimal' also change over time. B) Illustration of some select measures binned across blocks. Note that the reason for many an effect of block is not visible in the raw data is that other factors have a larger impact on behaviour/mood (see figure 2B in main text). Statistical tests in A are one-tailed two-sided t-tests of the regression weights. For further information on task behaviours see table S5 ***$p<0.001$ (no adjustments for multiple comparisons), ns: not significant, grey dots are individual participants, mean and standard errors in black. N=702

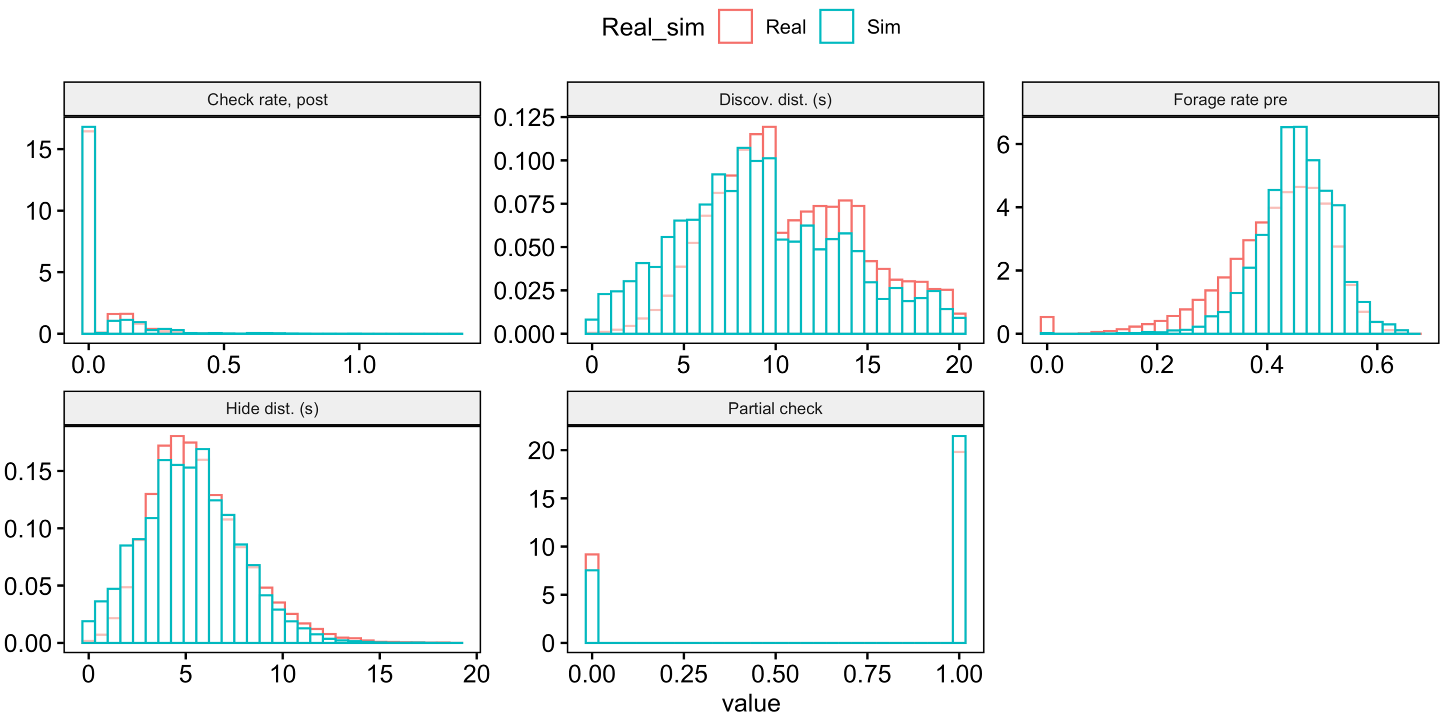


**Figure S5. Histogram of selected behavioural measures comparing real and simulated data (n=702, replication sample).**

Simulated data used the exact parameters fit on the real data (replication sample), but all simulations played the same schedule, while participants performed two types of schedules. Selected measures include: Whether or not partial checks were made prior to discovery of predator, how far (in seconds) the predator was at the time of the discovery, the forage pre discovery, the check rate post discovery and the distance (in seconds) of the predator at the time the participants hid. The most notable divergence is that sometimes real participants do not forage at all before discovering the predator and that the simulations allow the model to get too close before discovering the predator.

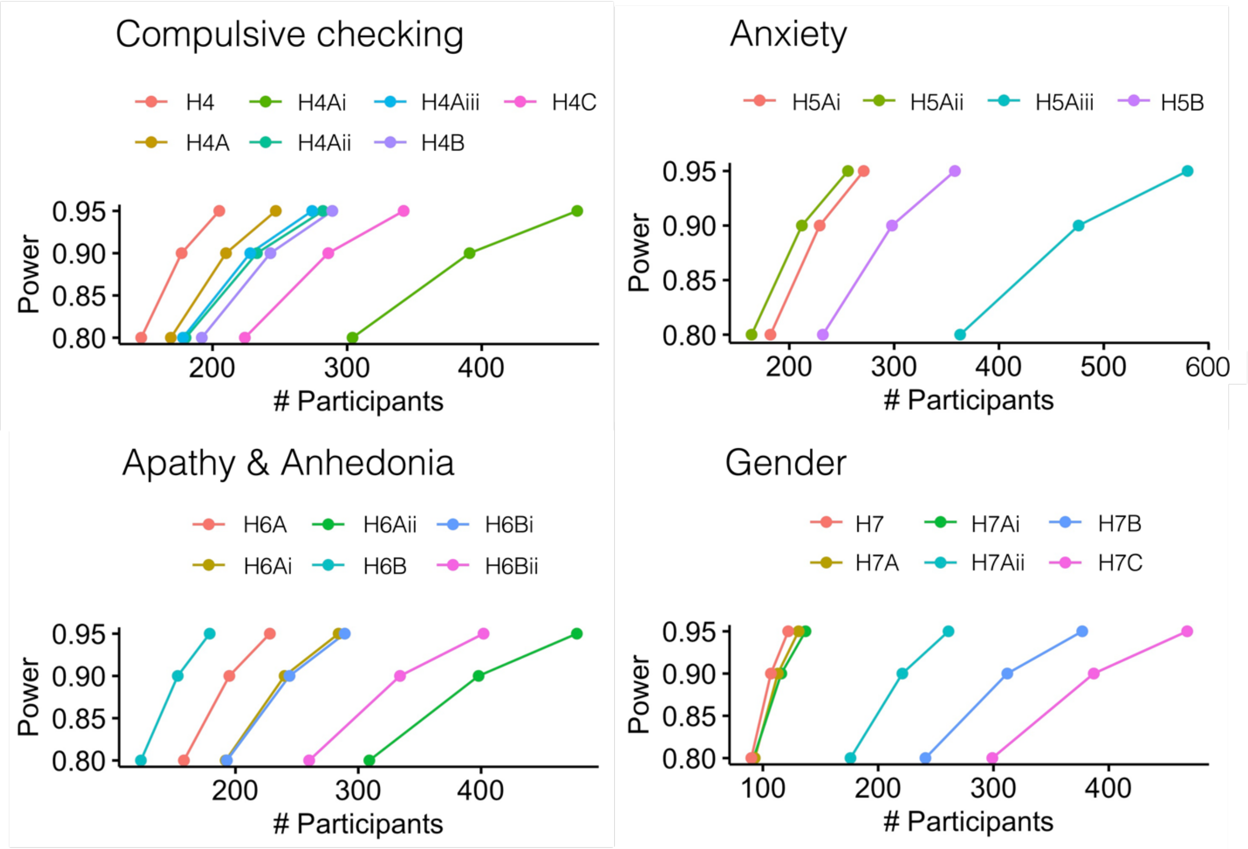


**Figure S6. Power calculation.**
Power as a function of the number of participants for each hypothesis based on discovery sample. After discovery sample analysis, replication sample size was computed for each clinical hypothesis using the WebPower package in R (Zhang & Yuan, 2018). For this, we computed Cohen's f2 for each hypothesis. We computed the number of participants required for power between 0.8 and 0.95. This suggested that 580 participants should provide 95% power for all analyses (for $p < 0.05$, one-tailed).

Ai Factor scores – replication sample

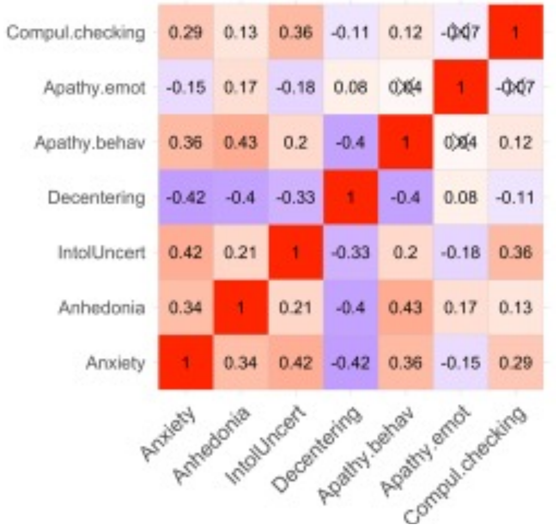


Aii Factor scores – discovery sample

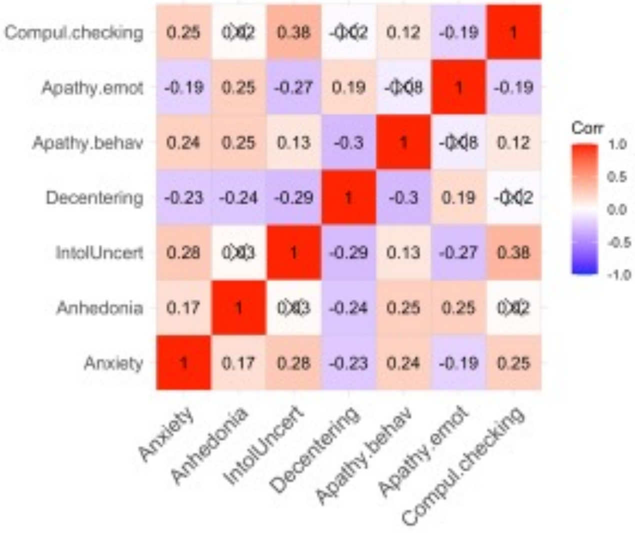


B Questionnaire subscales – discovery sample

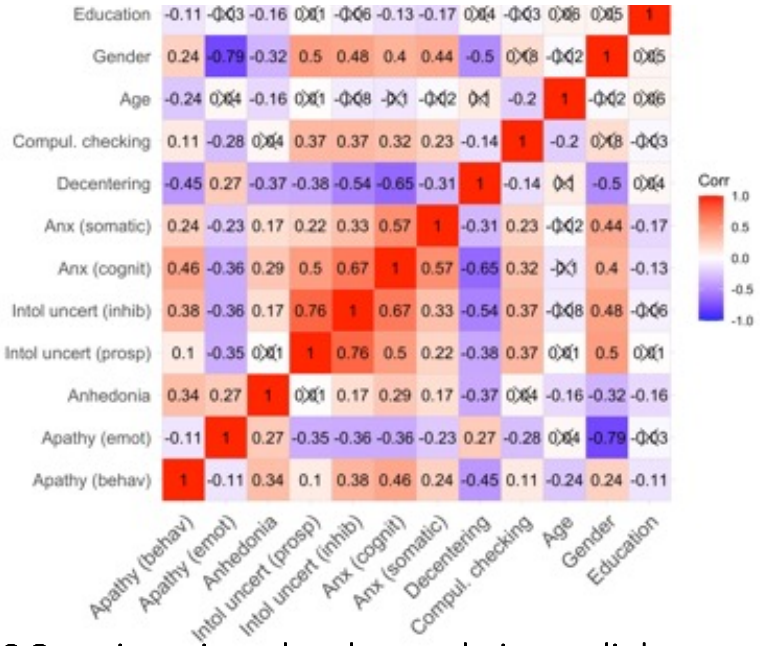


C Questionnaire subscale correlations split by age

Age <=40

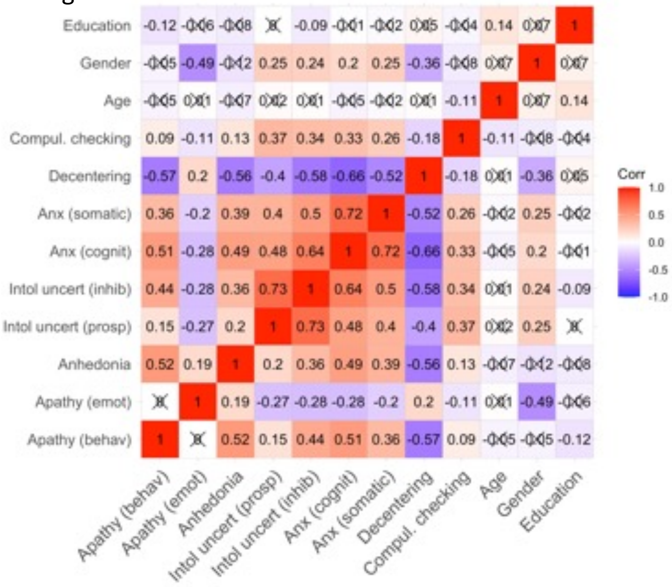


Age >40

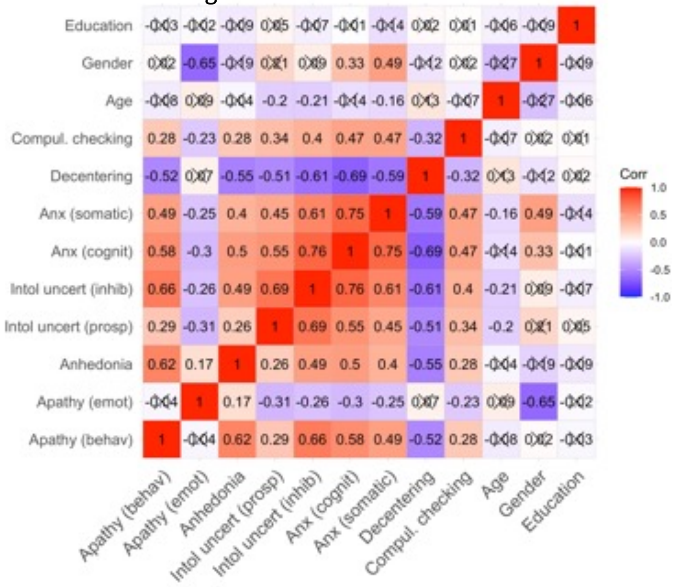

**Figure S7. Correlations among questionnaire factor scores and subscales (related to extended data figures 2+3).**

A) Applying the factor solution from extended data figure 3 to the replication and discovery data creates factors scores for each participants. Correlations are Pearson's r, not corrected for multiple comparisons. The reason that the factor correlations are not exactly the same in the two samples is due to applying the Bartlett method when extracting factor scores (vs. the [default] Thurstone method). The correlations thus reflect the differences in correlations in the questionnaire subscales, see next. Sample size: n=702 (Ai), n=374 (Aii). B) For comparison to extended data figure 2 in the main text, correlations for the questionnaire subscales are shown for the discovery sample (for gender, instead of correlations, t-tests and Cohen's d are shown). Of note is the replication sample shows somewhat higher correlations between some subscales (e.g. apathy to anxiety), but overall correlation patterns are similar. Sample size: 366 for tests including gender (excluding participants who did not select 'male' or 'female') and otherwise 374. C) To check whether the changed age inclusion criteria (see table S2) in the replication sample affected the correlations between the questionnaire subscales, we plotted them split by age, either below or equal to 40 (left, same criterion as discovery sample, n=539) or above 40 (right, n=163). We found that out of 36 correlations, 29 (i.e. 81%) were larger in the sample above than below 40. We also note that even in the sample below or equal to 40, behavioural apathy and somatic anxiety in particular showed larger correlations with other subscales than in the discovery sample in B. Non-significant correlations are marked with 'X' (i.e. $p>0.05$ for Pearson's r, two-tailed); no adjustments for multiple comparisons.

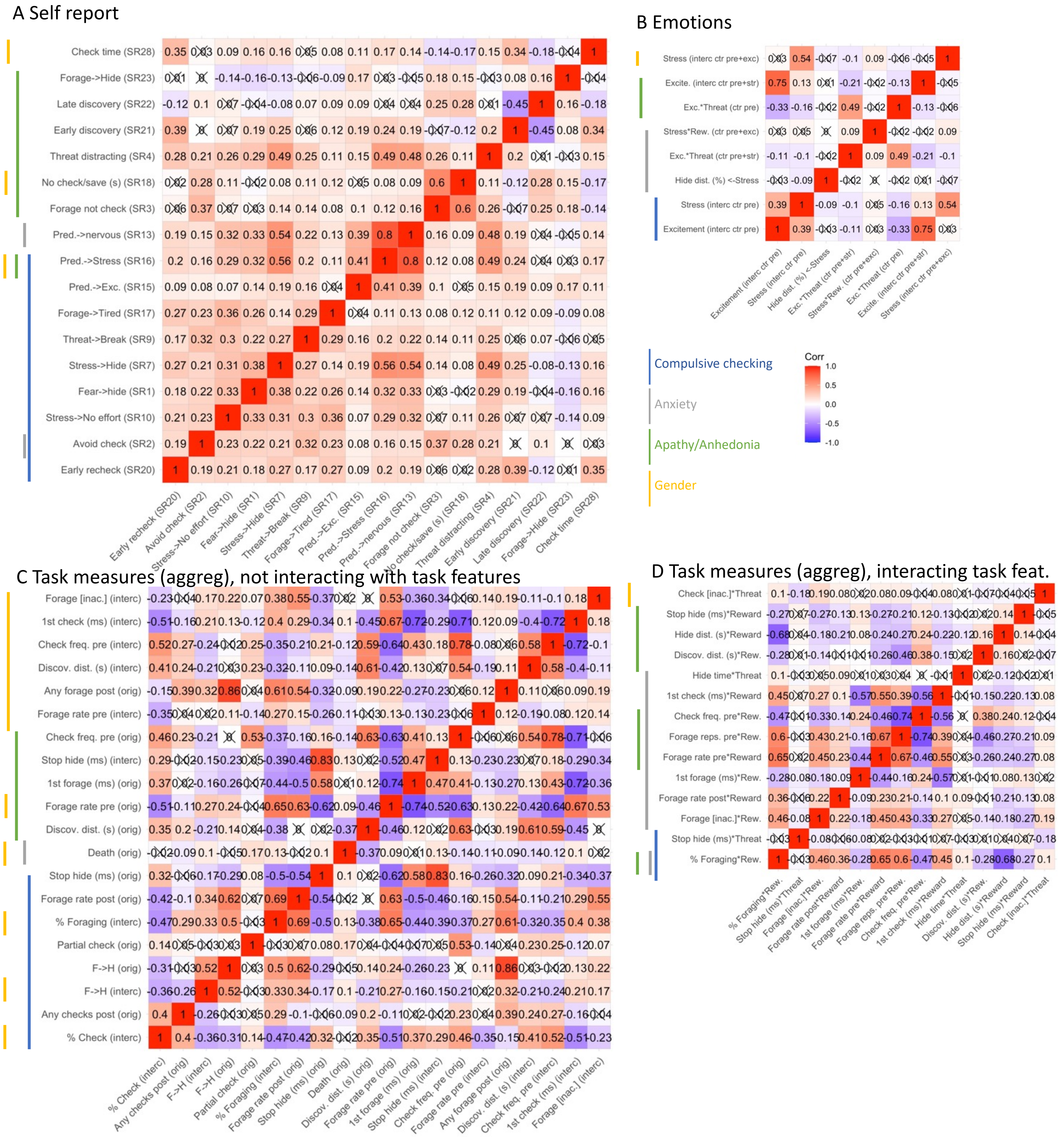


**Figure S8. Correlations between measures included in pre-registration (that were included based on significance of links to clinical dimensions or gender).**

For visualisation split into data pertaining to post-task self reports (A), emotions during the task (B), task behaviours either averaged across all blocks ('orig') or the intercept from the regression predicting behaviour from task features ('interc') (C), task behaviours interacting with task features (reward [rew] or predator speed [threat]) (D). Correlations are shown in colours and with numbers. If not significant, values are crossed out. Coloured bars on the sides show which clinical hypothesis the measures belong to (compulsive checking in

blue, anxiety in grey, apathy/anhedonia in green, gender in yellow). N=702. Non-significant correlations are marked with 'X' (i.e. $p>0.05$ for Pearson's r, two-tailed); no adjustments for multiple comparisons.

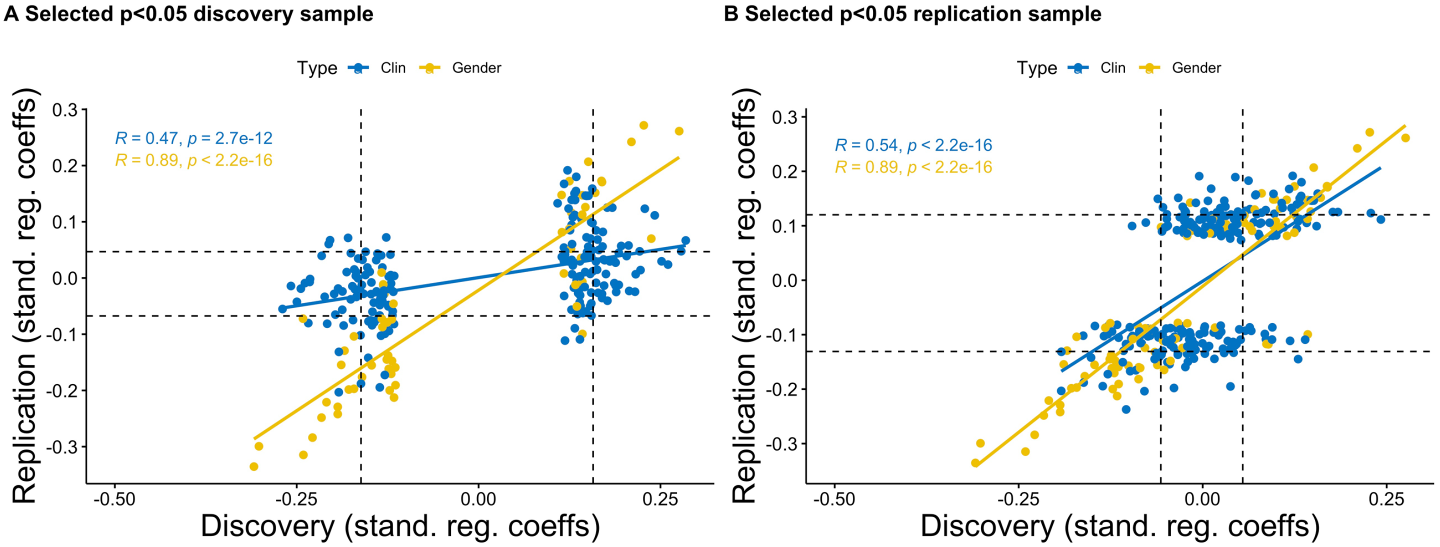


**Figure S9. Correlation between effect sizes of the two samples.**

In both samples, effect sizes (standardized regression coefficients) were selected from analyses of from behaviour/self-report ~ clinical1+ clinical2+ … demographic measures. These were run separately on the two samples. A) Data included were selected based on the regression coefficients being significant (p<0.05) in the discovery sample. B) Data were selected based on the regression coefficients being significant (p<0.05) in the replication sample. Effect sizes were split depending on whether they captured gender effects (yellow) or clinical subscales (blue). Black dotted lines show the means, computed as follows: in A: means of the negative and positive regression coefficients in the discovery sample and for the horizontal lines, means for the replication sample, but grouping of participants according to the sign of each participant in the discovery sample (e.g. if a regression coefficient was negative in the discovery sample, but positive in the replication sample, it would have nevertheless been part of the mean for the negative coefficients for the replication sample). In B, the sorting was reversed. Results shows that there is a correlation between effect sizes in the two samples (significant separately for both effects related to gender and related to clinical subscales). Results also show that sorting of effect sizes reproduces the well-known effect that effect sizes decrease in replication studies.

## SUPPLEMENTARY TABLES

| Exclusion reason | % (total: 773) |
|---|---|
| Self reported task problems | |
| FALSE | 765 (99.0%) |
| TRUE | 8 (1.03%) |
| Button reactivation problem (>6 occurences) | |
| FALSE | 757 (97.9%) |
| TRUE | 16 (2.07%) |
| Button reactivation problem, alternative measure (>6) | |
| FALSE | 772 (99.9%) |
| TRUE | 1 (0.13%) |
| Fewer than 40 check actions | |
| FALSE | 760 (98.3%) |
| TRUE | 13 (1.68%) |
| Final life counter <5 | |
| FALSE | 769 (99.5%) |
| TRUE | 4 (0.52%) |
| Caught more often than 9 times | |
| FALSE | 760 (98.3%) |
| TRUE | 13 (1.68%) |
| No actions in more than 6 epochs | |
| FALSE | 765 (99.0%) |
| TRUE | 8 (1.03%) |
| Total exclusions based on task | |
| FALSE | 723 (93.5%) |
| TRUE | 50 (6.47%) |
| Questionnaire checks: more than 1 point difference on any check questions [max error] (not used) | |
| FALSE | 683 (88.4%) |
| TRUE | 90 (11.6%) |
| Questionnaire checks: more than 1 point difference on average of check questions [mean error] | |
| FALSE | 750 (97.0%) |
| TRUE | 23 (2.98%) |
| Total exclusions task and questionnaires mean error | |
| FALSE | 702 (90.8%) |
| TRUE | 71 (9.18%) |
| Total exclusions task and questionnaires max error (not used) | |
| FALSE | 640 (82.8%) |
| TRUE | 133 (17.2%) |

**Table S1. Participant exclusion criteria.**
A list of all criteria which excluded any participants in the replication sample, as preregistered. Note that we had pre-registered to exclude participants based on the mean error in the check questions being larger than one. This excluded (combined with the task-based measures) only 9% of participants. Had we instead excluded participants for diverging by more than one point on any check item, we would have excluded 17%.

| | Discovery N=374 | Replication N=702 | p.overall |
|---|---|---|---|
| Age | 29.8 (6.16) | 35.4 (11.0) | <0.001 |
| Gender: | | | 0.660 |
| Female | 191 (51.1%) | 355 (50.6%) | |
| Male | 175 (46.8%) | 337 (48.0%) | |
| Other | 8 (2.14%) | 10 (1.42%) | |
| EducationLevel: | | | 0.002 |
| GCSE | 24 (6.42%) | 79 (11.3%) | |
| A-levels | 135 (36.1%) | 195 (27.8%) | |
| Batchelor | 170 (45.5%) | 304 (43.3%) | |
| Masters | 40 (10.7%) | 103 (14.7%) | |
| Doctorate | 5 (1.34%) | 21 (2.99%) | |
| Experiment duration (min) | 60.6 (14.0) | 58.4 (12.3) | 0.010 |
| Training Duration (min) | 9.67 (6.32) | 9.84 (4.28) | 0.654 |
| Task Duration (min) | 35.3 (6.95) | 34.9 (6.90) | 0.357 |
| Questionnaire duration (min) | 9.29 (4.20) | 8.84 (4.02) | 0.091 |
| Psychoactive medication (%yes): | | | 0.202 |
| no | 324 (86.6%) | 586 (83.5%) | |
| yes | 50 (13.4%) | 116 (16.5%) | |
| Clinical anxiety (STICSA): | | | 0.094 |
| Clinical anxiety probable | 82 (21.9%) | 188 (26.8%) | |
| No clinical anxiety | 292 (78.1%) | 514 (73.2%) | |

**Table S2. Replication and Discovery Sample Demographics and Clinical Scores.**
Clinical scores are shown only for the STICSA as it is the only questionnaire for which a validated clinical threshold (>43 for the trait scale; Van Dam et al., 2013) is available. Percentage of participants reporting using any psychoactive medication is shown here; see Table S3 for a more detailed breakdown. We noticed that accidentally, while for the discovery sample, the age range in the Prolific screening form had been chosen as 18-40 years, this filter was not used for the replication sample, resulting in an age range of 18 to 79. This somewhat impacted correlations between clinical subscales, extended data figure 2, supplementary figure S7. Statistics: "p.overall" is the result of chi-squared tests, two-sided; no adjustments for multiple comparisons.

| Medication | Count |
|---|---|
| ADHD medication | 5 |
| Antidepressant | 105 |
| Antipsychotic | 10 |
| Anxiolytic | 23 |
| Epilepsy or Bipolar Disorder | 4 |
| Other | 10 |

**Table S3. Psychoactive Medication Reported in Replication Sample.**

This table shows a total count of medications reported (it was possible for participants to report more than one medication). Overwhelmingly, antidepressants were the most commonly taken medication. Antidepressants included selective serotonin re-uptake inhibitors (e.g. citalopram, fluoxetine, sertraline), tricyclic antidepressants (e.g. amitriptyline), serotonin-norepinephrine reuptake inhibitors (e.g. venlafaxine) and atypical antidepressants (e.g. bupropion). Antipsychotic medication included quetiapine, risperidone, aripiprazole, and promethazine hydrochloride. Anxiolytic medication included beta blockers (propranolol), benzodiazepines (lorazepam, diazepam, alprazolam, clonazepam), anticonvulsant/anxiolytic (pregabalin), and buspirone. ADHD medication included amphetamines (Adderall, lisdexamfetamine, and lisdexamfetamine dimesylate). We tested whether medication (as an objective measure of needing psychiatric intervention) was related to clinical scores using Bayesian regressions in BRMS (medication yes/no ~ STICSA somatic + STICSA cognitive + AMI emotional + AMI behavioural + SHAPS + Compulsive checking). Indeed, there was a significant impact of STICSA somatic (0.11 [0.05; 0.16]) and overall model comparison (for the full model vs. an intercept-only model) based on approximation of leave-one-out cross validation (loo package in BRMS, see main text (Vehtari et al., 2017)) was better (expected log predictive density [elpd] difference: 27.9, standard error: 7.9).

To complement recent findings (Zorowitz et al., 2023), we related whether participants using psychoactive were more likely to be excluded based on the questionnaire exclusion (figure S1), using Bayesian regressions in BRMS: check_question_error_exclusion (yes/no) ~ medication yes/no. This was not the case (95% Bayesian Credible interval: -0.07 [-1.29; +1.0]).

| No. | Question |
|---|---|
| 1 | I hid for longer than necessary because I was afraid to leave the hiding place. |
| 2 | I avoided checking for the predator because I didn't want to make a decision about when to hide. |
| 3 | I avoided checking for the predator because I wanted to keep diving for food. |
| 4 | It was hard to focus on gathering food in a block with a fast predator. |
| 5 | I got caught by the predator because I was distracted by gathering food. |
| 6 | I avoided checking altogether and went straight to hiding instead. |
| 7 | I hid earlier than necessary because I was stressed. |
| 8 | Before a round with a fast predator, I took a longer break than usual. |
| 9 | After a round with a fast predator, a took a longer break than usual. |
| 10 | I thought a round was too stressful, so I tried not to take it too seriously and didn't try hard to gain more lives. |
| 11 | I didn't dive for food until I knew where the predator was located. |
| 12 | I only dove for food when there was no predator in the area. |
| 13 | After seeing a predator I felt more nervous than before. |
| 14 | After seeing a predator I felt more relaxed than before. |
| 15 | After seeing a predator I felt more excited than before. |
| 16 | After seeing a predator I felt more stressed than before. |
| 17 | I hid earlier than necessary because I was tired of diving for food. |
| 18 | I avoided checking for a predator to save time. |
| 19 | I avoided checking for a predator because I was afraid to see one. |
| 20 | I sometimes checked again for a predator even though I had just finished checking in all directions? |
| 21 | I tried to find the predator as early as possible. |
| 22 | I tried to find the predator as late as possible. |
| 23 | I tried to gather as much food as possible right before hiding. |
| 24 | I tried not to check on the predator when it was very close because it felt threatening. |
| 25 | I was not worried about how soon the predator would arrive. |
| 26 | Please explain your answer to Question 6 ("I avoided checking altogether and went straight to hiding instead"). If you sometimes did this, please explain why |
| How much time did you spend performing each of these actions? | |
| 27 | Eating food |
| 28 | Checking for a predator |
| 29 | Hiding from a predator |
| 30 | Did you have any technical issues in playing the game or completing the questionnaires? Please circle one: Yes / No |
| 31 | If you answered "yes" to the previous question, what technical issues did you experience? We appreciate any feedback as we are trying to improve the study. |

**Table S4. Debrief Questionnaire Items.**
Items were rated on a Likert scale from 0 (e.g. “never”) to 6 (e.g. “very often”).

| Variable | Description |
|---|---|
| **A Variables used in pre-registered individual difference hypotheses whose clinical links replicated** | |
| F->H | Type of last action before hiding (1=forage, 0=check) |
| % Foraging | Foraging as a percentage of all actions across the epoch |
| Stop hide (ms) | Time (s) to stop hiding at beginning of epoch before predator discovered |
| **B Additional vars: non-preregistered with significant clinical links across both samples** | |
| % Hide | Hiding as a percentage of all actions across the epoch |
| Hide dist. | Distance of predator when participant hides (in %) |
| Hide dist. (s) | Distance of predator when participant hides (in sec) |
| Hide time (since begin) | Time (s) since epoch began at time of hide |
| Hide [inac.] | Rate of inactive hide button presses across the epoch |
| Total earning | Total earnings in the task |
| Forage first | First action in the epoch before predator discovered (1=forage, 0=check) |
| 1st check (ms) | Time (s) to first check before predator discovered |
| Any forage pre | Whether participants foraged at all (yes/no) before predator discovery |
| % Check | Checking as a percentage of number of checks plus number of forages, across the epoch |
| 1st forage (ms) | Time (s) to first forage before predator discovered |
| Check freq. pre | Frequency of checking sequences before predator discovered |
| Discovery dist. (%) | Percentage of predator journey remaining at time of predator discovery |
| Discov. dist. (s) | Time (s) until predator arrival at time of predator discovery |
| Forage reps. post | Maximum number of forages in a sequence (uninterrupted by checking) after predator discovered |
| F->C pre | Rate of transitions (per sec) from forage to checking before predator discovery |
| Forage reps. pre | Maximum number of forages in a sequence (uninterrupted by checking) before predator discovered |
| Any forage post | Whether any foraging occurred after predator discovery |
| **C Additional vars: pre-registered individual difference hypotheses whose gender links replicated** | |
| Forage rate pre | Rate of foraging before predator discovered |
| Forage rate post | Rate of foraging after predator discovered |
| Death | Number of times the fish was caught by the predator |
| Forage [inac.] | Rate of inactive forage button presses across the epoch |
| Check [inac.] | Rate of inactive check button presses across the epoch |
| **D Additional vars: non-preregistered with significant gender links across both samples** | |
| Reward foraged | Total earnings in the task (ignoring points lost due to being caught) |
| #Times caught | Total number of times caught in the task |
| Any checks post | Whether any checks occurred after predator discovered |
| **E Additional vars: used in pre-registration and did not replicate** | |
| Partial check | Whether any partial checks (not checking in every direction) occurred before predator discovered |
| **F Additional vars: not in pre-registration and no non-preregistered corrs** | |
| Any checks | Whether participants checked at all for predators |
| Hides | Whether participants hid more than once in an epoch |
| Check rate, post | Rate of checking per second after predator discovered |
| (s) b/w checks | Time (s) in between checks after predator discovery |
| C->H dist. perc | Predator distance (%) when participants make their last check in an epoch |
| C->H dist (s) | Seconds until predator arrival at time of last check before hiding |
| (s) b/w checks pre (orig) | Time (s) in between checking sequences before predator discovered |

**Table S5. Explanation of all behavioural measures.**

For visualization, the variable have been grouped, if a variable is included in a group, even if it was also included in a subsequent group, it is only listed once: A) Variables included in the pre-registration as linked to clinical

traits and significant again individually in the replication sample (table S11), B) Variables not pre-registered, but showing significant correlations (or regressions, correcting for other clinical traits) with clinical traits across both samples (table 3), C) Variables in pre-registration linked to gender and significant again individually in the replication sample, D) Variables not pre-registered, but showing links with gender across both samples, E) Variables in pre-registration that did not replicate and not included in any groups A-D. F) Other variables measured that did not show any significant links in pre-registration or post-hoc.

| | Pre-discovery (forage=0, check=1) | | Post-discovery (hide=1, not=0) | | Post-discovery (forage=0, check=1 ) | |
|---|---|---|---|---|---|---|
| **Name** | **Cohen's d** | **p-value** | **Cohen's d** | **p-value** | **Cohen's d** | **p-value** |
| Const | -1.85 | 1.03E-228 | -1.06 | 1.15E-116 | -1.44 | 2.61E-169 |
| Reward | -0.64 | 7.99E-55 | -0.29 | 2.99E-14 | 0.04 | 0.333 |
| Check seq | 2.45 | 6.49E-299 | NA | NA | NA | NA |
| Threat (time pressure) | 1.57 | 2.13E-191 | NA | NA | NA | NA |
| Threat (proximity) | NA | NA | 3.33 | 0 | 0.08 | 0.041 |
| Threat (time since last check | NA | NA | 1.16 | 8.09E-132 | 0.65 | 6.46E-54 |
| Threat (predator speed) | 0.52 | 3.29E-38 | -1.15 | 1.31E-129 | -0.82 | 4.83E-78 |
| # cones | 0.22 | 9.49E-09 | 0.74 | 6.40E-68 | -0.2 | 2.59E-07 |
| Env indx | -0.23 | 1.60E-09 | -0.68 | 3.40E-60 | -0.89 | 1.23E-88 |
| Epoch 1 | 1.67 | 1.50E-204 | 0.63 | 2.47E-53 | 0.41 | 7.86E-25 |

**Table S6. Single-Choice Regression Analyses: Group-Level Results.**
Results of regressions testing the impact of task features on participants' individual choices (rather than summary behavioural measures). Three separate regressions (see main text methods 'Exploratory analyses – Single-choice regressions' were run assessing for each participant the effect of task features on choices to forage or check before the discovery of the predator ('Pre-discovery'), on choices whether to hide or not post discovery and, if not hiding, whether the forage or check post-discovery. Effect sizes are Cohen's and p-values the results of two-tailed t-tests of regression weights across participants. Results are shown here for the replication sample, the signs were the same and the significance similar in the discovery sample (no case where $p<0.05$ in one sample but not the other). Abbreviations: Const: Intercept/constant in the regression; check seq: whether pre-discovery a check sequence has started and not yet ended (e.g. participant has checked one out of four possible areas); time pressure: time since last completed check sequence divided by speed of predator; proximity: proximity of predator in seconds. # cones: number of areas that can be checked: env indx: index of the environment in the task; epoch 1: whether the current choice belongs to the first epoch in an environment or not.

| A) Pre discovery (check or forage) | | | | | | |
|---|---|---|---|---|---|---|
| | *Const_Check.t* | *Reward.t* | *Threat(time pressure).t* | *CheckSeq.t* | *Epoch1.t* | *EnvInd.t* |
| *Const_Check.f* | 0.840*** | -0.036 | 0.453*** | 0.033 | -0.045 | 0.048 |
| *Reward.f* | -0.044 | 0.774*** | 0.05 | -0.012 | 0.002 | 0.025 |
| *Threat(time pressure).f* | -0.016 | 0.028 | 0.974*** | -0.093 | 0.014 | 0.017 |
| *CheckSeq.f* | 0.255*** | 0.095 | -0.047 | 0.902*** | -0.024 | 0.137 |
| *Epoch1.f* | 0.043 | 0.024 | 0.017 | 0.029 | 0.881*** | -0.065 |
| *EnvInd.f* | 0.142 | 0.022 | 0.011 | 0.133 | -0.129 | 0.924*** |
| *Threat(predator speed).f* | 0.257*** | 0.170* | 0.182* | 0.078 | 0.027 | -0.132 |
| *Check dirs.* | -0.098 | 0.232** | -0.033 | 0.164* | 0.077 | -0.056 |

| B) Post discovery (hide or not) | | | | | | |
|---|---|---|---|---|---|---|
| | *Const_Hide.t* | *Reward.t* | *Threat(proximity).t* | *Threat(time since last check).t* | *Epoch1.t* | *EnvInd.t* |
| *Const_Hide.f* | 0.911*** | -0.058 | 0.150* | 0.12 | -0.063 | 0.024 |
| *Reward.f* | -0.223** | 0.560*** | -0.026 | -0.035 | 0.011 | -0.048 |
| *Threat(proximity).f* | 0.113 | -0.092 | 0.848*** | -0.052 | -0.009 | -0.008 |
| *Threat(time since last check).f* | 0.176* | 0.044 | -0.109 | 0.878*** | 0.164* | -0.002 |
| *Epoch1.f* | 0.034 | 0 | -0.03 | 0.134 | 0.579*** | 0.032 |
| *EnvInd.f* | -0.011 | -0.117 | 0.081 | -0.083 | -0.023 | 0.893*** |
| *Threat(predator speed).f* | 0.266*** | 0.011 | 0.08 | -0.055 | 0.051 | 0.129 |
| *Check dirs.f* | 0.192* | -0.035 | 0.068 | -0.029 | -0.1 | 0.033 |

| C) Post disocvery (check or forage) | | | | | | |
|---|---|---|---|---|---|---|
| | *Const_Check.t* | *Reward.t* | *Threat(proximity).t* | *Threat(time since last check).t* | *Epoch1.t* | *EnvInd.t* |
| *Const_Check.f* | 0.797*** | 0.024 | 0.188* | -0.153* | 0.055 | -0.038 |
| *Reward.f* | -0.102 | 0.626*** | -0.076 | -0.058 | 0.007 | -0.047 |
| *Threat(proximity).f* | 0.014 | -0.023 | 0.674*** | 0.185* | 0.017 | -0.023 |
| *Threat(time since last check).f* | 0.068 | -0.124 | -0.039 | 0.852*** | 0.037 | 0.016 |
| *Epoch1.f* | 0.016 | 0.036 | -0.022 | -0.11 | 0.639*** | 0.063 |
| *EnvInd.f* | -0.005 | 0.003 | -0.159* | 0.071 | 0.064 | 0.690*** |
| *Threat(predator speed).f* | 0.419*** | 0.014 | 0.014 | -0.159* | 0.087 | -0.081 |
| *Check dirs.f* | 0.555*** | 0.039 | -0.046 | -0.035 | 0.129 | -0.045 |

**Table S7. Parameter recovery.**

Parameter recovery for models fit on either A) pre-discovery check or forage, B) post-discovery choices to hide or not to hide, c) post-discovery, choices to check or hide. Based on 200 simulations, parameters drawn randomly from replication sample participants, breaking correlations between parameters. Correlations are Pearson's r. Abbreviations: .t: true parameters; .f: fitted parameters; Epoch1: epoch is the first one of an environment or not;

EnvInd: index of the environment in the task; Check dirs.: number of check directions available. While p-values are shown (for Pearson's correlation r, two-sided), the important values to consider are the strengths of correlations (Pearson's r) as the significance depends on the number of simulations which is arbitrary; no adjustments for multiple comparisons.

| Variable | Reward | Threat (speed) | Threat (delay) | #Cones | #Env | 1st epoch (true/false) | Pred. disc. time (sec to end) | Intercept |
|---|---|---|---|---|---|---|---|---|
| Death | 0.11* $ | -0.18*** | -0.08 (ns) & | -0.42*** | -0.06 (ns) | -0.4*** | NA | -2.41*** |
| Discovery dist. (%) | -0.31*** | -0.59*** | 1.03*** | -0.63*** | -0.13*** $ | 0.54*** | NA | 9.11*** |
| Discov. dist. (s) | -0.28*** | -4.14*** | 0.9*** | -0.61*** | -0.08* ? | 0.51*** | NA | 17.73*** |
| Any checks | -0.1 (ns) | -0.08 (ns) | 0.26** | -0.09 (ns) | -0.22** $ | 1.31*** | NA | 2.05*** |
| % Check | -0.46*** | 1.57*** | 1.17*** | 2.11*** | -0.85*** | 1.38*** | NA | 1.85*** |
| % Foraging | 0.5*** | -1.54*** | 0.98*** | -1.98*** | 1.21*** | -0.22*** | NA | 6.31*** |
| % Hide | -0.35*** | 0.23*** | -1.31*** | 0.62*** | -0.76*** | -0.77*** | NA | 3.17*** |
| Hides | 0.02 (ns) | -0.92*** | -0.09 (ns) & | -0.38*** | -0.49*** | -0.54*** | NA | -2.37*** |
| Hide dist. (%) | -0.21*** | 2.14*** | 0.43*** | 0.89*** | -0.57*** | 0.26*** | 1.57*** | 2.81*** |
| Hide dist. (s) | -0.17*** | -0.34*** | 0.33*** | 0.81*** | -0.54*** | 0.21*** | 1.67*** | 3.95*** |
| Hide time (since begin) | -0.03 (ns) | -5.44*** | 6.13*** | -0.74*** | 0.63*** | -0.56*** | -1.47*** | 22.15*** |
| Hide [inac.] | 0.22*** | -0.11** $ | 0.02 (ns) | -0.24*** | -0.31*** | -0.18*** | NA | -0.4*** |
| Check [inac.] | -0.1** | 0.81*** | 0.67*** | 1*** | -0.31*** | 0.58*** | NA | 0.75*** |
| Forage [inac.] | 0.47*** | -0.59*** | -0.39*** | -0.6*** | 0.24*** | -1.05*** | NA | 1.06*** |
| Any checks post | 0.01 (ns) | -0.55*** | 0.14*** $ | -0.83*** | -0.24*** | 0.21*** $ | 1.85*** | -0.7*** |
| Check rate, post | -0.07 (ns) | 0.07 (ns) | 0.1 (ns) | 0.09 (ns) | -0.68*** | 0.37*** | -1.11*** | -4.25*** |
| (s) b/w checks | -0.03 (ns) | -0.16** | -0.28*** | -0.6*** | 0.66*** | -0.15** | 1.1*** | 0.76*** |
| Any forage post | 0.14*** | 0.85*** | -0.27*** | -0.1* ? | 0.63*** | 0.18*** | 3.57*** | 1.65*** |
| Forage rate post | 0.14*** | 0.14*** | -0.2*** | -0.48*** | 0.92*** | -0.35*** | 1.27*** | -3.44*** |
| Forage reps. post | 0.31*** | 0.21*** | -0.41*** | -0.42*** | 0.89*** | -0.91*** | 3.49*** | 2.38*** |
| F->H | 0.19*** | 0.64*** | -0.24*** | 0.06 (ns) & | 0.52*** | 0.05 (ns) & | 1.41*** | 0.96*** |
| C->H dist. perc | 0.02 (ns) | 1.68*** | 0.07 (ns) & | 0.61*** | -0.45*** | 0.29*** | 1.28*** | 3.41*** |
| C->H dist (s) | 0.01 (ns) | -0.78*** | 0.07 (ns) & | 0.55*** | -0.45*** | 0.29*** | 1.5*** | 4.84*** |
| Partial check | 0.58*** | -0.71*** | 0.65*** | -0.19*** $ | -0.96*** | 0.36*** | NA | -2.55*** |
| (s) b/w checks pre (orig) | 0.11* $ | -1.24*** | 0.22*** | -0.67*** | 0.21*** | 0.15*** | NA | 2.25*** |
| F->C pre | -0.12** $ | 1.77*** | 0.57*** | -0.46*** | -0.49*** | 1.03*** | NA | 3.21*** |
| Check freq. pre | -0.46*** | 2.11*** | 0.28*** | -0.7*** | -0.81*** | 1.96*** | NA | -4.9*** |
| Forage first | 0.09* | -0.08* | -0.2*** | 0.58*** | 0.8*** | -1*** | NA | 1.27*** |
| 1st check (ms) | 0.45*** | -1.58*** | 0.01 (ns) | 0.04 (ns) | 0.72*** | -0.73*** | NA | 11.76*** |
| 1st forage (ms) | -0.23*** | 0.04 (ns) ! | 0.25*** | 0.31*** | -0.86*** | 0.93*** | NA | 11.42*** |
| Stop hide (ms) | -0.45*** | -0.54*** | 0.03 (ns) | 0.29*** | -1.07*** | -0.03 (ns) | NA | 7.75*** |
| Any forage pre | 0.3*** | 0.69*** | 1.99*** | 0.11 (ns) | 0.89*** | 1.52*** | NA | 3.12*** |
| Forage rate pre | 0.56*** | -0.85*** | 0.34*** | -1.83*** | 1.02*** | 0.23*** | NA | -4.67*** |
| Forage reps. pre | 0.57*** | -2.25*** | 0.43*** | -0.52*** | 0.71*** | -0.85*** | NA | 4.81*** |

**Table S8. Impact of task features on behaviour (related to figure 2B).**
Epoch-wise behavioural measures (table S5) were predicted based on task features (main text methods 'Impact of task features on behaviour'): amount of reward available in the epoch (Reward), speed of the predator (Threat (speed), monotonic factor), delay from the start until the predator entered (Threat (delay)), number of visibility cones (#Cones monotonic factor), index of the block in the task (#Env), whether the current epoch was the first epoch in a block (1st epoch (true/false)), time until arrival of the predator at the point of predator discovery, i.e. duration of the post-discovery phase (Pred. disc. Time).*p<0.05, **p<0.01, ***p<0.001 for two-tailed one-sample tests on replication sample. Differences (either in terms of direction of the effect or significance) from discovery sample are shown as: $: effect had same sign in both samples, but only significant in replication sample; &: effect had same sign in both samples, but only significant in discovery sample; ?:

effect had opposite sign in both samples and only significant in replication sample; !: effect had opposite sign in both samples and only significant in discovery sample. There were no cases with effects being significant in both samples and of opposite signs. Of note, for a few regressors (Death, Hides), for each subject the distribution of events was very skewed (i.e. events were very rare), therefore for many participants these models could not be estimated, while for others the results may be skewed. None of these regression weights were used in pre-registered hypotheses or post-hoc found to be related to real-life individual differences.

| Hypothesis | Regression type | Measures included in tests | Measures individually significant |
|---|---|---|---|
| H1A | Predict excitement | #Cones [+]; Threat [+]; Reward (max) [+] | #Cones; Threat; Reward (max) |
| H1B | Predict stress | #Cones [+]; Threat[+]; Rew. (max)[+]; Reward (min) | #Cones; Threat; Reward (max) |
| H1C | Predict stress or excitement | Stress predicted by virtual death [+]; Excitement predited by virtual death[-] | Stress predicted by virtual death]; Excitement predited by virtual death |
| H2Ai | Stress as predictor | Discover predator sooner (i.e.distance (sec) of predator when discovered [+]); Forage rate pre predator discovery)[+]; % time spent checking[-]; Time to 1st forage (sec) from the beginning of an epoch<-Stress (ctr e)[-] | Discover predator sooner (i.e.distance (sec) of predator when discovered)); Forage rate pre predator discovery); % time spent checking); Time to 1st forage (sec) from the beginning of an epoch |
| H2Aii | Predict stress | Check frequency pre predator discovery[+]; Distance (sec) of predator when discovered[-]; % of time spent foraging [-]; Forage rate post predator discovery [-]; Last action before hiding is forage [-]; Forage rate pre predator discovery[-]; Length of forage sequences [-] | Distance (sec) of predator when discovered; % of time spent foraging; Forage rate post predator discovery; Last action before hiding is forage; Forage rate pre predator discovery; Length of forage sequences [-] |
| H2B | Stress as predictor | Predator distance when hiding (s)[+]; Any checks post predator discovery [-]; Length of forage sequences [-] | Predator distance when hiding (s) |
| H3A | Excitement as predictor | Rate of pressing the forage button when inactive[+] | |
| H3B | Predict excitement | Rate of pressing the inactive Hide [+] or Forage [+] buttons | Rate of pressing the inactive Hide or Forage buttons |

**Table S9. List of predictors included in mood-related hypotheses (related to table 1).**
Measures that were included in each hypothesis based on the discovery sample ('Measured included in tests') and those that were significant in the replication sample ('Measures individually significant'). The measures were taken from regression models: task to mood (H1A, H1B, H1C), mood to behaviour (H2Ai, H2B, H3A) or behaviour to mood (H2Aii, H3B). As preregistered, the models controlled for the other emotion, i.e. stress or excitement, measured at the same time as the emotion of interest (H1, H2Ai, H3A) and also for the same emotion at the previous measurement point (H2Aii, H3B). For H2B, we did not control for the other emotion. Significance was determined using one-tailed t-tests or Wilcoxon tests (see methods), with significance defined as $p<0.05$. [+] and [-] indicate the direction of the effect in the discovery sample. Specifically, the regression weights for each person within one hypothesis (e.g. for H1A, there were 3: Exc.<-#Cones [+]; Exc.<-Threat [+]; Exc.<-Reward [+]) were added together (with signs flipped if noted by [-]).Then a Wilcoxon or t-test was performed across participants., see methods section 'Mood hypothesis testing'. Note (see methods section 'Divergence from pre-registration') that for H2B, the measure of any check post <-Stress was in fact not significant in the discovery sample after correction of a bug, but nevertheless included here as pre-registered.

| Hypothesis | Discovery sample model | Replication sample model | beta [Bayesian 95% CI] |
|---|---|---|---|
| H1A | Excite.~ 1+ #Check dirs + Speed + Reward (max) + Caught | Excitement (true) ~ Excitement (predicted) | 0.9 [0.74; 1.06] |
| H1B | Stress~ 1+ #Check dirs + Speed + Reward (max) + Reward (min) + Caught | Stress (true) ~ Stress (predicted) | 1.0 [0.91; 1.1] |
| H2Ai | | Behav (residual) ~ Stress*Behav type | 0.03 [0.01; 0.05] |
| H2Aii | Stress (res) ~ Behav 1 (res) + Behav 2 (res) +… | Stress (res, true) ~ Stress (pred) | 0.39 [0.24; 0.54] |
| H2B | | Behav (residual) ~ Stress*Behav type | 0.02 [0.001; 0.04] |
| H3B | Excite. (res) ~ Behav 1 (res) + Behav 2 (res) +… | Excitement (true) ~ Excitement (predicted) | 0.48 [0.16; 0.82] |

**Table S10. Alternative statistical procedures for mood hypotheses**

Statistical tests for the replication sample were (broadly) pre-registered as statistical tests (t-test or Wilcoxon, specifically pre-registered for each case) across regression weights of separate regressions performed for each person (summed within each person), either predicting task from mood or behaviour or predicting behaviour from mood (see methods). The advantage of this approach was its simplicity and that hierarchical tests could easily be constructed by combining regression weights (similar to an ANOVA). On reviewer request, we have developed an alternative approach, applied to exactly the same behaviours and moods as in the pre-registration. Overall, we find that all analyses remain significant. For models predicting mood as a function of several behavioural or task measures (H1A, H1B, H2Aii, H3B), we trained models on the discovery sample (note that for H3Aii and H3B, residuals for mood and behaviour were used, as in the pre-registered analyses that controlled for the task features of H1A). These models were then applied to the discovery sample and predicted stress/excitement related to true stress/excitement. Significance was defined as the 95% Bayesian Credible interval for the regressor predicted Stress/Excitement (column 'beta') not including zero. For models predicting behaviour based on mood (H2Ai and H2B), to construct a single model, the different behaviours were concatenated for each person and a categorical regressor behavioural type was included. The models were fit directly on the replication sample (after selection of the behaviours). Significance was defined was the 95% Bayesian Credible interval for the regression weight for Stress (column 'beta') not including zero.
All models were hierarchical models, i.e. included terms like (1+# Check dirs. + Speed + reward (max) + Caught | ID) in BRMS.

| Hyp | Subscale | Included Regressors | Replicated (corrected for other clinical dimensions) | Replicated additionally, not correcting other clin | Replicated additionally, corrected other clinical, participants aged ≤40 |
|---|---|---|---|---|---|
| 4Ai | Comp. check. | % Check (interc)[gr+, +], Any checks post predator discovery (orig)[+],Last action before hide is forage (interc and original )[gr+, -] | Last action before hide is forage (orig)[-] | | Last action before hide is forage (interc and original )[gr+, -] |
| 4Aii | | Any partial check (orig)[+], Checked again even though just checked (SR20)[+], Avoid checking because didn't want to make decision (SR2)[+] | Checked again even though just checked (SR20)[+], Avoid checking because didn't want to make decision (SR2)[+] | | |
| 4Aiii | | % Foraging across epoch (interc)[gr+, -], Forage rate post predator discovery (orig)[-], % Foraging*Reward [gr+, +], If the round was too stressul, I made no effort (SR10)[+] | % Foraging across epoch (interc)[-] | Forage rate post predator discovery (orig)[-], If the round was too stressul, I made no effort (SR10)[+] | |
| 4B | | Time to stop hiding from epoch start (ms) (orig)[+], Stop hide (ms)*Threat[gr-, +], Hiding for longer than necessary because afraid (SR1)[+], I hid earlier than necessary because stressed (SR7)[+], Took a longer break after round with fast predator (SR9)[+], Hid earlier than necessary because tired (SR17)[+] | Hiding for longer than necessary because afraid (SR1)[+], Took a longer break after round with fast predator (SR9)[+], Hid earlier than necessary because tired (SR17)[+] | I hid earlier than necessary because stressed (SR7)[+] | |
| 4C | | Excitement (interc )[+], Stress (interc )[+], After seeing predator felt more excited (SR15)[+], After seeing predator felt more stressed (SR16)[+] | Excitement (interc pre)[+] | After seeing predator felt more excited (SR15)[+], After seeing predator felt more stressed (SR16)[+] | |
| 5Ai | IU pros | Intearctions (*Reward) between the amount of reward and: % Foraging[gr+, -], Inactive forage button presses [gr+, -], Forage rate post predator discovery [gr+, -], Time to first forage from epoch start [gr-, +], Forage rate pre predator discovery [gr+, -], Maximum forage action sequence length pre discovery [gr+, -], Check frequency pre predator discovery[gr-, +], Time to first check from epoch start [gr+, -] | | | |
| 5Aii | IU inh | Hide time (since beginning of the epoch) *Threat[gr-, +], Action to return from hiding from the beginning of the epoch *Threat[gr-, -], Impact of stress on the predator distance when hiding [-] | | | |
| 5Aiii | IU pros | Imapct of threat on excitement[+], Impact of reward on stress[+] | | | |
| 5B | Anx som | Number of times player caught by predator (orig)[+], % Check of check and forage actions in an epoch (interc)[gr+, -], Avoid checking to dive for food (SR2)[+], Feeling more nervous after seeing predator (SR13)[+] | Avoid checking to dive for food (SR2)[+], Feeling more nervous after seeing predator (SR13)[+] | | |
| 6Ai | Beh apath | Distance of predator at time of discovery (s) (orig)[-], % Foraging of all actions (interc)[gr+, +], Forage rate pre discovery (orig)[+], Time to 1st forage in epoch(orig)[-], Time to stop hiding (interc)[gr+, -], Check freq. pre predator discovery (orig)[-], Avoid checking to forage (SR3)[+], Avoid checking to save time (SR18)[+] | Avoid checking to forage (SR3)[+] | Time to stop hiding (interc) (interc)[-] | % Foraging of all actions (interc)[+], Time to 1st forage in epoch(orig)[-] |
| 6Aii | | Impact of threat on excitement[+], More stressed after seeing predator (SR16)[+], Threat made it hard to gather food (SR4)[+] | | More stressed after seeing predator (SR16)[+] | Impact of threat on excitement[+] |
| 6Bi | Anhedonia | Interactions with reward (*reward): Predator distance at discovery (s)[gr-, -], Predator distance when hiding (s)[gr-, -], Check freq. pre predator discovery [gr-, -], Time to stop hiding [gr-, -], Forage rate pre discovery[gr+, +], Maximum forage action sequence length pred discovery [gr+, +], % Foraging[gr+, +] | Time to stop hiding*Reward[-] | | % Foraging*Reward [+] |
| 6Bii | | Tried to find predator as quickly as possible (SR21)[-], Tried to find the predator as late as possible (SR22)[+], Tried to gather as much food as possible before hiding (SR23)[-], Excitement during the task (interc)[-] | Tried to find predator as quickly as possible (SR21)[-], Tried to gather as much food as possible before hiding (SR23)[-], Excitement during the task (interc)[-] | | Tried to find the predator as late as possible (SR22)[+] |
| 7Ai | Gender | % Foraging (interc)[gr+, -], Forage rate pre discovery (orig and intercept)[-], Any forage post disocvery (orig)[-], Forage rate post discovery (orig)[-] | % Foraging (interc)[-], Forage rate pre discovery (orig and intercept)[-], Any forage post disocvery (orig)[-], Forage rate post discovery (orig)[-] | | |
| 7Aii | | % Check (interc)[gr+, +], Predator distance at discovery (s) (interc)[gr+, +], Check freq. pre discovery (interc)[gr-, +], Time to fist check (interc)[gr+, -], F->H (interc)[gr+, -], Time spent checking for predator (SR28)[+], Avoid checking to save time (SR18)[-], Number times caught by predator (orig)[+] | % Check (interc)[+], Predator distance at discovery (s) (interc)[+], Check freq. pre discovery (interc)[+], Time to fist check (interc)[-], F->H (interc)[-], Time spent checking for predator (SR28)[+], Avoid checking to save time (SR18)[-], Number times caught by predator (orig)[+] | | |
| 7B | | Stress (interc)[+], Feeling more stressed after seeing predator (SR16)[+] | | Stress (interc)[+] | |
| 7C | | Inactive button presses for: foraging (interc)[gr+, -], Forage*Rew.[gr+, -], checking*Threat[gr+, -], | Inactive button presses for: foraging (interc)[-], Forage*Rew.[-], checking*Threat[-], | | |

**Table S11. List of predictors included in individual difference (clinical, age) hypothesis (related to table 2).**

Measures that were included ('Included Regressors') in each hypothesis based on the discovery sample and those that were significant in the replication sample controlling for other clinical subscales ('Replication (corrected for other clinical dimensions)'). Additional regressors that were replicated are shown for (each time, only the additional significant replications are shown): not correcting for other clinical subscales ('Replicated additionally, not correcting other clin') and only in participants fulfilling the same age restrictions as in the discovery sample, i.e. ≤40, correcting for other clinical dimensions ('Replicated additionally, corrected other clinical, participants aged ≤40') – see figure S7C for how the questionnaire subscales were more correlated in older participants. In the left- most column the group level effects (where appropriate, i.e. for regression weights) are also printed (e.g. 'gr-'). [+] and [-] indicate the direction of the relationship between the clinical subscale and the task measure in the discovery sample. Significance of individual predictors was tested by running the same regressions as in the discovery sample of the form: $m_i \sim \beta_0 + \beta_1 \text{targetClinical} + \beta_2 \text{clinical1} + \beta_3 \text{clinical2} + \beta_4 \text{demographic1} \dots$ (where $m_i$ is the predictor; see Eq. 7 in the main text). In other words, we tested whether there was a unique relationship between a clinical predictor and a behaviour (or self-report measure), controlling for other clinical or demographics dimensions). Significance was defined as $p<0.05$, one-tailed. For general hypotheses (e.g. H4 or H4A), full included predictors are not listed because the predictors were simply all those from the relevant sub-hypotheses. Behaviour measures are explained in more detail in table S5. Abbreviations: (interc): intercept of the regression analysis linking task features to mood or behaviour; (orig): measure averaged across all blocks, rather than included in regressions; SR: 'self report'. Note that analyses on the participants aged ≤40 identified additional behavioural measures, especially for the apathy and anhedonia subscales that replicated, even though the sample size was smaller (only at most 539 participants compared to the 702 in the full sample, i.e. 77% of sample size), supporting the idea that the pre-registered analyses identified fewer individual task measures as replicating due to increased correlations in the replication sample among the questionnaires due to the accidental removal of the upper age limit.

| Hyp | Clin subscale / gender | Bayes Factor | Out-of-sample $R^2$ against null | | Out-of-sample $R^2$ against other clinical | | Model trained on task residuals controlling for other clinical dims | | Excluding participants with any errors on questionnaire quality | | |
|---|---|---|---|---|---|---|---|---|---|---|---|
| | | | $R^2$ | p (1-tailed) | $R^2$ | p (1-tailed) | reg coef | p (1-tailed) | r | p (1-tailed) | #subj |
| 4 | Comp. check. | 5578 | -0.03 | <1e-04 | -0.08 | 0.4 | 0.09 | 0.02638 | 0.26 | <1e-04 | 278 |
| 4A | | 664 | 0 | <1e-04 | -0.03 | 0.09 | 0.1 | 0.01069 | 0.22 | 0.00014 | 280 |
| 4Ai | | 0.066 | -0.05 | 0.34 | -0.02 | 0.76 | 0.02 | 0.33 | 0.1 | 0.05 | 288 |
| 4Aii | | 5.00E+06 | 0.04 | <1e-04 | 0.01 | <1e-04 | 0.16 | 0.00011 | 0.26 | <1e-04 | 294 |
| 4Aiii | | 0.148 | -0.05 | 0.13 | -0.04 | 0.91 | 0.04 | 0.21 | 0.08 | 0.09 | 286 |
| 4B | | 805 | -0.01 | <1e-04 | -0.02 | 0.34 | 0.1 | 0.01085 | 0.26 | <1e-04 | 294 |
| 4C | | 14 | -0.02 | 0.0008 | -0.02 | 0.7 | 0.04 | 0.18 | 0.16 | 0.00251 | 292 |
| 5 | Anx/IU | NA | 0 | 0.0006 | -0.07 | 1 | NA | NA | NA | NA | NA |
| 5A | Int unc | NA | 0 | 0.55 | -0.09 | 1 | NA | NA | NA | NA | NA |
| 5Ai | IU pros | 0.101 | -0.06 | 0.23 | -0.07 | 0.55 | 0.03 | 0.29 | 0.02 | 0.4 | 286 |
| 5Aii | IU inh | 0.034 | -0.06 | 0.7 | -0.07 | 1 | -0.04 | 1 | -0.09 | 0.94 | 292 |
| 5Aiii | IU pros | 0.032 | -0.04 | 0.75 | -0.05 | 1 | -0.03 | 1 | -0.1 | 0.95 | 292 |
| 5B | Anx som | 3.00E+05 | 0.04 | <1e-04 | -0.01 | 0.07 | 0.1 | 0.01896 | 0.24 | <1e-04 | 293 |
| 6 | Apath/An h | NA | 0 | <1e-04 | -0.07 | 0.3 | NA | NA | NA | NA | NA |
| 6A | Beh apath | 5 | -0.06 | 0.0026 | -0.09 | 0.99 | 0.04 | 0.23 | 0.18 | 0.00137 | 291 |
| 6Ai | | 1 | -0.04 | 0.009 | -0.06 | 0.73 | 0.08 | 0.04761 | 0.13 | 0.0143 | 293 |
| 6Aii | | 0.592 | -0.03 | 0.0282 | -0.05 | 1 | -0.04 | 1 | 0.12 | 0.02177 | 292 |
| 6B | Anhedoni a | 97 | -0.03 | <1e-04 | -0.06 | 0.0132 | 0.13 | 0.00217 | 0.15 | 0.00627 | 292 |
| 6Bi | | 0.254 | -0.06 | 0.08 | -0.07 | 0.62 | 0.01 | 0.38 | 0.05 | 0.21 | 294 |
| 6Bii | | 1320 | 0.03 | <1e-04 | 0.03 | <1e-04 | 0.19 | <1e-04 | 0.2 | 0.00029 | 292 |
| 7 | Gender | 6.00E+15 | 0.04 | <1e-04 | 0.06 | <1e-04 | 0.58 | <1e-04 | 0.37 | <1e-04 | 276 |
| 7A | | 6.00E+14 | 0.09 | <1e-04 | 0.07 | <1e-04 | 0.59 | <1e-04 | 0.41 | <1e-04 | 278 |
| 7Ai | | 1.00E+17 | 0.08 | <1e-04 | 0.07 | <1e-04 | 0.58 | <1e-04 | 0.37 | <1e-04 | 284 |
| 7Aii | | 7.00E+06 | 0.05 | <1e-04 | 0.06 | <1e-04 | 0.39 | <1e-04 | 0.33 | <1e-04 | 286 |
| 7B | | 44 | -0.02 | 0.014 | -0.02 | 0.31 | -0.07 | 1 | 0.11 | 0.02859 | 290 |
| 7C | | 4.00E+10 | 0.05 | <1e-04 | 0.05 | <1e-04 | 0.44 | <1e-04 | 0.28 | <1e-04 | 292 |

**Table S12. Additional Statistics Clinical and Demographic Hypotheses (Related to Table 2).**
Bayes factors for the correlations reported in the main text. Bayes factor is a measure that quantifies evidence for and against the null model; Bayes Factor > 1 signifies that the alternative hypothesis is more likely than the null hypothesis (Morey et al., 2022; Rouder et al., 2009); Strength of evidence: >3 'substantial, >10 'strong' and >100 'decisive'. Correlations shown in the main text, Bayes factors here and out-of-sample $R^2$ values against null test the replicability of links between task/self-report measures and clinical subscales (or gender). We can additionally test whether these links are unique for a given clinical dimensions, i.e. controlling for other non-related dimensions. We can do this in two ways. First (column 'Model trained on task residual controlling for other clinical dims'), we can instead of correlating predicted clinical subscales (based on the hypothesis-regressions), or % accuracy for gender, with actual subscales/gender, use a regression of the format predicted

clinical value ~ true clinical value + other clinical subscales + demographics. In this case, to generate the predicted clinical value, models were trained on behaviours after orthogonalized for other clinical subscales, predicting clinical subscales (also orthogonalized for other clinical subscales and demographics). Orthogonalization was used to remove shared variance. Regressions linking Predicted and True clinical subscales used then the raw (unorthogonalized) subscales. This result is sometimes not defined (NA) because the hypothesis was based on combining across different clinical subscales, in which case no single regression weight exists to test it. However, in each case, the components can be tested (e.g. 5A cannot be tested in this way, but 5Ai, 5Aii and 5Aiii can be tested). We report here the standardized regression coefficients and the one-tailed p-values for the regression coefficients (set to 1 if the direction of the effect was not positive). We find that for many hypotheses, the results are the same, other than H4C, H6A (driven by changes to H6Aii, while H6Ai remains significant), H7B.

Second (column 'Out-of-sample $R^2$ against other clinical' – what we had initially pre-registered), we can compare models trained on the discovery sample in the form clinical ~ 1+behav1+ behave 2+ … + other clinical + gender vs. a model of the form clinical ~ 1 + other clinical + gender. We can compare the out-of-sample performance of these models using out-of-sample $R^2$ ($R^2_{OOS}$ ). $R^2_{OOS}$ p values indicate the significance of the out-of-sample $R^2$ value assessed with permutation testing (see Berry et al., 2019). Significance was defined as one-tailed 95% (i.e., null hypothesis was rejected if the true $R^2_{OOS}$ was larger than 95% of $R^2_{OOS}$ derived from predicting the permuted scores). The column 'against null model', shows $R^2_{OOS}$ for models matching table 2 in the main text, i.e. we compare the difference between clinical/demographic measures in the replication sample against predictions from models containing the task-based (including post-task self-report) measures vs. a model containing only an intercept. Note that the reason that $R^2_{OOS}$ values are sometimes negative and still significant is because random permutations led to even more negative $R^2_{OOS}$ values. The column 'against demogr. pred. only' shows the results of the pre-registered models, i.e. in addition to the task-based (including post-task self-report) measures, we also included other clinical measures; the control model then only contained these clinical measures. Here, given the increased correlations between the subscales in the replication sample, it is maybe not surprising that little variance remains unexplained that could be then linked to the task measures. Finally, we performed another reviewer suggested control analysis to rule out that our results could be confounded with bad performance on questionnaires ('Excluding participants with any errors on questionnaire quality'): instead of excluding participants based on showing on the check questions an average error higher than 1, we excluded them if they showed any error on any of the check questions. This strongly reduced the sample size (column '#participants' – less than half of participants). Yet, we find still the same results. No adjustments for multiple comparisons (but note that these analyses are complementary to those in the main text for which appropriate corrections for multiple comparisons were done through our hierarchical approach).

## Supplementary methods

### [1] Task instructions

Task instructions were presented as slides participants clicked through before doing practice trials, followed by a multiple choice quiz to check understanding.

| | |
|---|---|
| 1. Click through these task instructions using the buttons at the bottom. Warning: There will be a quiz at the end of the instructions to test whether you understand! | |
| 2. Throughout this game you will use 3 keyboard buttons: the left, up, and right arrows. | 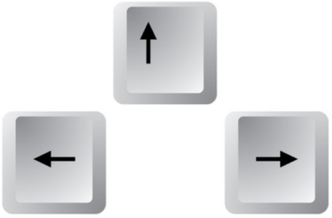 |
| 3. In this study you will play 20 rounds of the Undersea Adventure game. You play a little fish in the ocean. Your goal is to gain as much 'energy' as possible by eating delicious algae without getting eaten by predators. In the end of the study, the energy you've gathered will be translated into money. | 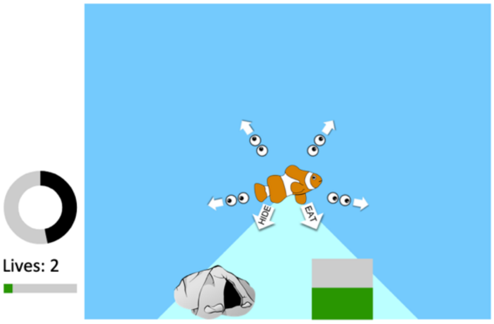 |
| 4. EAT using the RIGHT arrow key: You can gain energy by eating algae. Note how the amount of available algae varies randomly over time – so sometimes you gain more energy when you eat and sometimes less. The amount of energy you gain from eating will appear in the center of the screen (above shown as +29). | 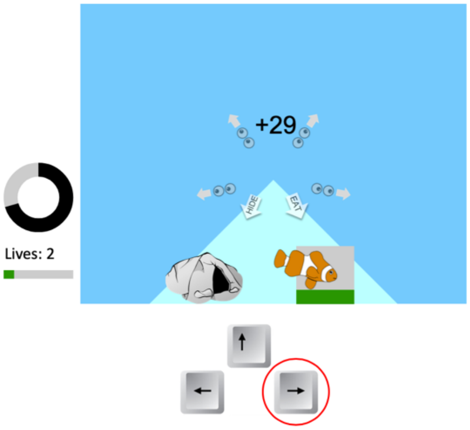 |
| 5. You can keep track of your energy by looking at how much green is filling the horizontal bar on the left side of the screen (see red circle). Whenever the bar is completely filled you gain an extra 'life'. You receive a bonus payment for each extra life you earn. Note: if you earn very few (<5) or negative extra | 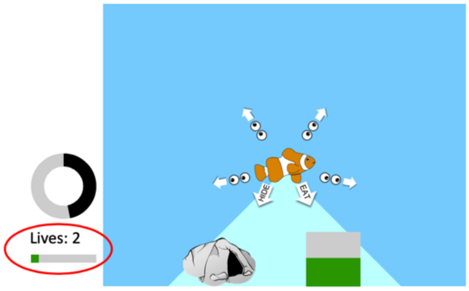 |

lives, your submission may not be approved due to low effort!

6. CHECK using the UP arrow key: Be careful - there may be a predator in the area! A predator can appear in any area of the surrounding water, and it can be hard to see predators through the murky water. To check whether a predator is swimming toward you, press the UP arrow key. This will allow your fish to see in a certain direction for a short time.

   When you are searching, the section of the environment that is revealed when you check will rotate each time you press the button. After you've discovered a predator, checking will always reveal the correct predator location. Predators swim towards you in a straight line until they reach you and eat you. Note: There will only be one predator at a time.

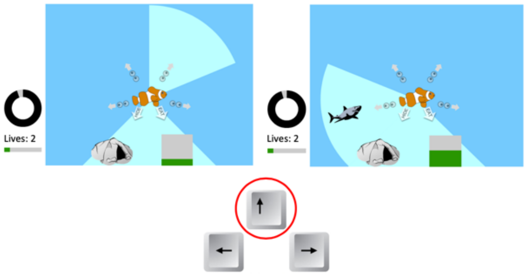


7. HIDE using the LEFT arrow key: You can avoid being eaten by clicking the LEFT arrow key to hide in a cave. While hiding, you can watch the predator reach the centre of the screen and then swim away again. However, you must be completely hidden by the time the predator arrives!

   Once the predator starts to swim away it will not return, so it is safe for you to come back out. To finish hiding, click the LEFT arrow key again. There may be a shorter or longer amount of time until another predator appears.

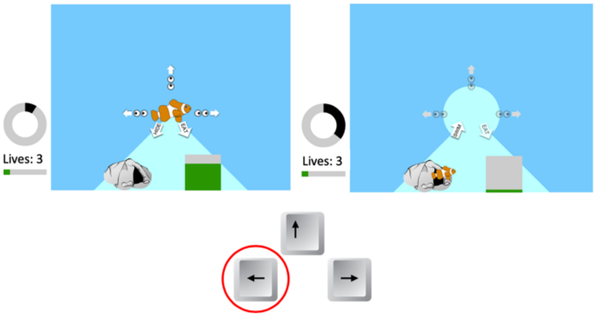


8. If you do not hide in time, the predator will catch you! Then you will lose 1 Life.

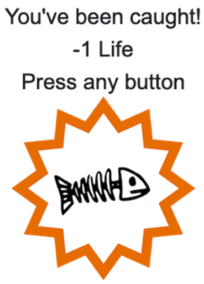

9. You will play 20 rounds, each lasting 90 seconds. The time left in the current round is shown by the circular clock on the left-hand side (see red arrow). When the current round is over, the grey area will be filled with black. You start each round with the same amount of health you had in the previous round.

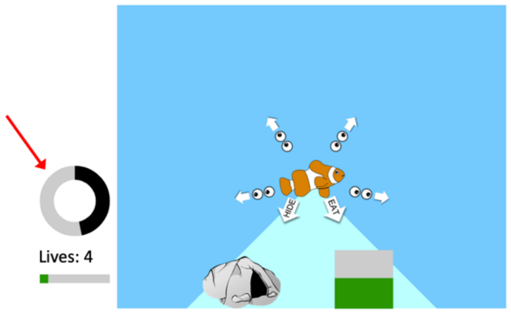


10. Whenever a new round starts, the ocean may become less clear (more areas to check) or more clear (fewer areas to check). When there are more areas to check, it means your fish can see less of the ocean each time it checks for predators. This can make it harder to tell whether there is a predator in the area and you might need to check more often.

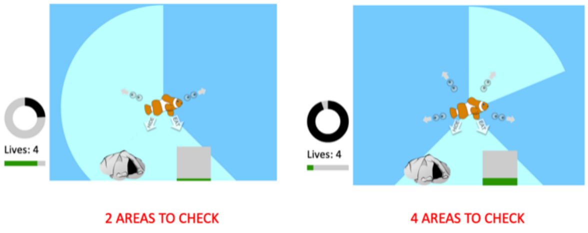


11. When you start a new round, the type of predators also change randomly. Different predators swim at different speeds. Jellyfish are the slowest, squid have a medium speed and sharks are the fastest.

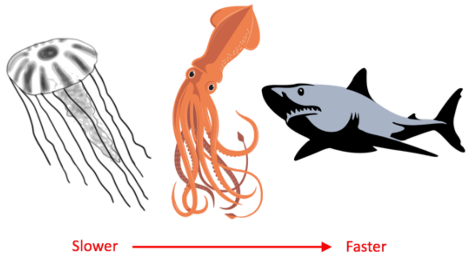


12. Before and after each round, you will be asked to answer two questions about your current mood. Click on the slider to rate your experience. When you click on the first slider, the second will appear.

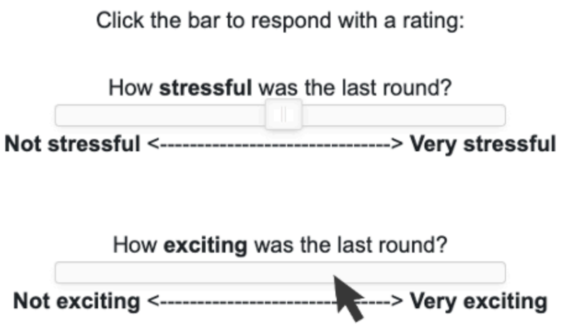


## [2] Multiple choice quiz

| Question | Text | Options | Correct answer |
|---|---|---|---|
| 1 | Right after you press a button (check, forage or hide), all the buttons appear grey for a short time. What does this mean? | a: "The game is not working."<br>b: "This is normal. The buttons are temporarily disabled so I don't press them twice accidentally. I wait until the buttons are no longer grey to choose my next action." | b |
| 2 | What happens when the fish is hiding? | a: "The predator disappears immediately." | b |

| | | | |
|---|---|---|---|
| | | b: "The predator keeps swimming to the middle of the screen and then leaves again. You are safe in the hiding place. | |
| 3 | Why is it important to eat as much algae and thus gain extra lives as possible? | a: "Extra lives become extra money for you at the end of this experiment."<br>b: "It is not important for the fish to gain as much health as possible." | a |
| 4 | What happens if the predator catches your fish? | a: "You lose a life (which means losing part of your bonus payment)."<br>b: "Nothing." | a |
| 5 | How can you tell how fast a predator is? | a: "Predators become faster as time progresses."<br>b: "The type of predator (shark, squid, or jellyfish) shows how fast it moves." | b |
| 6 | When can the predator catch the fish? | a: "The predator can catch the fish as soon as the predator reaches the centre of the screen, and only if the fish is not hiding."<br>b: "The predator can catch the fish when the fish is hiding."<br>c: "The predator can catch the fish even if the predator is still far away from the centre.” | a |
| 7 | Which picture shows the highest amount of food for the fish? | a: "A"<br>b: "B"<br>c: "C" | c |
| 8 | When a predator disappears after having reached the middle of the screen, how long until another predator appears? | a: "There may be a shorter or longer amount of time until another predator appears."<br>b: "Another predator will always appear exactly 5 seconds after the last one disappears."<br>c: "Another predator always appears immediately." | a |

## References


Berry, K. J., Johnston, J. E., & Mielke, P. W. (2019). *A Primer of Permutation Statistical Methods*. https://link.springer.com/book/10.1007/978-3-030-20933-9

Gronau, Q. F., Sarafoglou, A., Matzke, D., Ly, A., Boehm, U., Marsman, M., Leslie, D. S., Forster, J. J., Wagenmakers, E.-J., & Steingroever, H. (2017). A tutorial on bridge sampling. *Journal of Mathematical Psychology*, *81*, 80–97. https://doi.org/10.1016/j.jmp.2017.09.005

Morey, R., Rouder, J., Jamil, T., Urbanek, S., Forner, K., & Ly, A. (2022). *BayesFactor: Computation of Bayes Factors for Common Designs* (Version 0.9.12-4.4) [R]. https://richarddmorey.github.io/BayesFactor/

Rouder, J. N., Speckman, P. L., Sun, D., Morey, R. D., & Iverson, G. (2009). Bayesian t tests for accepting and rejecting the null hypothesis. *Psychonomic Bulletin & Review*, *16*(2), 225–237. https://doi.org/10.3758/PBR.16.2.225

Van Dam, N. T., Gros, D. F., Earleywine, M., & Antony, M. M. (2013). Establishing a trait anxiety threshold that signals likelihood of anxiety disorders. *Anxiety, Stress, & Coping*, *26*(1), 70–86. https://doi.org/10.1080/10615806.2011.631525

Vehtari, A., Gelman, A., & Gabry, J. (2017). Practical Bayesian model evaluation using leave-one-out cross-validation and WAIC. *Statistics and Computing*, *27*, 1413–1432.

Zhang, Z., & Yuan, K. H. (2018). Practical Statistical Power Analysis Using Webpower and R. *ISDSA Press*.

Zorowitz, S., Solis, J., Niv, Y., & Bennett, D. (2023). Inattentive responding can induce spurious associations between task behaviour and symptom measures. *Nature Human Behaviour*, 1–15.
