# Supplementary material for: Emotions and individual differences shape human foraging under threat
Source: Nat Ment Health. 2025 Mar 12;3(4):444–65. doi: 10.1038/s44220-025-00393-8 (PMC11978516; doi:10.1038/s44220-025-00393-8)
Supplement: Supplementary file 2 — Reporting Summary [file 44220_2025_393_MOESM2_ESM.pdf]

nature portfolio

Corresponding author(s): Jacqueline Scholl

Last updated by author(s): Nov 13, 2024

Reporting Summary

Nature Portfolio wishes to improve the reproducibility of the work that we publish. This form provides structure for consistency and transparency in reporting. For further information on Nature Portfolio policies, see our Editorial Policies and the Editorial Policy Checklist.

Statistics

For all statistical analyses, confirm that the following items are present in the figure legend, table legend, main text, or Methods section.

n/a	Confirmed	
☐	☒	The exact sample size (*n*) for each experimental group/condition, given as a discrete number and unit of measurement
☐	☒	A statement on whether measurements were taken from distinct samples or whether the same sample was measured repeatedly
☐	☒	The statistical test(s) used AND whether they are one- or two-sided<br>*Only common tests should be described solely by name; describe more complex techniques in the Methods section.*
☐	☒	A description of all covariates tested
☐	☒	A description of any assumptions or corrections, such as tests of normality and adjustment for multiple comparisons
☐	☒	A full description of the statistical parameters including central tendency (e.g. means) or other basic estimates (e.g. regression coefficient) AND variation (e.g. standard deviation) or associated estimates of uncertainty (e.g. confidence intervals)
☐	☒	For null hypothesis testing, the test statistic (e.g. *F*, *t*, *r*) with confidence intervals, effect sizes, degrees of freedom and *P* value noted<br>*Give P values as exact values whenever suitable.*
☐	☒	For Bayesian analysis, information on the choice of priors and Markov chain Monte Carlo settings
☒	☐	For hierarchical and complex designs, identification of the appropriate level for tests and full reporting of outcomes
☐	☒	Estimates of effect sizes (e.g. Cohen's *d*, Pearson's *r*), indicating how they were calculated

Our web collection on statistics for biologists contains articles on many of the points above.

Software and code

Policy information about availability of computer code

Data collection	Information copied from manuscript: The experiment was coded in javascript, using jQuery, GreenSock animation platform, noUiSlider animations code. Web applications JATOS [73] and Pavlovia [74] and the jsPsych library version 6.0 [75] were used for experiment hosting and data collection.
Data analysis	Information copied from manuscript: Analysis was performed in R version 4.3 [76] using Rstan version 2.26.13 [77], dplyr version 1.0.10 [78], tidyverse version 1.3.1 [79], ggpubr versions 0.4.0 and 0.5.0 [80], compareGroups version 4.5.1 [81], fuzzyjoin version 0.1.6 [82], data.Table version 1.14.6 [83], sjPlot version 2.8.12 [84], brms version 2.18.0 [85], [86], Stan version 2.26.13 [87], BayesFactor version 0.9.12-4.4 [88], Rcpp version 1.0.9 [89], [90], [91], stringr versions 1.5.0 [92], doParallel version 1.0.17 [93], foreach version 1.5.2 [94], loo version 2.5.1 [95], DescTools version 0.99.47 [96], , ggplot2 version 3.4.0 [97], bayesplot version 1.10.0 [98], openxlsx version 4.2.5.1 [99], lubridate version 1.8.0 [100], jsonlite version 1.8.0 [101], psych version 2.3.6 [102], paran version 1.5.2 [103], mice version 3.16.0 [104], mifa [105].<br><br>Custom code for this publication is available at 10.17605/OSF.IO/NTB5E

For manuscripts utilizing custom algorithms or software that are central to the research but not yet described in published literature, software must be made available to editors and reviewers. We strongly encourage code deposition in a community repository (e.g. GitHub). See the Nature Portfolio guidelines for submitting code & software for further information.

Data

Policy information about availability of data

All manuscripts must include a data availability statement. This statement should provide the following information, where applicable:

- Accession codes, unique identifiers, or web links for publicly available datasets
- A description of any restrictions on data availability
- For clinical datasets or third party data, please ensure that the statement adheres to our policy

All data (with removed identifiers) is available at 10.17605/OSF.IO/NTB5E

Research involving human participants, their data, or biological material

Policy information about studies with human participants or human data. See also policy information about sex, gender (identity/presentation), and sexual orientation and race, ethnicity and racism.

Reporting on sex and gender	Participants (50% female) were included irrespective of gender. Participants self-reported gender (options: female, male or other)
Reporting on race, ethnicity, or other socially relevant groupings	Race or ethnicity were not measured. We did not collect ethnicity data because such data are regarded as a "protected characteristic" under the UK Equality Act 2010. At the time of initiating the study our understanding was that protected characteristic data should only be collected when there are clear reasons to do so. We had no reason to suspect that the basic psychological processes that are the focus of our study should vary with ethnicity and so we did not collect ethnicity data. However highest education achieved and age was measured.
Population characteristics	Participants were recruited to be at least 18 years old (mean: 35). 402 participants were included after having completed another study, including pre-screening for an even spread of anhedonia and anxiety and acceptable task performance on two other cognitive tasks (see methods).
Recruitment	Participants were recruited via the online platform Prolific.co. Participants were informed about the content of the study at recruitment (cognitive tasks and mental health questionnaires). Thus it is likely that only participants interested in completing cognitive tasks and questionnaires would take part in the study. It is thus unclear how the results would translate to other populations (e.g. patients not well enough to understand task instructions or complete questionnaires).
Ethics oversight	Ethics approval for the study was given by Oxford University Central University Research Ethics Committee (CUREC) (Ref-numbers: R54722/RE001 and R77387/RE003).

Note that full information on the approval of the study protocol must also be provided in the manuscript.

Field-specific reporting

Please select the one below that is the best fit for your research. If you are not sure, read the appropriate sections before making your selection.

☐ Life sciences ☒ Behavioural & social sciences ☐ Ecological, evolutionary & environmental sciences

For a reference copy of the document with all sections, see nature.com/documents/nr-reporting-summary-flat.pdf

Behavioural & social sciences study design

All studies must disclose on these points even when the disclosure is negative.

Study description	Quantitative experimental study, not including any treatment manipulations. The design was cross-sectional.
Research sample	English-speaking volunteers recruited via the international website Prolific.co (age: 35+/- 11, gender: 50.6% female, 48% male, 1.4% other). The sample is thus only representative of commonly studied populations in psychological studies, not the population in general.
Sampling strategy	Sample size for the replication sample (i.e. sample reported in the article) was determined based on power calculation performed on a discovery sample and pre-registered. 402 participants were collected as a quota sample (see above), the remaining as a convenience sample.
Data collection	Participants completed the experiment on their own computer by following a link from the Prolific.co website. Responses were registered as mouse clicks and button presses. While in theory, participants could have contacted the experimenter before the experiment for clarification, in practice, all participants completed the experiment completely by themselves. Participants had no direct contact with the experimenter during or before study completion, thus experimenter bias was minimized.
Timing	11th of May 2022 until 7th of November 2022. The replication sample data was collected, but not analysed (other than the first ~10

Timing	participants checked for data recording problems) before pre-registration.
Data exclusions	Extensive data quality checks were used (copied from the methods): Inattentiveness and poor performance. Participants were excluded if they had too many (> 6) epochs in the experiment without any actions, if they checked too rarely (< 40 times), if they were caught too often (> 9 times), and if they earned less than 5 extra lives. To check attentiveness to questionnaires, three questions were repeated, and participants were excluded if the average absolute difference in repeated question scores was larger than one.<br>In addition, epochs were screened for measures of inattentiveness. Problematic epochs were either excluded from certain behavioural measures or excluded altogether. Epochs were excluded entirely if participants stayed in hiding at the beginning of the epoch for more than 2.2s, or if participants hid more than once. Behavioural measures were computed from epochs depending on the epoch outcome: if participants discovered the predator and successfully hid, epoch data was used to compute all behavioural measures; if participants discovered the predator but the block ended before they could hide, epoch data was used to compute all pre-discovery behavioural measures; if participants did not discover the predator in an incomplete epoch with at least 10s of data, epoch data was used to compute the 'rate of inactive button presses' and 'time to first action' measures; if participants did not discover the predator in an incomplete epoch with less than 10s of data, epoch data was used to compute only 'time to first action'; if participants were caught by the predator, all within-epoch behavioural measures were computed but the data was not used to compute measures across epochs (e.g. time to first action after the predator leaves).<br><br>Technical errors. Participants were excluded if they reported technical problems with the task, including the predator or the reward animations showing incorrectly. In addition, data was screened for unreported technical errors. If errors were detected in a predator epoch, that epoch and all subsequent ones in that block were removed from data analysis. If too many epochs were removed, the participant was excluded. The cut-off for what was considered too many removed epochs was determined based on the replication sample, such that at most 2.5% of participants were removed by a single behavioural measure.<br>Two indicators of technical errors were used to exclude data: 1) Any actions that happened in an epoch after a predator caught the participant were removed (e.g. a participant might have pressed the 'forage' button while on the screen showing they had been caught). 2) After each action, there was an enforced delay before the buttons became active again (1.5 sec for forage, 0.5 sec for check). Technical problems sometimes meant that this was not respected. We used a difference by more than 100 msec as a cut-off for exclusion.<br><br>Questionnaire quality. Before completing the questionnaires, participants were reminded that this was an important part of the study and that some questions would repeat to check for data quality. In the end of the questionnaires, participants were shown three repeat questions: SHAPS Q4 ("I would find pleasure in my hobbies and pastimes"), AMI Q13 ("I feel awful if I say something insensitive"), OCRI Q2 ("I check things more often than necessary"). These questions were selected based on subjective judgements that they were questions participants were likely to have a strong opinion about. In fact, all of these questions showed a strong leftward skew with the lowest (i.e. least psychiatrically symptomatic) answer being the most frequently picked, meaning that – in contrast to questions where most answers are scored in the middle of the range – divergences between the question repeats was more easily detectable. We computed for each participants the average of the absolute difference between their repeat answers for each pair of questions.<br><br>In the replication sample, we thus excluded 133 participants (17.2%) in total.
Non-participation	Once participants had started with the computerized tasks, none dropped out.
Randomization	Participants were not allocated to groups.

Reporting for specific materials, systems and methods

We require information from authors about some types of materials, experimental systems and methods used in many studies. Here, indicate whether each material, system or method listed is relevant to your study. If you are not sure if a list item applies to your research, read the appropriate section before selecting a response.

Materials & experimental systems

n/a	Involved in the study
☒	☐ Antibodies
☒	☐ Eukaryotic cell lines
☒	☐ Palaeontology and archaeology
☒	☐ Animals and other organisms
☒	☐ Clinical data
☒	☐ Dual use research of concern
☒	☐ Plants

Methods

n/a	Involved in the study
☒	☐ ChIP-seq
☒	☐ Flow cytometry
☒	☐ MRI-based neuroimaging
